# Programmable Encapsulation Enables On‐Demand Proliferation of Therapeutic Bacteria for Potent Cancer Immunotherapy

**DOI:** 10.1002/advs.76706

**Published:** 2026-07-23

**Authors:** Jianhui Yang, Ao Peng, Shuaiqiang Li, Yaning Huang, Jian Yan, Leyuan Wang, Zhihui Zhu, Fei‐Long Liu, Erbao Bian, Yang Liu, Dasheng Tian, Fenghe Li, Qi Liu

**Affiliations:** ^1^ School of Pharmacy Anhui Medical University Hefei China; ^2^ Department of Orthopedics The Second Affiliated Hospital of Anhui Medical University Hefei China; ^3^ State Key Laboratory of Medicinal Chemical Biology Key Laboratory of Functional Polymer Materials Ministry of Education Nankai University Tianjin China

**Keywords:** bacterial capsule, cancer immunotherapy, immune checkpoint blockade, l‐arginine supplementation, live bacterial therapeutic

## Abstract

Engineered bacteria have emerged as a promising therapeutic modality but face safety risks and delivery challenges in clinical practice. Herein, we develop a programmable encapsulation technology that formulates individual bacteria with a thin formulation layer of cross‐linked polymers, which confers live bacteria with reduced immunogenicity and restricted proliferation in healthy organs. To leverage these benefits, an engineered Escherichia coli Nissle strain capable of converting tumor‐accumulated ammonia into L‐arginine and secreting soluble programmed cell death protein 1 (sPD‐1) was encapsulated to synthesize a degradable bacterial capsule, optimizing both biosafety and delivery processes while preserving therapeutic function. Upon reaching the tumors, matrix metalloproteinase‐2 triggers bacterial release and local proliferation, achieving L‐arginine‐driven tumor immune microenvironment modulation and sustained PD‐L1 blockade, ultimately initiating robust antitumor immune responses. Overall, this programmable encapsulation platform tackles both safety risks and delivery challenges of bacterial medicines, broadening the clinical application prospects of live bacterial therapeutics.

## Graphical abstract

A programmable encapsulation technology is developed to reduce bacterial immunogenicity and proliferation after systemic administration. The cross‐linked polymer networks around individual bacteria enable immunogenic shielding and permit selective proliferation in tumors, allowing the bacteria to convert tumor‐accumulated ammonia into L‐arginine and secrete soluble programmed cell death protein 1, thereby achieving effective tumor microenvironment modulation and PD‐1/PD‐L1 pathway blockade.

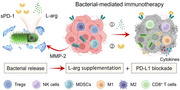

## 1 Introduction

Recent advances in synthetic biology and genetic engineering have spurred growing interest in harnessing microbes for disease treatment [1, 2, 3]. Many microbes hold the innate ability to sense and respond to the physiological microenvironment, enabling targeted colonization of pathological tissues to deliver or locally secrete therapeutic agents [4, 5, 6]. For instance, certain bacterial strains exhibit preferential tumor tropism due to the immunosuppressive and hypoxic tumor microenvironment (TME), rendering them attractive live biotherapeutic products in cancer treatment [7, 8]. In addition, intratumoral bacterial colonization has been shown to augment tumor immunogenicity, eliciting potent antitumor immunity [9, 10, 11]. Despite a multitude of benefits, live bacterial therapeutics face severe challenges due to host toxicity, which restricts tolerable doses and compromises therapeutic outcomes, and ultimately leads to the discontinuation of several early‐phase clinical trials [12, 13, 14]. In particular, the propensity of systemically administered bacteria to persistently colonize and proliferate in healthy tissues, including liver, spleen, and hypoxic stem cell niches, may raise serious safety concerns [15, 16, 17, 18, 19]. To minimize damage to healthy tissues, it is crucial to mitigate the biotoxicity of live bacterial therapeutics.

One strategy to mitigate immunogenicity and biotoxicity of live bacterial therapeutics involves the knockout of key virulence genes to generate attenuated strains, exemplified by engineered *Escherichia coli*, *Listeria*, and *Clostridium* [20, 21, 22, 23]. However, genetic knockouts struggle to achieve an optimal balance between safety and effectiveness, often resulting in irreversible attenuation and diminished colonization capacity, as validated by the failure of clinical trials of attenuated *Salmonella typhimurium* [13, 24, 25]. Although nonsystemic localized injection may bypass this limitation, its suboptimal efficacy and limited applicability restrict its use in many disease settings [26]. Surface modification has been proven to be effective in engineering drug delivery vehicles with reduced immunogenicity, thereby reducing their elimination by the mononuclear phagocytic system (MPS) and extending systemic circulation [27, 28]. Building on this principle, surface functionalization of bacteria with biocompatible coatings, including polydopamine [29]. lipids [30, 31], chitosan [32], and nanoparticles [33], represents an alternative strategy to mitigate bacterial toxicity while preserving their biological activity [34, 35, 36]. Despite these advancements, current studies have made limited progress in controlling bacterial proliferation after systemic administration. Undesirable colonization and persistent proliferation of bacteria in healthy organs may induce a prolonged toxic response, as validated by a study showing detectable *Salmonella* in normal organs of crab‐eating monkeys for over 40 days after treatment [37, 38]. Therefore, a strategy capable of concurrently mitigating bacterial immunogenicity and restricting their proliferation in non‐targeted organs is critically needed to advance the clinical translation of live bacterial therapeutics.

**FIGURE 1:**
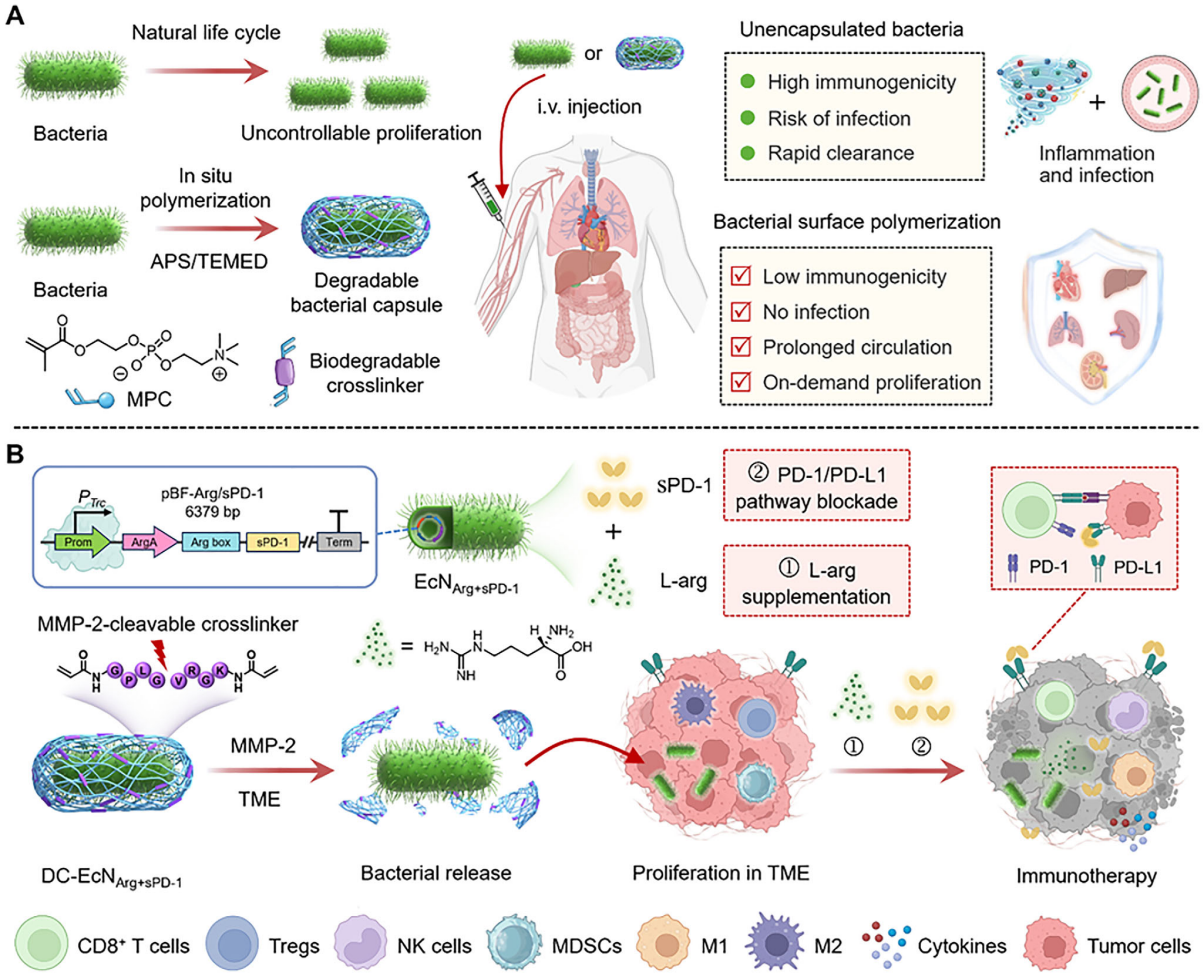
A universal encapsulation technology enables on‐demand proliferation of therapeutic bacteria for cancer immunotherapy. (A) In situ formation of cross‐linked polymer networks on bacterial surface to mitigate bacterial immunogenicity and restrict its proliferation in healthy organs. (B) Schematic illustration of engineered bacterial strains (EcN_Arg_+sPD‐1), in vivo delivery processes, and antitumor mechanisms. Following systemic administration, DC‐EcN_Arg_+sPD‐1 achieves TME‐selective bacterial release in response to intratumoral MMP‐2, enabling localized proliferation, sustained production of L‐arg and sPD‐1 to synergistically activate antitumor immune responses. A and B were created with BioRender.com.

For a proof‐of‐concept validation, we chose L‐arginine (L‐arg) as a therapeutic target for its pivotal role in modulating TME and T cell immune responses [39, 40, 41]. Increasing intratumoral L‐arg levels can significantly potentiate immune checkpoint blockade therapies [42, 43]. However, systemic administration requires impractically high doses (about 150 g L‐arg per patient daily), while intratumoral injection usually leads to rapid diffusion and poor local retention [44, 45]. Engineered bacteria overcome this limitation by locally producing L‐arg in the TME, ensuring sustainable therapeutic availability. However, current strategies remain constrained by bacterial safety profiles that necessitate suboptimal local administration routes. Herein, we developed a platform technology that formulates individual bacteria with a thin layer of cross‐linked polymer networks. This polymer coating confers triple functionality, including immunogenic surface shielding, prolonged blood circulation, and on‐demand bacterial proliferation after systemic administration, addressing both biotoxicity and delivery challenges without compromising biological activity (Figure 1A). To demonstrate this, an *Escherichia coli* Nissle 1917 (EcN) was engineered to constantly convert tumor‐accumulated ammonia into L‐arg (EcN_Arg_), and then encapsulated to form a bacterial capsule (C‐EcN_Arg_). Compared to unencapsulated EcN_Arg_, C‐EcN_Arg_ showed lower immunogenicity and negligible damage of healthy organs. In addition, C‐EcN_Arg_ prevented rapid MPS clearance during blood circulation and undesirable bacterial proliferation in healthy organs while preserving their tumor colonization capacity. Ultimately, C‐EcN_Arg_ dramatically elevated intratumoral L‐arg level and synergized with anti‐programmed death‐ligand 1 (αPD‐L1) to initiate potent antitumor immune responses.

A prominent advantage of live bacterial therapeutics over conventional drugs lies in their ability to proliferate in pathological tissues, enabling in situ therapeutic amplification [7, 46]. In this study, all monomers and cross‐linkers for bacterial encapsulation are commercially accessible and readily customizable, allowing for systematic optimization of bacterial capsule properties to achieve lesion‐selective bacterial release and proliferation. To achieve on‐demand bacterial proliferation within the tumors, we synthesized an enzyme‐responsive cross‐linker that can be cleaved by matrix metalloproteinase 2 (MMP‐2), a TME‐specific physiological stimulus. EcN was engineered to simultaneously produce L‐arg and secrete soluble PD‐1 (sPD‐1) (EcN_Arg+sPD‐1_), combining L‐arg supplementation and immune checkpoint blockade in a single strain to streamline clinical costs and implementation (Figure 1B). Next, EcN_Arg+sPD‐1_ was encapsulated using an MMP‐2‐cleavable cross‐linker to form a degradable bacterial capsule (DC‐EcN_Arg+sPD‐1_). Unlike a non‐degradable bacterial capsule, DC‐EcN_Arg+sPD‐1_ enables selective bacterial release and localized proliferation. Accordingly, EcN_Arg+sPD‐1_ achieves dual antitumor mechanisms: (i) L‐arg‐driven modulation of the tumor immune microenvironment and (ii) effective PD‐1/PD‐L1 pathway blockade‐mediated T cell activation. Together, this work establishes a programmable bacterial encapsulation strategy to mitigate bacterial biotoxicity and selectively proliferate at lesion sites, providing insights for advancing the clinical application of live bacterial therapeutics.

## 2 Results and Discussion

### 2.1 EcN_Arg_ Synergizes With αPD‐L1 to Activate Antitumor Immunity

**FIGURE 2:**
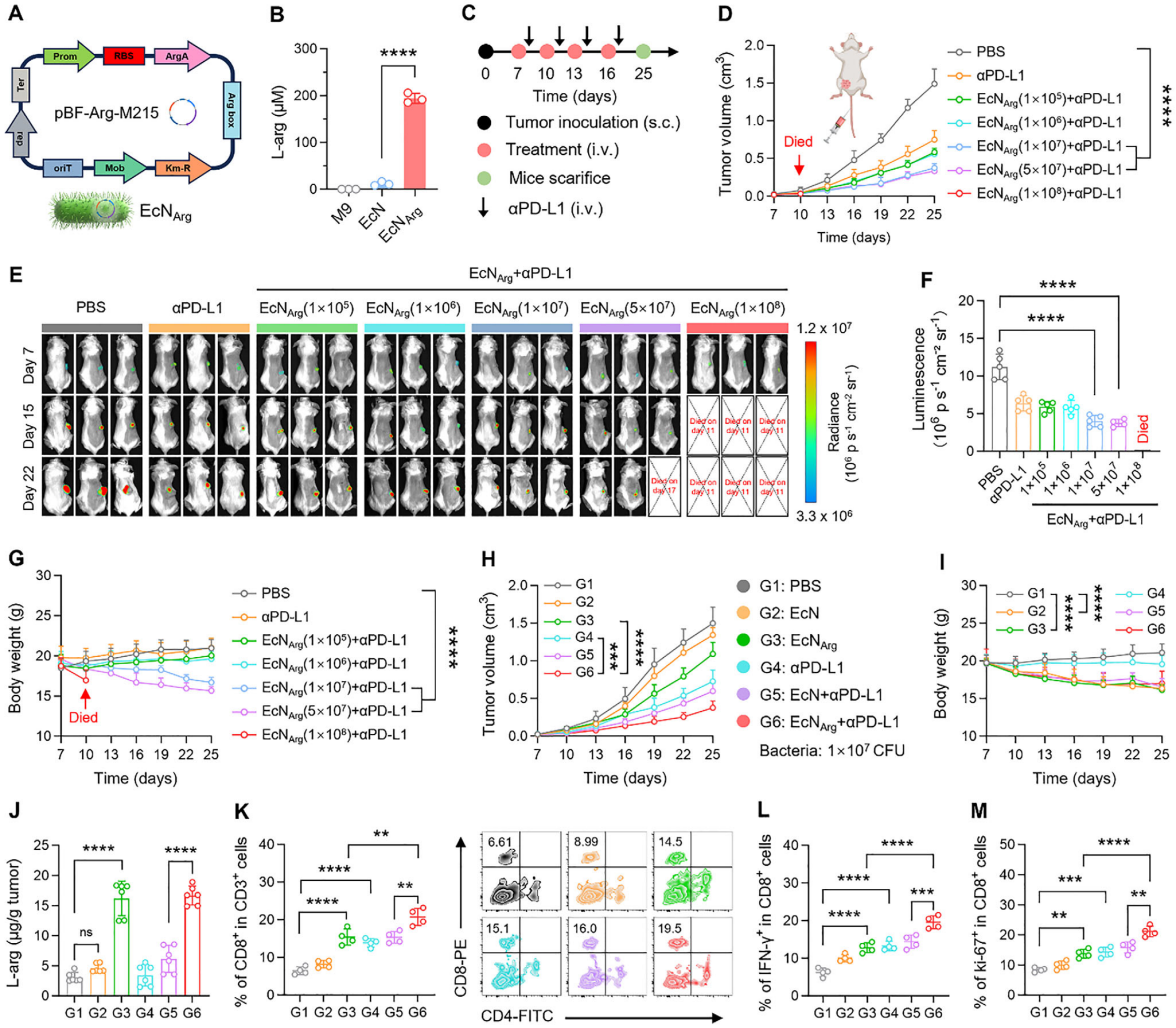
Synergistic antitumor effect of EcN_Arg_ and αPD‐L1. (A) Plasmid map for constructing EcN_Arg_. (B) Non‐engineered EcN and EcN_Arg_ were cultured in a medium containing 5 mm NH_4_Cl as the only nitrogen source and then incubated at 37°C for 4 h. L‐arg levels in the supernatant of the medium was measured using a L‐arg detection kit. (C) Schematic illustration for evaluating the antitumor effects of EcN_Arg_+αPD‐L1 in 4T1‐bearing mice. (D) The antitumor effects of the combination of various doses of EcN_Arg_ and αPD‐L1. (E) In vivo bioluminescence images of 4T1‐bearing mice after different treatments. (F) Bioluminescence quantitative analysis of 4T1‐bearing mice after different treatments. (G) Changes in mouse body weight during combination therapy with varying doses EcN_Arg_ and αPD‐L1. (H) Tumor growth curves of mice treated with different formations. (I) Changes in mouse body weight after different treatments. (J) Intratumoral L‐arg levels after different treatments. (K–M) Flow cytometry analysis of tumor‐infiltrating CD8^+^ (K), IFN‐γ^+^ CD8^+^ (L), and Ki‐67^+^ CD8^+^ (M) T cells. Data in (B) are presented as means ± s.d. from three independent experiments (*n*  = 3). Data in (F) are presented as means ± s.d. from five independent experiments (*n* = 5). Data in (K–M) are presented as means ± s.d. from four independent experiments (*n* = 4). Data in D and (G–J) are presented as means ± s.d. from six independent experiments (*n* = 6). Statistical analysis in (B) was performed using Student's t test. Statistical analysis in (F) and (J–M) were performed using one‐way ANOVA followed by Tukey's test with multiple comparisons. Statistical analysis in (D) and (G–I) was performed using two‐way ANOVA followed by Sidak's test with multiple comparisons. The significant levels are shown as *p < 0.05, **p < 0.01, ***p < 0.001, and ****p < 0.0001.

L‐arg supplementation has emerged as a promising strategy to activate T cells for cancer immunotherapy, particularly in combination with immune checkpoint blockade therapies [43]. However, the clinical translation of L‐arg supplementation faces two major challenges: (i) systemic administration requires prohibitively high doses and (ii) intratumoral injection leads to poor local retention due to rapid diffusion [44]. To address these limitations, an engineered EcN (EcN_Arg_) was constructed as a living therapeutic platform to convert tumor‐accumulated ammonia (byproduct of glutamine metabolism) into L‐arg [47], ensuring sustainable therapeutic availability. EcN_Arg_ was constructed by introducing a functional plasmid (pBF‐Arg‐M215) encoding N‐acetylglutamate synthase (ArgA) and Arg box (detailed gene sequences were illustrated in). ArgA is the first enzyme in the pathway of arginine biosynthesis, and the Arg box can titrate the ArgR repressor protein. The map and successful construction of pBF‐Arg‐M215 are presented in Figure 2A and Figure S1A. To assess the production of L‐arg, EcN_Arg_ was cultured in a medium containing ammonium chloride as the only nitrogen source. As shown in Figure 2B, compared to wild EcN, EcN_Arg_ produced a higher level of L‐arg (16.3‐fold over that of wild EcN) after 4 h cultivation. Next, we investigated whether EcN_Arg_ can synergize with PD‐1/PD‐L1 pathway blockade immunotherapy. All experimental protocols were conducted within Anhui Medical University guidelines for animal research and approved by the Animal Care and Use Committee (LLSC20230877). A breast cancer model was constructed by subcutaneously injecting 1 × 10^6^ 4T1 cells. Then, 4T1‐bearing mice were treated with EcN_Arg_ and αPD‐L1 via tail vein as indicated in Figure 2C. As shown in Figure 2D–F and Figure S2, EcN_Arg_ significantly enhanced the efficacy of αPD‐L1‐mediated immune checkpoint blockade therapy, demonstrating the highest antitumor activity at a dose of 1 × 10^7^ colony‐forming units (CFU). In contrast, the mice treated with EcN_Arg_ alone (1 × 10^7^ CFU) and wild EcN (1 × 10^7^ CFU) plus αPD‐L1 (EcN+αPD‐L1) displayed significantly lower antitumor efficacy (Figure 2H and Figure S3). By overcoming metabolic immunosuppression and providing T cell‐supportive L‐arg, EcN_Arg_ may synergize with αPD‐L1 to activate robust T cell‐mediated antitumor immune responses [39, 43]. To elucidate the therapeutic mechanism of the combination of EcN_Arg_ and αPD‐L1, intratumoral L‐arg levels were first assessed. As expected, significantly higher intratumoral L‐arg levels were detected after EcN_Arg_ and EcN_Arg_+αPD‐L1 treatments (Figure 2J), indicating the effective EcN_Arg_‐mediated L‐arg production in tumors. Ultimately, we analyzed the T cell subsets in TME. As shown in Figure 2K, EcN_Arg_+αPD‐L1 treatment induced a higher proportion of tumor infiltration of cytotoxic T lymphocytes (CTLs, CD8^+^ CD3^+^ CD45^+^ cells) compared to that of EcN+αPD‐L1 treatment. Moreover, EcN_Arg_+αPD‐L1 improved the proliferation (Ki‐67^+^ CD8^+^) and killing (IFN‐γ^+^ CD8^+^) activity of CTLs (Figure 2L, M, and Figure S4), indicating the potential of EcN_Arg_ in enhancing αPD‐L1‐mediated antitumor T cell immune responses.

A key challenge in current bacterial therapeutics is toxicity. To this end, we continuously monitored the mouse body weight during treatment. As shown in Figure 2G, I, at the high doses of EcN (1 × 10^7^ CFU) and EcN_Arg_ (exceeding 1 × 10^7^ CFU), the mouse body weight rapidly dropped, and even all mice died at a dose of 1 × 10^8^ CFU. These results underscore the severe toxicity of live bacterial therapeutics, including those derived from non‐pathogenic *Escherichia coli* strains. We further conducted a comprehensive safety evaluation on BALB/c mice (Figure S5A). Major organs and blood were collected for histopathological, blood biochemical, and routine blood analysis after treatments. Analysis revealed significant alterations in multiple hematological and biochemical parameters. As shown in Figure S5B, the mice treated with bacterial formulations showed significant elevation in WBC, PLT, and NEUT counts, implying systemic inflammation and bacterial infection. In addition, noticeable fluctuations in liver (ALT, AST, and GGT) and kidney (UREA and CREA) biomarkers indicate serious hepatorenal toxicity after live bacterial therapeutics. Histopathological examination (hematoxylin and eosin (H&E) staining) also exhibited hepatic inflammatory cell infiltration (Figure S6), corroborating the hepatotoxicity of bacteria at a high dose. Collectively, these results emphasize the urgent need to mitigate safety risks of live bacterial therapeutics for facilitating their clinical translation.

### 2.2 Preparation and Characterization of Bacterial Capsule

Surface polymerization has been extensively explored in our previous studies for protein encapsulation, which can effectively shield protein immunogenicity while preserving small molecule permeability [48, 49, 50, 51]. To mitigate the biotoxicity of living bacterial medicines, we developed a bacterial encapsulation technology through an in situ free‐radical polymerization method in this work. This method can generate cross‐linked polymer networks around individual bacteria, designed to simultaneously: (i) mitigate immunogenicity while preserving permeability to essential small molecules and (ii) restrict bacterial proliferation in healthy organs after systemic administration.

**FIGURE 3:**
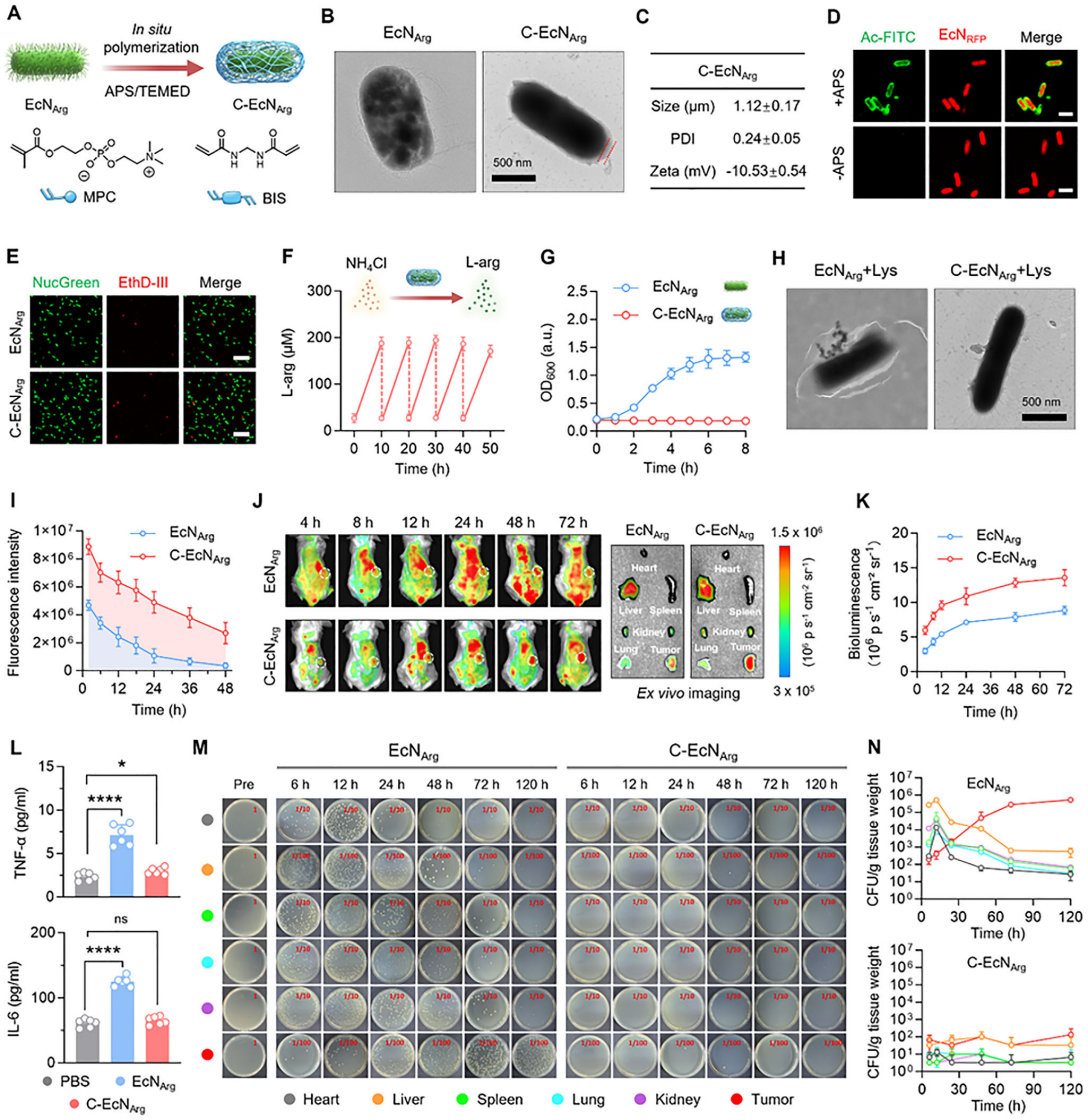
Synthesis and characterization of bacterial capsule. (A) Schematic illustration of the fabrication of C‐EcN_Arg_. (B) TEM characterization of EcN_Arg_ and C‐EcN_Arg_; Scale bar: 500 nm. (C) Hydrodynamic particle size, polydispersity index (PDI), and zeta potential of C‐EcN_Arg_. (D) Confocal laser scanning microscope (CLSM) images for the preparation of EcN_RFP_ with or without initiator (APS). EcN was indicated by RFP (red), and Ac‐FITC (green) was added as a monomer; Scale bar: 1 µm. (E) NucGreen (living bacteria, green) and EthD‐III (dead bacteria, red) staining images of EcN_Arg_ and C‐EcN_Arg_; Scale bars: 10 µm. (F) C‐EcN_Arg_ was cultured in a medium containing 5 mM NH_4_Cl as the only nitrogen source at 37°C. L‐arg was measured using a L‐arg detection kit. Culture medium was replaced with fresh medium every 10 h within 50 h. (G) Time‐dependent growth curves of EcN_Arg_ and C‐EcN_Arg_ cultured in LB medium at 37°C. (H) TEM images of EcN_Arg_ and C‐EcN_Arg_ after lysozyme (50 µg mL^−1^) treatment. (I) Pharmacokinetics of EcN_Arg_ and C‐EcN_Arg_ within 48 h. EcN_Arg_ was labeled with Cy5.5. (J) In vivo fluorescence imaging and *ex vivo* fluorescence images of major organs and tumors in mice following intravenous injection of EcN_Arg_ and C‐EcN_Arg_. EcN_Arg_ was labeled with Cy5.5. (K) Quantitative analysis of tumor fluorescence intensity. (L) Inflammatory factor levels (TNF‐α and IL‐6) in mouse serum following intravenous injection of PBS, EcN_Arg_, and C‐EcN_Arg_, respectively. (M) Representative photographs of LB agar plates spread with tissue homogenate of major organs and tumors at different time points after intravenous injection of EcN_Arg_ and C‐EcN_Arg_. (N) Bacterial counts analysis in normal organs and tumor tissues. Data in (F), (G), (I), (K), and (N) are presented as means ± s.d. from three independent experiments (*n* = 3). Data in (L) are presented as means ± s.d. from six independent experiments (*n* = 6). Statistical analysis was performed using one‐way ANOVA followed by Tukey's test with multiple comparisons. The significant levels are shown as ns > 0.05, *p < 0.05, and ****p < 0.0001.

To reduce safety risks, EcN_Arg_ capsules (C‐EcN_Arg_) were synthesized through an in situ free‐radical polymerization method, forming a thin polymer layer composed of acrylamide (AAm), 2‐methacryloyloxyethyl phosphorylcholine (MPC), and N, N'‐methylenebisacrylamide (BIS) on the surface of individual EcN_Arg_ (Figure 3A). MPC allows the formation of zwitterionic polymer layers around single bacteria that minimize plasma protein adsorption and macrophage uptake, thereby improving serum stability and prolonging blood circulation [52]. The successful synthesis of C‐EcN_Arg_ was characterized by transmission electron microscopy (TEM) and dynamic light scattering (DLS) measurements. Compared to unencapsulated EcN_Arg_, a distinct polymer layer, increased particle size, and zeta potential were observed from C‐EcN_Arg_, and indicating that the thickness of the polymer layer is approximately 30 nm (Figure 3B, C, and Figure S7). To further verify successful polymerization on the bacterial surface, EcN encoding red fluorescent protein (EcN_RFP_) and green fluorescent monomer (acetylated FITC, Ac‐FITC, synthesis detailed in Figure S8) were used to synthesize C‐EcN_RFP_. Intense green fluorescence on the bacterial surface confirmed the successful formation of polymer networks (Figure 3D), whereas no detectable polymer network (green) was observed in the control group without initiator (ammonium persulfate, APS). We also assessed the stability of bacterial capsule in different physiological shear forces and pHs, and found that the surface polymer layer of C‐EcN possessed excellent structural stability against fluid shear stress and pH variations (Figure S9). NucGreen/EthD‐III staining images showed that bacterial encapsulation did not significantly impair their activity, and C‐EcN_Arg_ retained more than 90% viability relative to unencapsulated EcN_Arg_ (Figure 3E and Figure S10A). To evaluate the potential of C‐EcN_Arg_ in sustainably producing L‐arg, C‐EcN_Arg_ was cultured in a medium containing 5 mm NH_4_Cl as the only nitrogen source. Culture medium was replaced with fresh medium every 10 h. As shown in Figure 3F, C‐EcN_Arg_ exhibited considerable metabolic activity and continuously converted NH_4_Cl to L‐arg within 50 h. To investigate the potential of engineered bacteria in activating T cells, T cells were extracted from the spleen of C57BL/6 mouse and incubated with the supernatant of EcN_Arg_ and C‐EcN_Arg_. Free L‐arg and the supernatant of wild‐type EcN were employed as comparative groups. The result indicates EcN_Arg_ and C‐EcN_Arg_ elicited similar and robust T‐cell proliferation compared to free L‐arg, indicating the potential of bacterial‐driven L‐arg in activating T‐cell proliferation (Figure S11A). In contrast, the supernatant of wild‐type EcN showed little regulatory effect on T cell proliferation. We also assessed the IFN‐γ secretion levels of T cells after different treatments. As shown in Figure S11B, the supernatant of EcN_Arg_ and C‐EcN_Arg_ significantly promoted the IFN‐γ secretion of T cells. These findings demonstrate that surface polymerization achieves effective bacterial encapsulation while preserving their metabolic function.

A principal assumption for encapsulating bacteria with cross‐linked polymer networks is to spatially limit bacterial proliferation. To demonstrate this, we first compared the growth curves of unencapsulated EcN_Arg_ and C‐EcN_Arg_. As shown in Figure 3G, C‐EcN_Arg_ showed near‐complete growth inhibition relative to unencapsulated EcN_Arg_. For direct observation, EcN_RFP_ and C‐EcN_RFP_ were plated on Luria‐Bertani (LB) agars, respectively. After 10 h incubation at 37°C, C‐EcN_RFP_ plated on LB agar showed little fluorescence (Figure S10B), confirming the bacterial proliferation inhibition post‐encapsulation. Furthermore, the lysozyme killing experiment showed severe morphological damage to EcN_Arg_ at 50 µg mL^−1^, whereas C‐EcN_Arg_ remained largely intact (Figure 3H). This phenomenon may arise from either limited access of lysozyme to the bacterial surface or structural stabilization of cross‐linked polymer networks. To analyze this, we assessed C‐EcN_Arg_ viability following lysozyme exposure and detected no significant decrease in bacterial survival (Figures S10C, D), indicating that cross‐linked polymer networks can protect bacterial activity by limiting the contact between lysozyme and the bacterial surface.

To evaluate the immunogenicity and biosafety of C‐EcN_Arg_ in vivo, we compared systemic inflammatory responses and organ toxicity between EcN_Arg_ and C‐EcN_Arg_‐treated BALB/c mice following systemic administration. Enzyme‐linked immunosorbent assays (ELISA) revealed much lower serum levels of proinflammatory cytokines (TNF‐α and IL‐6) in C‐EcN_Arg_‐treated mice compared to EcN_Arg_‐treated mice (Figure 3L), indicating improved biocompatibility with surface polymerization‐mediated immunoshielding. Moreover, we performed a comprehensive analysis of blood and organ samples after systemic administration, including histopathological examination, blood biochemical and routine blood assays. Although unencapsulated EcN_Arg_ induced obvious inflammation, bacterial infection, and hepatorenal injury, C‐EcN_Arg_ treatment showed stable inflammatory indicators, normal liver and kidney function (Figure S12), demonstrating the potential of surface polymerization in reducing the toxicity of living bacteria. The mitigated immunogenicity of C‐EcN_Arg_ indicates extended blood circulation. To validate this, Cy5.5‐labeled EcN_Arg_ and C‐EcN_Arg_ were intravenously injected into the mice. C‐EcN_Arg_ (18.8 ± 1.4 h) exhibited a prolonged in vivo half‐time compared to unencapsulated EcN_Arg_ (7.05 ± 0.82 h) (Figure 3I), indicating the circulatory benefits of bacterial encapsulation. Biodistribution analysis revealed a higher tumor accumulation of C‐EcN_Arg_ (in vivo and *ex vivo* fluorescence imaging, Figure 3J, K), which is mainly due to reduced immune clearance and preserved tumor colonization ability of EcN following surface polymerization. Ultimately, we investigated bacterial proliferation in vivo following systemic administration. Colony counting assays of major organs and tumors showed that EcN_Arg_ that accumulated in healthy organs (e.g., heart, liver, and spleen) and tumors remained capable of proliferation. In contrast, systemically administrated C‐EcN_Arg_ was unable to proliferate in these tissues (Figure 3M, N). This stark contrast demonstrates that surface cross‐linked polymer networks can restrict in vivo bacterial proliferation through spatial confinement.

### 2.3 Synergistic Anti‐tumor Efficacy of C‐EcN_Arg_ and αPD‐L1

**FIGURE 4:**
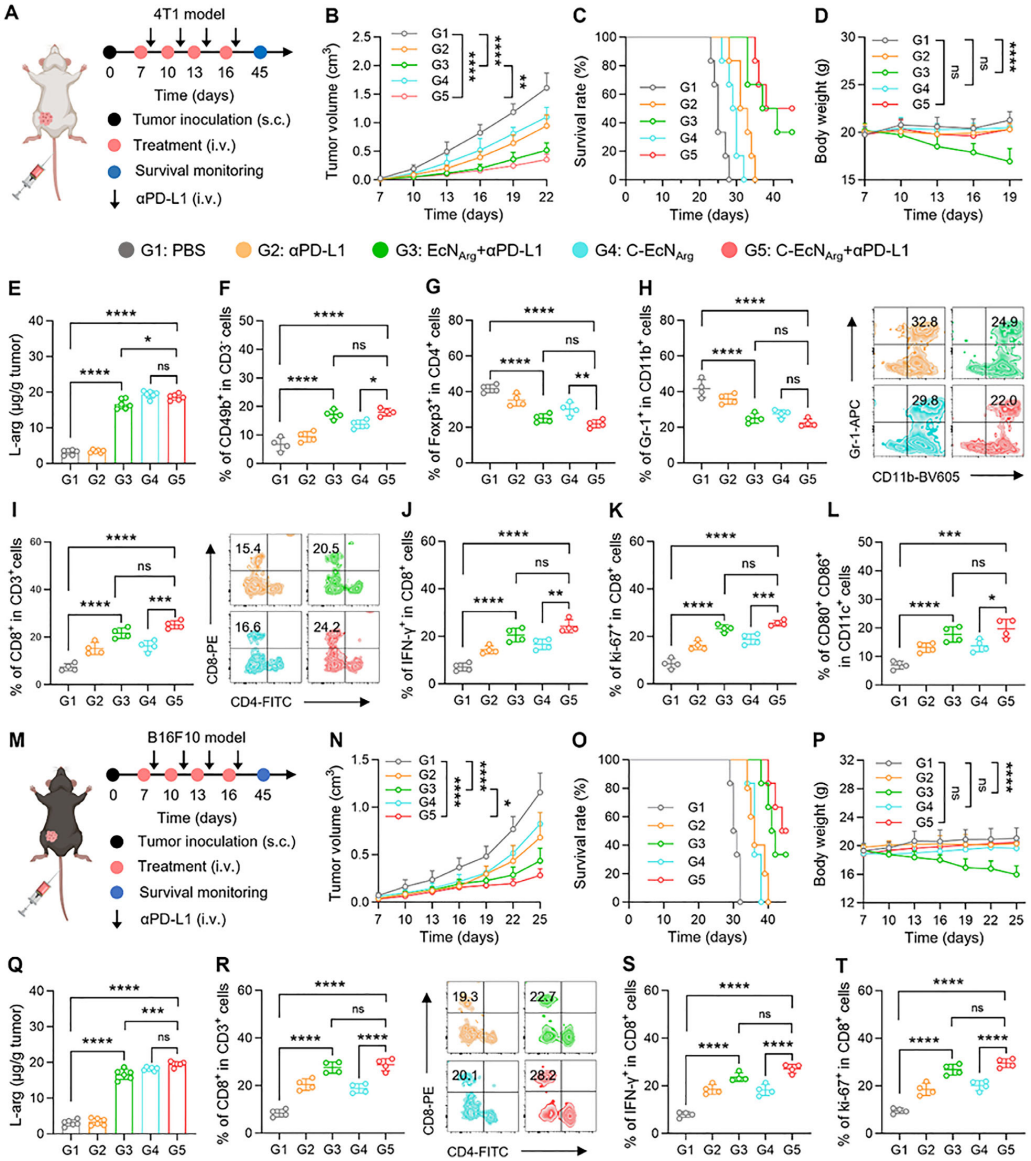
Synergistic antitumor efficacy of C‐EcN_Arg_ combined with αPD‐L1. (A) Schematic illustration for evaluating the antitumor effect of C‐EcN_Arg_ in 4T1‐bearing mice. (B) Tumor growth curves in 4T1‐bearing mice after different treatments. (C) Survival curves of 4T1‐bearing mice after different treatments. (D) Body weight changes of 4T1‐bearing mice after different treatments. (E) Intratumoral L‐arg levels after different treatments. (F–H) Flow cytometric analysis of the tumor infiltration of NK cells (F), Tregs (G), and MDSCs (H) in 4T1‐bearing mice after different treatments. (I‐K) Flow cytometric analysis of tumor infiltration of CD8^+^ (I), IFN‐γ^+^ CD8^+^ (J), and Ki‐67^+^ CD8^+^ (K) T cells after different treatments. (L) Flow cytometric analysis of DCs maturation (CD80^+^ CD86^+^ CD11c^+^) in tumor‐draining LNs after different treatments. (M) Schematic illustration for evaluating the antitumor effects of C‐EcN_Arg_ in B16F10‐bearing mice. (N) Tumor growth curves in B16F10‐bearing mice treated with different formations. (O) Survival curves of B16F10‐bearing mice after different treatments. (P) Body weight changes of B16F10‐bearing mice after different treatments. (Q) Intratumoral L‐arg levels after different treatments. (R‐T) Flow cytometric analysis of CD8^+^ (R), IFN‐γ^+^ CD8^+^ (S), and Ki‐67^+^ CD8^+^ (T) T cells after different treatments. Data in (B‐E) and (N–Q) are presented as means ± s.d. from six independent experiments (*n* = 6). Data in (F–I) and (R–T) are presented as means ± s.d. from four independent experiments (*n* = 4). Statistical analysis in (B), (D), (N), and (P) was performed using two‐way ANOVA followed by Sidak's test with multiple comparisons. Statistical analysis in (E–L) and (Q–T) was performed using one‐way ANOVA followed by Tukey's test with multiple comparisons. The significant levels are shown as ns > 0.05, *p < 0.05, **p < 0.01, ***p < 0.001, and ****p < 0.0001.

Benefiting from the excellent performance of surface polymer networks in reducing bacterial toxicity and preserving their therapeutic activity, we next investigated the synergistic antitumor efficacy of the combination of C‐EcN_Arg_ and αPD‐L1. The 4T1‐bearing mice were constructed via subcutaneous injection of 1 × 10^6^ 4T1 cells, αPD‐L1, EcN_Arg_+αPD‐L1, and C‐EcN_Arg_ were set as comparative groups (Figure 4A). After four times of treatment, αPD‐L1 and C‐EcN_Arg_ alone showed unsatisfactory tumor inhibitory effects, while EcN_Arg_+αPD‐L1 and C‐EcN_Arg_+αPD‐L1 showed higher tumor inhibitory effects (Figure 4B and Figure S13A), indicating the effective collaboration between L‐arg supplementation and PD‐1/PD‐L1 pathway blockade. As a result, EcN_Arg_+αPD‐L1 and C‐EcN_Arg_+αPD‐L1 significantly extended the median survival of the 4T1‐bearing tumor (Figure 4C). H&E and terminal deoxynucleotidyl transferase‐mediated deoxyuridine triphosphate nick end labeling (TUNEL) staining revealed extensive tumor cell apoptosis in mice treated with EcN_Arg_+αPD‐L1 and C‐EcN_Arg_+αPD‐L1 (Figure S13B), which is consistent with tumor progression observation. However, EcN_Arg_+αPD‐L1 treatment induced rapid weight loss (Figure 4D), indicating the severe toxicity of live bacterial medicines. In contrast, negligible weight loss was observed in mice treated with C‐EcN_Arg_ and C‐EcN_Arg_+αPD‐L1, demonstrating the effectiveness of surface polymer networks in improving bacterial biosafety.

To elucidate the therapeutic mechanism underlying the synergy of C‐EcN_Arg_ and αPD‐L1, tumors and lymph nodes (LNs) were collected after treatments. As expected, both C‐EcN_Arg_ and C‐EcN_Arg_+αPD‐L1 significantly elevated the intratumoral L‐arg concentrations (Figure 4E), demonstrating the preserved bioactivity of EcN_Arg_ post‐encapsulation. Next, we performed comprehensive flow cytometric analysis of dissociated tumors and LNs using standardized gating strategies (Figure S14). As shown in Figure 4F–H and Figure S15, L‐arg supplementation effectively alleviated the immunosuppressive TME, in which C‐EcN_Arg_+αPD‐L1 treatment induced higher levels of intratumoral natural killer cells (NK, CD49b^+^ CD3^−^) and M1‐like macrophages (CD86^+^ F4/80^+^ CD11b^+^) and lower levels of intratumoral M2‐like macrophages (CD206^+^ F4/80^+^ CD11b^+^), myeloid‐derived suppressor cells (MDSCs, Gr‐1^+^ CD11b^+^ CD45^+^), and regulatory T cells (Tregs, Foxp3^+^ CD4^+^ CD3^+^) compared to those of PBS treatment. Moreover, we found that L‐arg‐driven TME modulation further enhanced PD‐1/PD‐L1 blockade‐mediated antitumor T cell immune responses, validating by elevated proportions of CD8^+^, IFN‐γ^+^ CD8^+^, and Ki‐67^+^ CD8^+^ T lymphocytes (Figure 4I–K and Figure S16). Upregulation of intratumoral pro‐inflammatory factors (TNF‐α and IFN‐γ) and maturation of dendritic cells (DCs, CD80^+^ CD86^+^ CD11c^+^) in tumor‐draining LNs also confirmed the effective activation of systemic antitumor immune responses (Figure 4L and Figure S17). These results demonstrate that C‐EcN_Arg_ and αPD‐L1 exert potent synergistic antitumor effects through two coordinated mechanisms: (i) sustained L‐arg supplementation to alleviate immunosuppressive TME and (ii) effective immune checkpoint blockade to reactive antitumor T cell immunity.

In addition to the breast cancer model, we also investigated the effectiveness of the combination of C‐EcN_Arg_ and αPD‐L1 in a melanoma model. Melanoma‐bearing mice were constructed by subcutaneous injection of 1 × 10^6^ B16F10 cells and treated as described in Figure 4M. As shown in Figure 4N, O, and Figure S18, C‐EcN_Arg_ treatment significantly enhanced αPD‐L1‐mediated immune checkpoint blockade therapy, evidenced by effective tumor growth inhibition and extended survival time. Similarly, C‐EcN_Arg_ and C‐EcN_Arg_+αPD‐L1 treatments had negligible effect on mouse body weight, indicating its excellent biosafety (Figure 4P). Thereafter, we analyzed the tumor immune microenvironment. Accompanied by elevated L‐arg concentrations (Figure 4Q), C‐EcN_Arg_+αPD‐L1 effectively alleviated the immunosuppressive TME, including an increase in the proportions of NK cells (NK1.1^+^ CD3^−^) and M1‐like macrophages, and a decrease in the proportions of M2‐like macrophages, MDSCs, and Tregs (Figure S19). Furthermore, C‐EcN_Arg_+αPD‐L1 achieved robust activation of T cell‐mediated antitumor immunity, as demonstrated by increased levels of tumor‐infiltrating CD8^+^, IFN‐γ^+^ CD8^+^, and Ki‐67^+^ CD8^+^ T lymphocytes and effective DCs maturation in tumor‐draining LNs (Figure 4R–T and Figure S20), confirming the potential of C‐EcN_Arg_ in enhancing immune checkpoint blockade therapy and activating systemic antitumor immune responses.

### 2.4 Construction of EcN_Arg+sPD‐1_ and Biodegradable Bacterial Capsule

**FIGURE 5:**
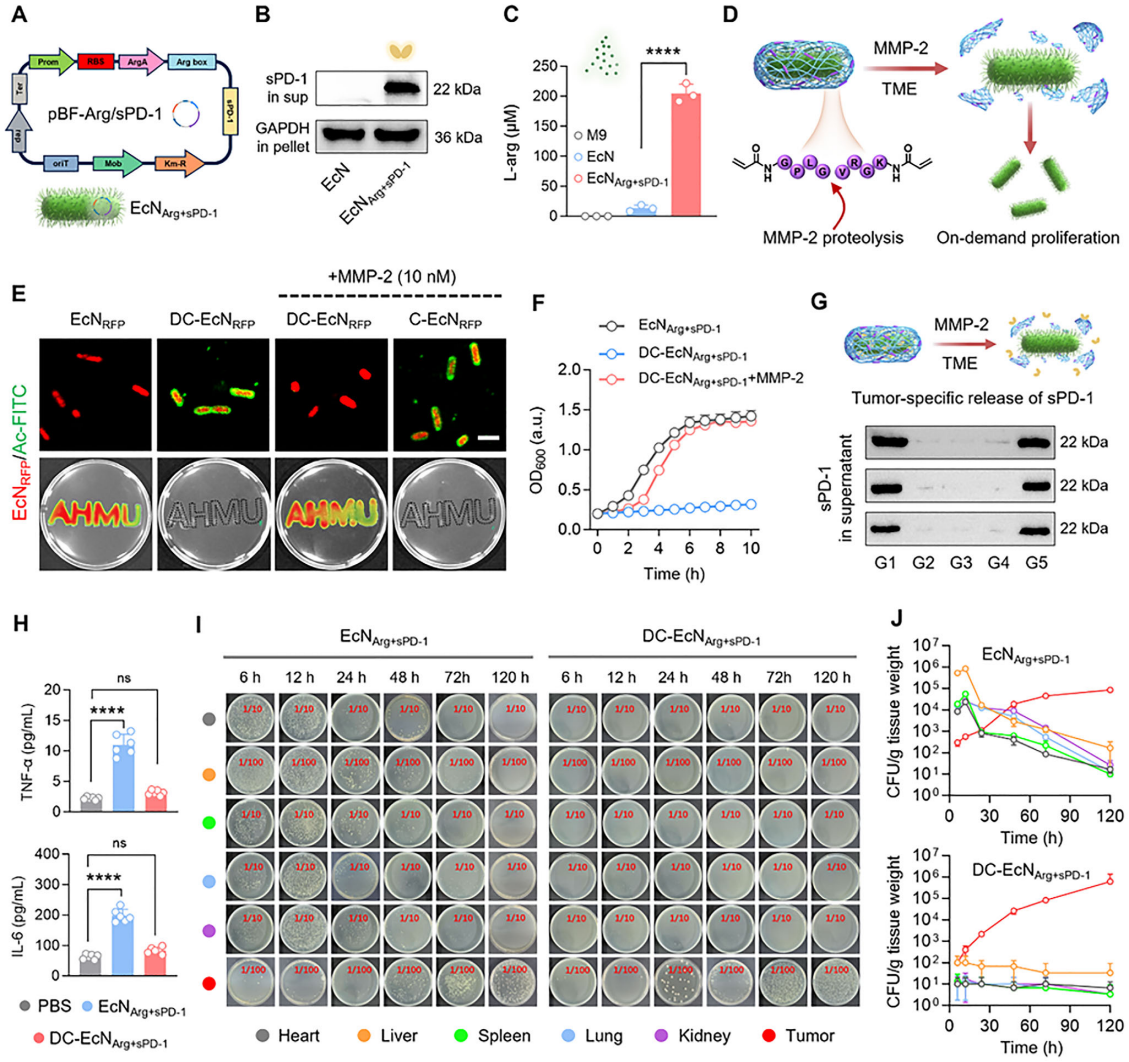
Preparation and characterization of biodegradable bacterial capsule. (A) Plasmid DNA map of EcN_Arg+sPD‐1_. (B) Western blot assay of sPD‐1 in the supernatant of EcN_Arg+sPD‐1_. (C) Non‐engineered EcN and EcN_Arg+sPD‐1_ were cultured in a medium containing 5 mm NH_4_Cl as the only nitrogen source and then incubated at 37°C for 4 h. L‐arg levels in the supernatant of the medium were measured using a L‐arg detection kit. (D) Schematic illustration of DC‐EcN_Arg+sPD‐1_ and on‐demand proliferation in response to endogenous MMP‐2. (E) Upper layer: CLSM images of EcN_RFP_, C‐EcN_RFP,_ and DC‐EcN_RFP_ in the presence or absence of MMP‐2 (10 nm). Scale bar: 1 µm. Lower layer: Fluorescence photographs of LB agar plates spread with EcN_RFP_, C‐EcN_RFP,_ and DC‐EcN_RFP_ in the presence or absence of MMP‐2 (10 nm). (F) Time‐dependent growth curves of EcN_Arg+sPD‐1_ and DC‐EcN_Arg+sPD‐1_ in the presence or absence of MMP‐2 (10 nm) at 37°C. (G) WB analysis of sPD‐1 levels in the supernatant of C‐EcN_Arg+sPD‐1_ and DC‐EcN_Arg+sPD‐1_ in the absence or presence of MMP‐2 (10 nm). G1: EcN_Arg+sPD‐1_, G2: C‐EcN_Arg+sPD‐1_, G3: C‐EcN_Arg+sPD‐1_+MMP‐2, G4: DC‐EcN_Arg+sPD‐1_, G5: DC‐EcN_Arg+sPD‐1_+MMP‐2. (H) TNF‐α and IL‐6 levels in mouse serum after intravenous injection of PBS, EcN_Arg+sPD‐1_, and DC‐EcN_Arg+sPD‐1_, respectively. (I) Representative photographs of LB agar plates spread with homogenate of normal organs and tumors at different time points after systemic administration of EcN_Arg+sPD‐1_ and DC‐EcN_Arg+sPD‐1_. (J) Bacterial count analysis in normal organs and tumor tissues. Data in (C), (F), and (J) are presented as means ± s.d. from three independent experiments (*n* = 3). Data in (H) are presented as means ± s.d. from six independent experiments (*n* = 6). Statistical analysis in (C) and (H) was performed using one‐way ANOVA followed by Tukey's test with multiple comparisons. The significant levels are shown as ns > 0.05 and ****p < 0.0001.

As an emerging therapeutic modality, living bacterial medicines exhibit unique advantages in drug delivery and in situ production, also drawing extensive attention in protein therapeutics. Engineered bacteria can colonize at lesion sites and sustainable secrete therapeutic antibodies, addressing the short half‐life of conventional antibodies, reducing risks of systemic immune responses, and lowering clinical costs. To leverage these benefits, we constructed an EcN_Arg+sPD‐1_ to co‐express L‐arg and sPD‐1 for synergistic immunotherapy. (Figure 5A and Figure S1B). Western blot and ELISA assays confirmed the successful expression and secretion of sPD‐1 by EcN_Arg+sPD‐1_ (Figure 5B and Figure S21A). The potential of EcN_Arg+sPD‐1_ in converting intratumoral metabolized ammonium into therapeutic L‐arg was also demonstrated (Figure 5C). A prominent advantage of living bacterial medicines over conventional drugs lies in their proliferation at lesion sites, enabling in situ therapeutic amplification. To achieve on‐demand bacterial proliferation within the tumors, a cleavable peptide cross‐linker (Ac‐GPLGVRGK‐Ac, detailed characterization is shown in Figure S22A) that can be cleaved by MMP‐2 was synthesized [53, 54]. The successful preparation of a degradable EcN_Arg+sPD‐1_ capsule (DC‐EcN_Arg+sPD‐1_) was confirmed by TEM images and DLS measurements (Figures S22B–D). Unlike a non‐degradable bacterial capsule, DC‐EcN_Arg+sPD‐1_ enables tumor‐selective bacterial release and subsequent localized proliferation (Figure 5D). To demonstrate this, we first assessed MMP‐2‐responsive degradation of bacterial capsule. As shown in Figure 5E, polymer networks (green) on DC‐EcN_RFP_ surface disappeared after incubating with MMP‐2 (10 nm) for 2 h, whereas non‐degradable C‐EcN_RFP_ remained intact. We also evaluated MMP‐2 responsive degradation of bacterial capsules at different enzyme concentrations usingAc‐FITC. As shown in Figure S23, the cumulative shedding rates of surface polymer without MMP‐2 remained below 5% over 24 h, while the cumulative shedding rates under 10 nm MMP‐2 incubation rapidly increased to 88.35% within 8 h. We next evaluated bacterial proliferation after MMP‐2 treatment. Plate colony assay and bacterial growth monitoring suggested that surface polymer networks initially inhibit bacterial proliferation (Figure 5F and Figure S24), which is consistent with prior results. However, DC‐EcN_Arg+sPD‐1_ recovered proliferative activity after MMP‐2 treatment, while non‐degradable C‐EcN_Arg+sPD‐1_ showed negligible degradation of surface polymer and continuous proliferation inhibition after MMP‐2 treatment. These results further demonstrate that the proliferation inhibition post‐polymerization is governed by physical confinement caused by the high‐density polymer networks, and DC‐EcN_Arg+sPD‐1_ made with degradable cross‐linkers enables selective bacterial release and localized proliferation in response to intratumoral MMP‐2.

As described previously, cross‐linked polymer network on the bacterial surface confers effective protection against lysozyme‐mediated bacterial lysis through physical shielding. We therefore investigated whether the surface polymer network can prevent non‐specific release of therapeutic proteins secreted by bacteria. To avoid the impact of bacterial proliferation, bacterial densities were uniformly diluted to 5 × 10^7^ CFU mL^−1^ after MMP‐2 (10 nm) treatment. The supernatants were then collected by centrifugation. As shown in Figure 5G and Figure S21B, little sPD‐1 was detected in the supernatants of either C‐EcN_Arg+sPD‐1_ or DC‐EcN_Arg+sPD‐1_, while MMP‐2 treatment recovered sPD‐1 secretion in the supernatant of DC‐EcN_Arg+sPD‐1_. In contrast, MMP‐2 failed to recover sPD‐1 secretion from non‐degradable C‐EcN_Arg+sPD‐1_, suggesting that a degradable polymer network on the bacterial surface enables specific release of therapeutic proteins in response to intratumoral MMP‐2. By preventing the leakage of sPD‐1, DC‐EcN_Arg+sPD‐1_ may minimize undesirable immune activation.

To evaluate the biosafety of DC‐EcN_Arg+sPD‐1_, we first analyzed inflammatory factor levels in serum following systemic administration. Compared to unencapsulated EcN_Arg+sPD‐1_, much lower levels of TNF‐α and IL‐6 were detected in serum of DC‐EcN_Arg+sPD‐1_‐treated mice (Figure 5H), demonstrating excellent biocompatibility of DC‐EcN_Arg+sPD‐1_. Furthermore, we conducted comprehensive analysis on blood and major organs, including histopathological examination, blood biochemical, and routine blood assays. As shown in Figure S25, EcN_Arg+sPD‐1_ induced obvious inflammation and bacterial infection, whereas DC‐EcN_Arg+sPD‐1_ showed little alteration in these infection indicators (WBC, PLT, and NEUT), as well as many hepatic function (ALT, AST, GGT, and LDH) and renal function indicators (UREA, CREA). Ultimately, we evaluated bacterial tumor colonization following systemic administration. Similarly to C‐EcN_Arg_, EcN_Arg+sPD‐1_ encapsulated in DC‐EcN_Arg+sPD‐1_ showed no proliferation in healthy organs (Figure 5I, J). In contrast, colony counting assays showed that systemically administrated DC‐EcN_Arg+sPD‐1_ recovered bacterial proliferation in MMP‐2‐enriched tumors. To evaluate the long‐term biosafety of bacterial capsules, C‐EcN_Arg+sPD‐1_ and DC‐EcN_Arg+sPD‐1_ were intravenously injected into healthy mice. At 30 days post‐treatment, major organs and blood were collected for histopathological, blood biochemical, and routine blood analysis. The results showed that no obvious change was observed from these parameters after C‐EcN_Arg+sPD‐1_ and DC‐EcN_Arg+sPD‐1_ treatments, suggesting the good biosafety of the bacterial capsule (Figure S26A, B). Plate colony analysis of major organs showed that all bacteria were eventually completely cleared (Figure S26C). Collectively, the degradable bacterial capsule developed in this work may simultaneously achieve immunogenic surface shielding, low off‐target leakage of protein payloads, and on‐demand bacterial proliferation, providing a feasible encapsulation platform to overcome the safety risks and delivery challenges of bacterial medicines.

### 2.5 Enhanced Cancer Immunotherapy With DC‐EcN_Arg+sPD‐1_

**FIGURE 6:**
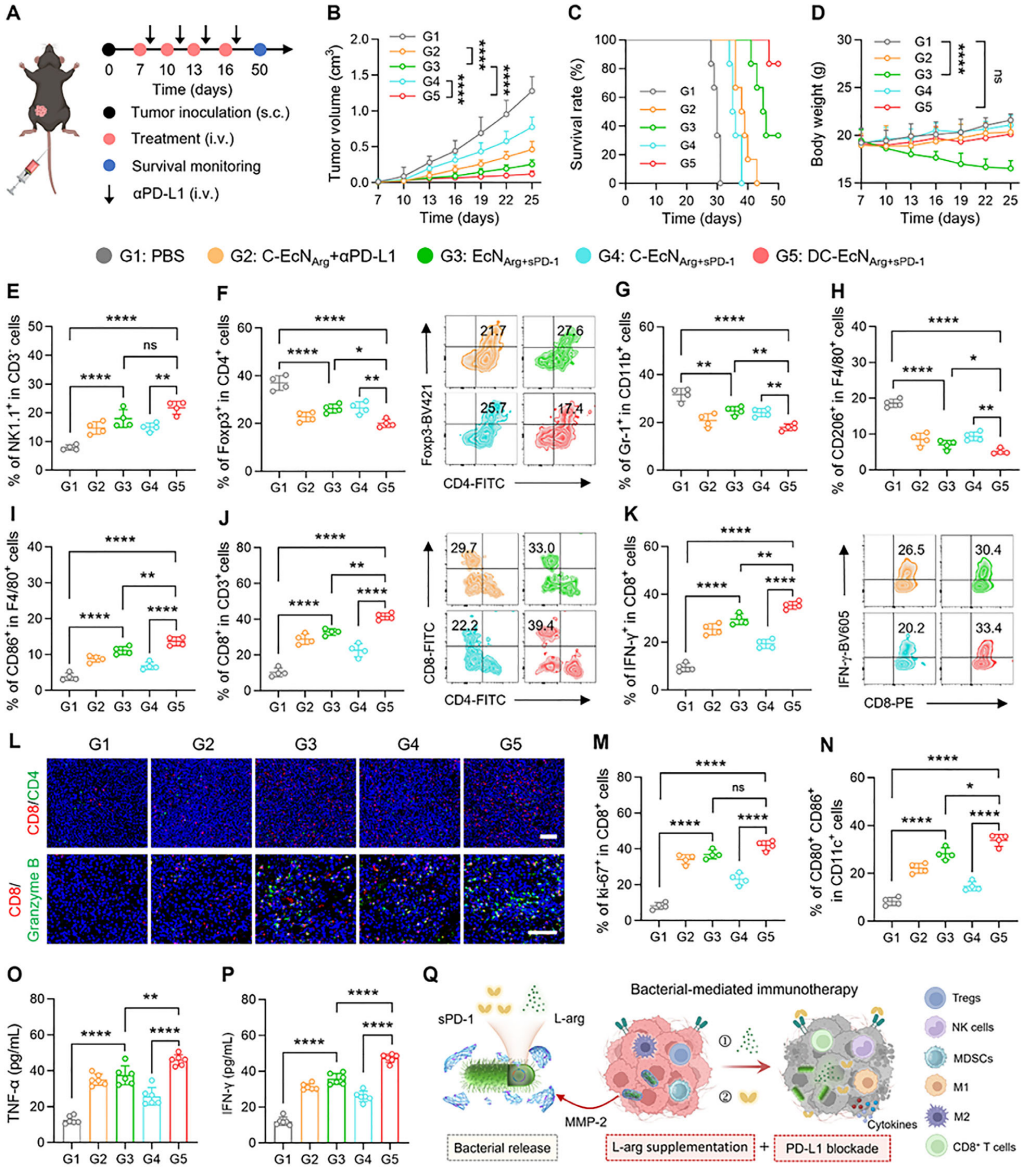
Enhanced cancer immunotherapy with DC‐EcN_Arg+sPD‐1_. (A) Schematic illustration for evaluating the antitumor effect of DC‐EcN_Arg+sPD‐1_ in B16F10‐bearing mice. (B) Tumor growth curves in B16F10‐bearing mice after different treatments. (C) Survival curves of B16F10‐bearing mice after different treatments. (D) Body weight changes of B16F10‐bearing mice after different treatments. (E–I) Flow cytometric analysis of the tumor infiltration of NK cells (E), Tregs (F), MDSCs (G), M2‐like macrophages (H), and M1‐like macrophages (I) in B16F10‐bearing mice after different treatments. (J and K) Flow cytometric analysis of tumor‐infiltrating CD8^+^ (J) and IFN‐γ^+^ CD8^+^ (K) T cells after different treatments. (L) Immunofluorescence analysis of CD8^+^ and Granzyme^+^ CD8^+^ T cells in tumor tissues after different treatments; Scale bars: 200 µm. (M) Flow cytometric analysis of tumor‐infiltrating Ki‐67^+^ CD8^+^ T cells after different treatments. (N) Flow cytometric analysis of DCs maturation in tumor‐draining LNs after different treatments. (O and P) Intratumoral TNF‐α (O) and IFN‐γ (P) levels after different treatments. (Q) Schematic diagram of therapeutic mechanism of DC‐EcN_Arg+sPD‐1_‐mediated immunotherapy. Data in (B–D) and (O–P) are presented as means ± s.d. from six independent experiments (*n* = 6). Data in (E–K) and (M, N) are presented as means ± s.d. from four independent experiments (*n* = 4). Statistical analysis in (B) and (D) was performed using two‐way ANOVA followed by Sidak's test with multiple comparisons. Statistical analysis in (E–K) and (M–P) was performed using one‐way ANOVA followed by Tukey's test with multiple comparisons. The significant levels are shown as ns > 0.05, *p < 0.05, **p < 0.01, ***p < 0.001, and ****p < 0.0001.

Next, we evaluated the antitumor effects of DC‐EcN_Arg+sPD‐1_, B16F10‐bearing model was constructed as described previously and treated with DC‐EcN_Arg+sPD‐1_ as indicated in Figure 6A. C‐EcN_Arg_+αPD‐L1, EcN_Arg+sPD‐1_, and C‐EcN_Arg+sPD‐1_ were set as comparative groups. As shown in Figure 6B and Figure S27, although C‐EcN_Arg_+αPD‐L1 markedly inhibited tumor growth and induced extensive tumor cell apoptosis, DC‐EcN_Arg+sPD‐1_ displayed superior antitumor efficacy, which is mainly due to local and sustainable production of sPD‐1 for PD‐1/PD‐L1 pathway blockade [55, 56]. Similarly, DC‐EcN_Arg+sPD‐1_ significantly prolonged the survival time of B16F10‐bearing mice, and five out of six mice remained alive at day 50 post‐tumor inoculation (Figure 6C). In contrast, DC‐EcN exhibited negligible tumor inhibition, verifying that the wild‐type EcN and surface polymer made with MPC have no significant antitumor activity (Figure S28). Non‐degradable C‐EcN_Arg+sPD‐1_ showed lower antitumor efficacy, which may be due to limited sPD‐1 release and bacterial proliferation. Benefiting from optimized in vivo pharmacokinetics and low MPS clearance, DC‐EcN_Arg+sPD‐1_ displayed a higher efficacy than direct intravenous injection of EcN_Arg+sPD‐1_ and the physical mixture of EcN_Arg_ and EcN_sPD‐1_. Furthermore, compared to EcN_Arg+sPD‐1_ treatment, no obvious weight loss was detected in the mice treated with DC‐EcN_Arg+sPD‐1_, demonstrating the potential of surface polymerization in attenuating bacterial toxicity (Figure 6D). To elucidate the therapeutic mechanism of DC‐EcN_Arg+sPD‐1_, intratumoral levels of sPD‐1 and L‐arg were first measured. As shown in Figure S29, obvious sPD‐1 expression and a higher L‐arg level (6.45‐fold over that of PBS) were detected in tumors of the mice treated with DC‐EcN_Arg+sPD‐1_ (All unprocessed western blot images were illustrated in Figure S30). In contrast, little intratumoral sPD‐1 was detected in mice treated with a non‐degradable EcN_Arg+sPD‐1_ capsule (C‐EcN_Arg+sPD‐1_). Therefore, the antitumor efficacy of C‐EcN_Arg+sPD‐1_ is solely attributable to L‐arg‐driven TME modulation, which aligns with the observed antitumor outcomes.

To demonstrate the potential of DC‐EcN_Arg+sPD‐1_ in activating antitumor immune responses, tumors and LNs were collected and homogenized for flow cytometric analysis after treatments. As described previously, DC‐EcN_Arg+sPD‐1_ effectively alleviated the immunosuppressive TME, as evidenced by a pronounced decrease in M2‐like macrophages, Tregs, and MDSCs, alongside a significant increase in NK cells and M1‐like macrophages (Figure 6E–I and Figure S31). Leveraging localized sPD‐1 production and reprogrammed TME, DC‐EcN_Arg+sPD‐1_ elicited the robust tumor‐infiltration of CD8^+^ T cells, including IFN‐γ^+^ and Ki‐67^+^ subsets (Figure 6J, K, M, and Figure S32), indicating enhanced cytotoxic and proliferative activity. Immunofluorescence analysis confirmed the efficient tumor infiltration (CD8^+^) and enhanced cytotoxic activity (Granzyme^+^ CD8^+^) of T lymphocytes (Figure 6L). Accordingly, the highest levels of TNF‐α (Figure 6O) and IFN‐γ (Figure 6P) were detected in tumor tissues after DC‐EcN_Arg+sPD‐1_ treatment, validating the effective activation of T cell‐mediated antitumor immune responses. Furthermore, DC‐EcN_Arg+sPD‐1_ significantly promoted DC maturation in LNs, suggesting the activation of systemic antitumor immunity (Figure 6N and Figure S33). These results demonstrate that DC‐EcN_Arg+sPD‐1_ can effectively activate the body's antitumor immune responses by synergistically alleviating immunosuppressive TME and blocking the PD‐1/PD‐L1 pathway, and the therapeutic mechanism is illustrated in Figure 6Q.

**FIGURE 7:**
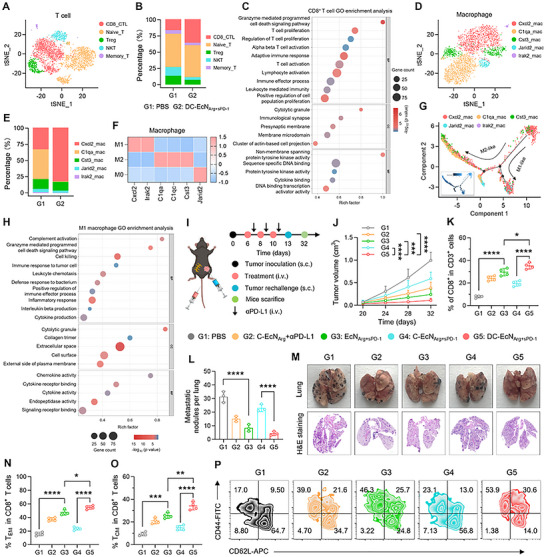
Single‐cell RNA sequencing analysis and inhibitory effects of DC‐EcN_Arg+sPD‐1_ on tumor recurrence and metastasis. (A) The t‐SNE plot for subgrouping analysis of T cells in the tumors. (B) The proportion of T cell subtypes in PBS and DC‐EcN_Arg+sPD‐1_ groups. (C) GO enrichment analysis of CD8^+^ T cells in DC‐EcN_Arg+sPD‐1_‐treated tumors. (D) The t‐SNE plot for subgrouping analysis of macrophage cells in the tumors. (E) The proportion of macrophage subtypes in PBS and DC‐EcN_Arg+sPD‐1_ groups. (F) M0, M1, and M2 signature scores based on the gene signatures from CIBERSORT. (G) The developmental trajectory analysis of macrophages. (H) GO enrichment analysis of M1 macrophages in DC‐EcN_Arg+sPD‐1_‐treated tumors. (I) Schematic illustration for evaluating the potential of DC‐EcN_Arg+sPD‐1_ in preventing tumor recurrence. (J) Rechallenged tumor growth curves after different treatments. (K) Flow cytometric analysis of CD8^+^ T cells in rechallenged tumors after different treatments. (L) Quantitative analysis of metastatic nodules on the lung surface after different treatments. (M) Lung images and H&E staining analysis after different treatments. (N‐P) Flow cytometric quantitative analysis of T_EM_ (N), T_CM_ (O), and represented plots (P) in spleens after different treatments. Data in (J) are presented as means ± s.d. from six independent experiments (*n* = 6). Data in (K), (N), and (O) are presented as means ± s.d. from four independent experiments (*n* = 4). Data in (L) are presented as means ± s.d. from three independent experiments (*n* = 3). Statistical analysis in (J) was performed using two‐way ANOVA followed by Sidak's test with multiple comparisons. Statistical analysis in (K), (L), (N), and (O) was performed using one‐way ANOVA followed by Tukey's test with multiple comparisons. The significant levels are shown as *p < 0.05, **p < 0.01, ***p < 0.001, and ****p < 0.0001.

To further explore the potential of DC‐EcN_Arg+sPD‐1_ in activating antitumor immune responses, we conducted single‐cell sequencing (scRNA‐seq) analysis on CD45‐gated cells in tumors after PBS and DC‐EcN_Arg+sPD‐1_ treatments on day 17. The scRNA‐seq data were processed using the Seurat package, and cell clusters were identified using unsupervised clustering analysis. All CD45‐positive cells were identified as eight distinct cell clusters, including macrophage, T cell, neutrophil, B cell, plasma, DC, monocyte, and mast cell (Figure S34A,C). Next, T cells were clustered into five subclusters (Figure 7A). As shown in Figure 7B and Figure S34D, DC‐EcN_Arg+sPD‐1_ treatment significantly elevated the proportion of effector CD8^+^ T (*Bcl11b*, *Satb1*, and *Tnf*) cells, indicating the effective activation of T cell immune responses. In addition, DC‐EcN_Arg+sPD‐1_ treatment remarkably reduced the population of Tregs (*Foxp3*, *Ctla4*, and *Ikzf2*), which serve as a critical suppressive role to restrict the efficacy of immune checkpoint blockade therapy. To further evaluate the biological processes modulated by DC‐EcN_Arg+sPD‐1_, Gene Ontology (GO) enrichment analysis was performed on the differentially expressed genes (DEGs) of CD8^+^ T cells in DC‐EcN_Arg+sPD‐1_‐treated tumors (Figure 7C). The result revealed significant enrichment in multiple biological processes, including granzyme‐mediated programmed cell death signaling pathway, T cell proliferation, T cell activation, adaptive immune response, and immune effector process, validating the effective activation of T cell‐mediated antitumor immune responses.

We next analyzed the macrophages with the maximum proportion in CD45‐positive cells. Macrophages can be subdivided into five functionally distinct subclusters, and showed diverse subcluster distributions among PBS and DC‐EcN_Arg+sPD‐1_ groups (Figure 7D). C1qa_macro and Cst3_macro were predominantly found in PBS‐treated tumors, whereas Cxcl2_macro was abundant in DC‐EcN_Arg+sPD‐1_‐treated tumors (Figure 7E). Cxcl2_macro can enhance inflammatory responses and facilitate the antigen presentation process. In contrast, C1qa_macro and Cst3_macro exhibited characteristics of tumor angiogenesis, immunotherapy resistance, and promotion of tumor invasion and metastasis [57]. Macrophages are usually classified into three subtypes: non‐polarized M0 (neutral), pro‐inflammatory M1 (anti‐tumor), and anti‐inflammatory M2 (pro‐tumor) [58]. Module scoring analysis demonstrated M1 increase (Cxcl2_macro and Irak2_macro) and M2 reduction (C1qa_macro and Cst3_macro) after DC‐EcN_Arg+sPD‐1_ treatment (Figure 7E, F, and Figure S34E), which is consistent with the flow cytometry experiment. Trajectory analysis was conducted to investigate the impact of DC‐EcN_Arg+sPD‐1_ on different macrophage subtypes. These macrophages started in non‐polarized M0 and then differentiated into pro‐inflammatory M1 and anti‐inflammatory M2 (Figure 7G). The trajectory heat map showed sequential gene expression changes and the gradual switch from M2 to M1 (Figure S34F). In addition, GO enrichment analysis of M1 macrophages revealed significant enrichment in many biological processes and molecular functions, such as immune response to tumor cells, positive regulation of immune effector process, inflammatory response, cytokine production, cytokine activity, cytokine receptor binding, and chemokine activity (Figure 7H). Overall, these results indicate that DC‐EcN_Arg+sPD‐1_ can promote the polarization of tumor‐associated macrophages toward a pro‐inflammatory M1 phenotype with enhanced antitumor activity while reducing protumoral M2‐like macrophages, thus alleviating immunosuppressive TME.

A defining advantage of cancer immunotherapy lies in its capacity to generate durable protective immunity, conferring long‐term surveillance against tumor recurrence and distant metastasis. To evaluate this capacity in our system, we conducted sequential tumor rechallenge experiments. In the tumor rechallenge model (Figure 7I), the mice bearing primary B16F10 tumors on the left flank received various treatments on day 6, 8, and 10 post‐inoculation. Subsequent subcutaneous rechallenge with B16F10 cells on the contralateral flank (day 13) revealed that DC‐EcN_Arg+sPD‐1_ treatment significantly delayed the growth of rechallenged tumors (Figure 7J and Figure S35A), along with complete tumor rejection observed in two of six mice. Accordingly, much higher levels of CD8^+^ T cells were detected in rechallenged tumors of mice treated with DC‐EcN_Arg+sPD‐1_, indicating the efficient activation of cognate antitumor immunity (Figure 7K and Figure S35B). We next evaluated the antimetastatic potential of DC‐EcN_Arg+sPD‐1_ by intravenous challenge with B16F10 cells at day 13 post‐inoculation. At the end of treatment, lungs were collected, and lung metastatic nodules were counted. Compared to other treatments, much less metastatic nodules on the lung surface were observed in mice treated with DC‐EcN_Arg+sPD‐1_, and histopathological evaluation confirmed the antimetastatic effect (Figure 7L, M). In addition, flow cytometric analysis revealed that DC‐EcN_Arg+sPD‐1_ significantly elevated the proportions of effector memory (T_EM_, CD3^+^ CD8^+^ CD44^+^ CD62L^−^) and central memory T cell populations (T_CM_, CD3^+^ CD8^+^ CD44^+^ CD62L^+^) in spleens compared to other treatments (Figure 7N–P and Figure S35C). Together, these results suggest that DC‐EcN_Arg+sPD‐1_ can effectively prevent tumor recurrence and distant metastasis by activating long‐term immune memory.

## 3 Conclusion

Bacteria have attracted extensive attention and exploration for their advantages of targeted colonization in pathological tissues and local production of therapeutic payloads. Despite these benefits, clinical translation of bacterial medicines faces serious challenges due to host toxicity, restricting their tolerable doses and compromising therapeutic outcomes. In this work, we presented a platform technology to improve therapeutic bacterial biosafety and delivery efficacy by mitigating bacterial immunogenicity and controlling its proliferation. This bacterial encapsulation platform was achieved using an in situ free‐radical polymerization method, which can formulate individual bacteria with a thin formulation layer of cross‐linked polymers, enabling immunogenic surface shielding, prolonged blood circulation, and bacterial proliferation control, addressing biosafety and delivery challenges after systemic administration. Additionally, to achieve on‐demand proliferation at tumor tissues, degradable bacterial capsule was synthesized using an enzyme‐responsive cross‐linker that could cleave by TME‐specific MMP‐2, enabling TME‐responsive bacterial release and localized proliferation. For cancer immunotherapy, an *E. coli* Nissle strain capable of simultaneously converting tumor‐accumulated ammonia into L‐arg and secreting sPD‐1 was constructed and encapsulated to form a degradable bacterial capsule, optimizing biosafety and the systemic delivery process. Upon reaching the tumors, MMP‐2 triggers bacterial release and proliferation, enabling L‐arg‐driven tumor microenvironment modulation and sustained PD‐L1 blockade, potently activating antitumor immune responses. Overall, this work proposed a universal encapsulation technology to address safety risks and delivery challenges of live bacteria, providing insights for advancing the clinical application of live bacterial therapeutics.

Despite many promising results of DC‐EcN_Arg+sPD‐1_, several challenges and opportunities for improvement remain to be addressed in future studies. First, attenuated EcN was employed as a proof‐of‐concept model in this work. To assess the broad applicability of our strategy, strains (e.g., *Salmonella*, *Listeria*, and *Clostridium*) with high toxicity and different surface structures should be encapsulated and conducted comprehensive safety evaluation. Second, various monomers and cross‐linkers for bacterial encapsulation are commercially available and readily customizable. This modular encapsulation method allows precise tuning of bacterial capsule properties to further optimize both tumor‐selective proliferation and biosafety profiles. In addition, this encapsulation strategy is anticipated to enhance biosafety and effectiveness of live bacterial therapeutics in multiple human diseases by dynamically responding to specific pathological microenvironments. Third, advances in synthetic biology and genetic engineering have enabled precise spatiotemporal control of therapeutic payload production in response to endogenous (e.g., pH and hypoxia) or exogenous stimuli (e.g., light, thermal, and ultrasound). Integration of stimulus‐responsive genetic circuits with surface polymerization could establish a dual‐layered safety mechanism, simultaneously reducing bacterial toxicity while augmenting their therapeutic efficacy. Fourth, this bacterial encapsulation strategy is highly compatible, holding great promise in overcoming the host toxicity and delivery challenges of multiple microbes (e.g., microalgae, bacteriophages, and fungi) through in situ formation of customized polymer networks on their surface.

## 4 Experimental Section

### 4.1 Bacteria Strains and Functional Plasmids

Attenuated *Escherichia coli* Nissle 1917 (EcN) and EcN_RFP_ used in this study were obtained from MiaoLing Technology (Wuhan, China). To construct EcN_Arg_ that can convert tumor‐accumulated ammonia, a byproduct of glutamine metabolism, to therapeutically active L‐arg, pBF‐Arg‐M215 plasmid was constructed by inserting the encoding sequence of ArgA and Arg box into the pBF‐pTrc‐B0035 plasmid using the multiple cloning site (Table S1). Thereafter, pBF‐Arg‐M215 plasmid was transformed into EcN through a heat shock transformation method, yielding engineered EcN_Arg_. To construct EcN_Arg_ that can simultaneously produce L‐arg and secrete sPD‐1, pBF‐Arg+sPD‐1 plasmid was constructed by encoding the extracellular domain protein of the mouse PD‐1 sequence (amino acids 1 to 169, UniProt accession number Q02242) with a pelB signal peptide sequence at the N‐terminus into the pBF‐Arg‐M215 plasmid using the multiple cloning site (Table S1). Next, pBF‐Arg+sPD‐1 plasmid was transformed into EcN through a heat shock transformation method, yielding engineered EcN_Arg+sPD‐1_. All plasmids were designed using SnapGene (GSL Biotech) and assembled using Gibson Assembly reagents from New England Biolabs (E2621L). Additional genes and all PCR primers were synthesized by Sangon Biotech.

### 4.2 Bacterial Culture for In Vitro and In Vivo Experiments

All bacteria were cultured in LB broth and grown on LB agar plates supplemented with appropriate antibiotics. Single colonies were picked and inoculated into LB broth, followed by overnight incubation in a shaking incubator (37°C, 200 rpm). For antibiotic selection, kanamycin (Beyotime Biotech, Shanghai, China) was added to the culture medium at a final concentration of 10 µg mL^−1^. Next day, the optical density at 600 nm (OD_600_) was measured, and saturated cultures were diluted to an OD_600_ of 0.2. Diluted cultures were grown to the exponential phase (OD_600_ = 1.5) prior to assays. OD_600_ measurements were performed in triplicate using a UV1900 spectrophotometer (SHIMADZU, Japan) in cuvette mode to ensure reproducibility.

### 4.3 Cell Culture

All cell lines were procured from the American Type Culture Collection (ATCC). 4T1 murine breast cancer cells were maintained in Dulbecco's Modified Eagle Medium (DMEM) supplemented with 10% (v/v) fetal bovine serum (FBS). B16F10 murine melanoma cells were cultured in Roswell Park Memorial Institute (RPMI)‐1640 medium containing 10% (v/v) FBS, 100 U mL^−1^ penicillin, and 100 µg mL^−1^ streptomycin (Biochannel). Cultures were maintained at 37°C in a humidified 5% CO_2_ incubator, with medium changes every 2–3 days and passaging at 80% confluence using 0.25% trypsin‐EDTA (Biochannel).

### 4.4 Preparation and Characterization of C‐EcN_Arg_

To synthesize C‐EcN_Arg_, 1 mL EcN_Arg_ suspension (1 × 10^8^ CFU mL^−1^) was centrifuged (3500 g, 5 min, 4°C) in a 1.5 mL sterile microcentrifuge tube, and the supernatant was discarded. The pellet was resuspended in 888 µL of fresh sterile ice‐cold PBS. The following monomer solutions were then added sequentially: 100 µL MPC (50 mg mL^−1^ in PBS), and 10 µL BIS (50 mg mL^−1^ in DMSO). After incubation for 10 min at 4°C, polymerization was initiated by adding 1 µL APS (100 mg mL^−1^ in water) and 1 µL TEMED (775 mg mL^−1^ in water). The mixture was maintained at 4–8°C and shaking (200 rpm) for 2 h. Encapsulated bacteria (C‐EcN_Arg_) were collected by centrifugation, washed with sterile ice‐cold PBS, and stored at 4°C for subsequent use.

Dynamic light scattering (DLS) was used to measure the size distribution and zeta potential of EcN_Arg_ and C‐EcN_Arg_ using a Zetasizer Nano ZS (Malvern Instruments), with each sample analyzed in triplicate and averaged. For transmission electron microscopy (TEM) observation, EcN_Arg_ and C‐EcN_Arg_ were prepared as described, diluted in deionized water to 1 × 10^7^ CFU mL^−1^, and 10 µL sample solutions were dropped onto a copper grid, allowed to stand for 10 min, and excess liquid was blotted off with filter paper. This process was repeated, followed by staining with 10 µL 2% phosphotungstic acid for 5 s. Excess liquid was removed with filter paper, and grids were vacuum‐dried for 24 h before imaging on a JEM‐2100 TEM (JEOL) at 200 kV.

To demonstrate the successful polymerization on a single bacterial surface, a fluorescent‐labeled monomer (Ac‐FITC) was synthesized. Briefly, FITC (778 mg, 2.0 mmol) was dissolved in dichloromethane (20 mL), APm (426 mg, 3.0 mmol) was added under ice bath conditions and stirred for 2 h. The solvent was then removed through reduced‐pressure concentration, and the crude product was purified using a C18 reverse‐phase column (MeOH/DCM = 1/15) to obtain yellow solid Ac‐FITC (yield, 968.5 mg, 91%). ^1^H NMR (400 MHz, DMSO‐*d*_6_) δ 10.13 (s, 3H), 8.23 (d, *J* = 2.0 Hz, 1H), 8.17 (s, 1H), 8.00 (t, *J* = 5.8 Hz, 1H), 7.75 (d, *J* = 8.3 Hz, 1H), 7.19 (d, *J* = 8.3 Hz, 1H), 6.68 (d, *J* = 2.3 Hz, 2H), 6.64‐6.54 (m, 4H), 5.67 (t, *J* = 1.3 Hz, 1H), 5.35‐5.31 (m, 1H), 3.60‐3.49 (m, 2H), 3.22‐3.16 (m, 2H), 1.90‐1.82 (m, 3H), 1.74 (p, *J* = 6.9 Hz, 2H). ESI‐MS: [M+H]: m/z: calcd. (C_28_H_25_N_3_O_6_S): 532.15, found: 532.10.

To directly observe the polymer network on the bacterial surface, Ac‐FITC and RFP‐labelled EcN_RFP_ were employed to synthesize C‐EcN_RFP_. Briefly, 1 mL EcN_RFP_ suspension (1 × 10^8^ CFU) was centrifuged (3500 g, 5 min, 4°C) in a 1.5 mL sterile microcentrifuge tube, and the supernatant was discarded. The pellet was resuspended in 888 µL of fresh ice‐cold PBS. The following monomer solutions were then added sequentially: 80 µL MPC (50 mg mL^−1^ in PBS), 20 µL Ac‐FITC (100 mg mL^−1^ in PBS), and 10 µL BIS (50 mg mL^−1^ in DMSO). After incubation for 10 min, polymerization was initiated by adding 1 µL APS (100 mg mL^−1^ in water) and 1 µL TEMED (775 mg mL^−1^ in water). The mixture was maintained at 4–8°C and shaking for 4 h. Unreacted monomers were removed by centrifugation, C‐EcN_RFP_ was observed using a confocal laser scanning microscope (ZEISS, Germany), RFP: 561 nm excitation, 580–620 nm emission; FITC: 488 nm excitation, 500–550 nm emission.

To evaluate the potential of C‐EcN_Arg_ in converting tumor‐accumulated ammonia into L‐arg, C‐EcN_Arg_ were resuspended in M9 minimal medium supplemented with 0.5% glucose and 5 mm NH_4_Cl at a density of 5 × 10^7^ CFU mL^−1^. Culture medium was replaced with fresh medium every 10 h within 50 h. L‐arg concentrations at 0, 10, 20, 30, 40, and 50 h were measured using a commercial L‐Arginine Assay Kit.

### 4.5 Growth Curves of EcN_Arg_ and C‐EcN_Arg_

To measure the bacterial growth curves, OD_600_ was monitored with a spectrophotometer and cuvettes. EcN_Arg_ and C‐EcN_Arg_ suspensions (100 µL, initial OD_600_ ≈ 0.2) were inoculated into 5 mL of fresh LB medium in a 15 mL sterile centrifuge tube and incubated at 37°C with shaking at 200 rpm. At 0, 1, 2, 3, 4, 5, 6, 7, and 8 h, 100 µL culture was withdrawn and transferred to a 10 mm path‐length quartz cuvette. OD_600_ was measured against a sterile LB medium blank control using a spectrophotometer, with the cuvette rinsed with distilled water and dried with lens paper after each measurement to prevent cross‐contamination. The experiment was performed in triplicate, and mean OD_600_ values at each time point were recorded.

### 4.6 Synthesis of MMP‐2‐Cleavable Peptide Cross‐Linker

Peptide containing MMP‐2 substrate (amino acid sequence: GPLGVRGK) was obtained from Nanjing Peptide Biotech Ltd. The MMP‐2‐cleavable peptide cross‐linker was synthesized by reacting GPLGVRGK with NAS. Briefly, 10 mg peptide (0.12 mm) was dissolved in 1 mL of borate buffer (20 mm, pH 8.5), and 21.6 mg NAS (0.6 mm) dissolved in 50 µL of anhydrous dimethyl sulfoxide (DMSO) was slowly added with stirring. The reaction proceeded under a nitrogen atmosphere with light exclusion at 25°C for 2 h. The resulting solution was dialyzed overnight against DI water to remove unreacted compounds and byproducts, lyophilized overnight, and stored at −20°C for later use. ESI‐MS: [M+H]: m/z: calcd. (C_40_H_66_N_12_O_11_): 890.49, found: 891.50.

### 4.7 Preparation and Characterization of C‐EcN_Arg+sPD‐1_ and DC‐EcN_Arg+sPD‐1_

To synthesize non‐degradable bacterial capsule (C‐EcN_Arg+sPD‐1_), EcN_Arg+sPD‐1_ (1 × 10^8^ CFU mL^−1^)) was resuspended in 888 µL of fresh sterile ice‐cold PBS. The following monomer solutions were then added sequentially: 100 µL MPC (50 mg mL^−1^ in PBS) and 10 µL BIS (50 mg mL^−1^ in DMSO). After incubation for 10 min, polymerization was initiated by adding 1 µL APS (100 mg mL^−1^ in water) and 1 µL TEMED (775 mg mL^−1^ in water). The mixture was maintained at 4–8°C and shaking for 2 h. Encapsulated EcN_Arg+sPD‐1_ (C‐EcN_Arg+sPD‐1_) were collected by centrifugation, washed with sterile ice‐cold PBS, and stored at 4°C for subsequent use. Degradable bacterial capsule (DC‐EcN_Arg+sPD‐1_) was synthesized by replacing BIS with an equimolar amount of degradable peptide cross‐linker (Ac‐GPLGVRGK‐Ac, 10 µL, 289 mg mL^−1^ in PBS). The successful preparation of C‐EcN_Arg+sPD‐1_ and DC‐EcN_Arg+sPD‐1_ was characterized using DLS and TEM measurements.

### 4.8 MMP‐2‐Responsive Degradation of Surface Polymer Networks

To directly observe the degradation of the polymer network of DC‐EcN_Arg+sPD‐1_, DC‐EcN_RFP_ was prepared with a similar synthesis method of C‐EcN_RFP_ by replacing BIS with an equimolar amount of degradable peptide cross‐linker (10 µL, 289 mg mL^−1^ in PBS). Unreacted monomers were removed by centrifugation (3500 g, 5 min, 4°C), C‐EcN_RFP_ and DC‐EcN_RFP_ at a density of 1 × 10^7^ CFU mL^−1^ were then resuspended in PBS with or without 10 nm MMP‐2. After 4 h incubation, C‐EcN_RFP_ and DC‐EcN_RFP_ were observed using CLSM (ZEISS, Germany), RFP: 561 nm excitation, 580–620 nm emission; FITC: 488 nm excitation, 500–550 nm emission.

### 4.9 On‐Demand Proliferation of DC‐EcN_Arg+sPD‐1_ in Response to MMP‐2

To demonstrate the on‐demand proliferation of DC‐EcN_Arg+sPD‐1_, EcN_Arg+sPD‐1_, C‐EcN_Arg+sPD‐1_, and DC‐EcN_Arg+sPD‐1_ at a density of 1 × 10^7^ CFU mL^−1^ were suspended in LB broth with or without 10 nm MMP‐2, respectively. After 4 h incubation, these bacterial suspensions (100 µL, initial OD_600_ ≈ 0.2) were resuspended into 5 mL of fresh LB medium in a 15 mL sterile centrifuge tube and incubated at 37°C with shaking at 200 rpm. At 0, 1, 2, 3, 4, 5, 6, 7, and 8 h, 100 µL culture medium was withdrawn and transferred to a 10 mm path‐length quartz cuvette. OD_600_ was measured against a sterile LB medium blank control using a spectrophotometer, with the cuvette rinsed with distilled water and dried with lens paper after each measurement to prevent cross‐contamination. The experiment was performed in triplicate, and mean OD_600_ values at each time point were recorded. For direct observation, C‐EcN_RFP_, and DC‐EcN_RFP_ were used. Briefly, C‐EcN_RFP_, and DC‐EcN_RFP_ at a density of 1 × 10^7^ CFU mL^−1^ were suspended in LB broth with or without 10 nm MMP‐2, respectively. After 4 h incubation, 100 µL samples were plated on LB agarose plates containing 100 µg mL^−1^ kanamycin. After 24 h incubation, LB agarose plates were imaged using an IVIS system (Spectral Instruments).

### 4.10 Antitumor Efficacy of DC‐EcN_Arg+sPD‐1_

To evaluate the antitumor efficacy of DC‐EcN_Arg+sPD‐1_, B16F10‐bearing mice were established by subcutaneous implantation of 1 × 10^6^ cells into the left flank of 6–8 weeks old female C57BL/6 mice. Seven days post‐implantation, mice were randomized into five groups (six mice per group, n = 6): PBS, C‐EcN_Arg_+αPD‐L1 (1 × 10^7^ CFU EcN_Arg_ and 200 µg αPD‐L1 per mouse), EcN_Arg+sPD‐1_ (1 × 10^7^ CFU EcN_Arg+sPD‐1_ per mouse), C‐EcN_Arg+sPD‐1_ (1 × 10^7^ CFU EcN_Arg+sPD‐1_ per mouse), and DC‐EcN_Arg+sPD‐1_ (1 × 10^7^ CFU EcN_Arg+sPD‐1_ per mouse). The αPD‐L1 was administrated at 24 h after bacterial treatment. All treatments were administered every 3 days for a total of 4 doses over 12 days. Tumor volume (calculated as V = 0.5 × length × width^2^) and body weight were monitored every 3 days.

For flow cytometric analysis, tumor tissues were minced, homogenized, and diluted with PBS at a weight ratio of 1:10. To evaluate T cell‐mediated antitumor immunity activation, single‐cell suspensions prepared from tumor tissues were stained with the following antibody panels: FITC‐conjugated anti‐mouse CD4, APC‐conjugated anti‐mouse CD3, PE‐conjugated anti‐mouse CD8, APC/Cy7‐conjugated anti‐mouse CD45, PE‐conjugated anti‐mouse CD45, BV605‐conjugated anti‐mouse IFN‐γ, FITC‐conjugated anti‐mouse Ki67, APC‐conjugated anti‐mouse CD11c, PE‐conjugated anti‐mouse CD86, FITC‐conjugated anti‐mouse CD80, BV605‐conjugated anti‐mouse CD11b, APC/Cy7‐conjugated anti‐mouse CD49b, APC/Cy7‐conjugated anti‐mouse NK1.1, BV510‐conjugated anti‐mouse F4/80, APC‐conjugated anti‐mouse Gr‐1, FITC‐conjugated anti‐mouse CD206, BV421‐conjugated anti‐mouse Foxp3. Prior to intracellular staining for Ki‐67, Foxp3, and IFN‐γ, tumor cells were fixed and permeabilized by incubation with 0.1% Triton X‐100 for 10 min. After staining, all suspensions were analyzed using flow cytometry.

To investigate the expression levels of tumor‐associated cytokines, IFN‐γ and TNF‐α in the supernatant of tumor homogenates were then measured using ELISA assay kits. For H&E and immunofluorescence staining, tumors were fixed with 4% paraformaldehyde and sliced to sections. Next, tumor sections were stained with H&E and a TUNEL staining kit, respectively. Immunofluorescence staining was performed on a Nikon AlR‐SIMe confocal microscope, and H&E‐stained slides were scanned using a slide scanner microscope (3DHISTECH).

### 4.11 Tumor Rechallenge and Metastasis Experiments

To investigate the long‐term immune memory effects of combinational therapy, a rechallenged tumor model was established. Briefly, B16F10‐bearing mice were treated on day 6, 8, and 10 as described previously. Primary tumors were surgically resected on day 13 post‐tumor inoculation. Mice were then rechallenged by subcutaneous injection of 1 × 10^6^ B16F10 cells into the contralateral (right) flank. Rechallenged tumor volumes were monitored for 19 days post‐rechallenge. To assess the activation of T cell‐based antitumor immunity in rechallenged tumors, rechallenged tumors were harvested and stained with FITC‐conjugated anti‐CD4, APC‐conjugated anti‐mouse CD3, PE‐conjugated anti‐mouse CD8, and APC/Cy7‐conjugated anti‐mouse CD45 for cytometric analysis.

To evaluate the antimetastatic potential of DC‐EcN_Arg+sPD‐1_, B16F10‐bearing mice were treated on day 6, 8, and 10 as described previously. At day 13 post‐tumor inoculation, the mice were intravenously challenged with B16F10 cells. To evaluate lung metastasis, lungs were harvested at day 25 post‐tumor inoculation, metastatic tumor nodules on the lung surface were counted, and analyzed by H&E staining. To analyze memory T cells, spleens were harvested and stained with APC/Cy7‐conjugated anti‐mouse CD45, APC‐conjugated anti‐mouse CD3, FITC‐conjugated anti‐mouse CD4, PE‐conjugated anti‐mouse CD8, FITC‐conjugated anti‐mouse CD44, and APC‐conjugated anti‐mouse CD62L, followed by flow cytometry analysis.

### 4.12 Statistical Analysis

All data were represented as mean ± standard deviation (mean ± s.d.) from more than three independent experiments (*n* ≥ 3). Statistical comparisons were performed using Student's *t*‐test for two‐group comparisons, and one‐way analysis of variance (ANOVA) with Tukey's test or two‐way ANOVA with Sidak's test for multiple comparisons where appropriate. All statistical analysis were obtained using GraphPad Prism (Prism 10.1.2). Flow cytometric data were analyzed by FlowJo software (v10.8.1). The significant levels are shown as ns > 0.05, *p < 0.05, **p < 0.01, ***p < 0.001, and ****p < 0.0001 as indicated.

## Author Contributions

**Jianhui Yang**: conceptualization, software, methodology, data curation, investigation, formal analysis, writing – original draft, visualization. **Ao Peng**: methodology, software, data curation, investigation, formal analysis, visualization. **Shuaiqiang Li**: data curation, methodology. **Yaning Huang**: software, data curation. **Jian Yan**: methodology, investigation, formal analysis, data curation. **Leyuan Wang**: methodology, data curation, investigation. **Zhihui Zhu**: methodology, data curation, investigation. **Fei‐Long Liu**: investigation. **Erbao Bian**: software, data curation, formal analysis. **Yang Liu**: writing – review and editing, funding acquisition, supervision. **Dasheng Tian**: writing – review and editing, funding acquisition, validation, resources. **Fenghe Li**: funding acquisition, writing – review and editing, project administration, validation. **Qi Liu**: conceptualization, writing – original draft, writing – review and editing, project administration, resources, supervision, funding acquisition, visualization.

## Conflicts of Interest

The authors declare no conflicts of interest.

## Supporting information




**Supporting File**: advs76706‐sup‐0001‐SuppMat.pdf.

## Data Availability

The data that supports the findings of this study are available in the supplementary material of this article.
