# Supplementary material for: Programmable Encapsulation Enables On‐Demand Proliferation of Therapeutic Bacteria for Potent Cancer Immunotherapy
Source: Adv Sci (Weinh). 2026 Jul 23;13(57):e76706. doi: 10.1002/advs.76706 (PMC13393283; doi:10.1002/advs.76706)
Supplement: Supplementary file 1 [file ADVS-13-e76706-s001.pdf]

Supporting Information

Programmable Encapsulation Enables On-Demand Proliferation of Therapeutic Bacteria for Potent Cancer Immunotherapy

Jianhui Yang[1], Ao Peng[1], Shuaiqiang Li[1], Yaning Huang[2], Jian Yan[1], Leyuan Wang[1], Zhihui Zhu[1], Fei-Long Liu[1], Erbao Bian[2], Yang Liu[3,*], Dasheng Tian[2,*], Fenghe Li[1,*], Qi Liu[1,*]

[1]School of Pharmacy, Anhui Medical University, Hefei 230032, China
[2]Department of Orthopedics, The Second Affiliated Hospital of Anhui Medical University, Hefei 230032, China
[3]State Key Laboratory of Medicinal Chemical Biology, Key Laboratory of Functional Polymer Materials Ministry of Education, College of Chemistry, Nankai University

Jianhui Yang and Ao Peng contributed equally to this work.

Supplementary Text

Materials

All commercial reagents and solvents were used as received without further purification unless otherwise specified. N-(3-Aminopropyl) methacrylamide hydrochloride (APm) and 2-methacryloyloxyethyl phosphorylcholine (MPC) were purchased from Energy Chemical (Shanghai, China). Ammonium persulfate (APS), N-acryloxysuccinimide (NAS), N,N'-methylene bisacrylamide (BIS), and N,N,N′,N′-tetramethylethylenediamine (TMEDA) were purchased from Aladdin (Shanghai, China). Dialysis membranes were purchased from Tiannan Technology (Tianjin, China). Fluorescein isothiocyanate (FITC) and Cy5.5-N-hydroxysuccinimide (Cy5.5-NHS) were purchased from Oukainasi Technology (Beijing, China). The Cell Counting Kit (CCK) was purchased from APExBIO Biotech (Shanghai, China). Antibodies for flow cytometric analysis, including FITC-conjugated anti-mouse CD4 (cat. no. 100406), APC-conjugated anti-mouse CD3 (cat. no. 100236), PE-conjugated anti-mouse CD8 (cat. no. 100708), APC/Cy7-conjugated anti-mouse CD45 (cat. no. 103116), PE-conjugated anti-mouse CD45 (cat. no. 103105), Brilliant Violet (BV) 605-conjugated anti-mouse IFN-γ (cat. no. 505840), FITC-conjugated anti-mouse Ki67 (cat. no. 151211), APC-conjugated anti-mouse CD11c (cat. no. 117310), PE-conjugated anti-mouse CD86 (cat. no. 105008), FITC-conjugated anti-mouse CD80 (cat. no. 104706), BV605-conjugated anti-mouse CD11b (cat. no. 101257), APC/Cy7-conjugated anti-mouse CD49b (cat. no. 108919), APC/Cy7-conjugated anti-mouse NK1.1 (cat. no. 108723), BV510-conjugated anti-mouse F4/80 (cat. no. 123135), APC-conjugated anti-mouse Gr-1 (cat. no. 108411), FITC-conjugated anti-mouse CD206 (cat. no. 141703), Brilliant Violet (BV) 421-conjugated anti-mouse Foxp3 (cat. no. 126419), FITC-conjugated anti-mouse CD44 (cat. no. 103006), and APC-conjugated anti-mouse CD62L (cat. no. 104412) were purchased from Biolegend (San Diego, USA). ELISA Assay kits for anti-mouse IFN-γ (cat. no. 2210002), anti-mouse IL-6 (cat. no. 1210602), and anti-mouse TNF-α (cat. no. 1217202) were obtained from Dakewe Biotech (Shenzhen, China). DAPI and D-Luciferin sodium salt D were purchased from Solarbio Science & Technology (Beijing, China). The Viability/Cytotoxicity Assay Kit for bacteria was obtained from UElandy Biotech (Suzhou, China). Anti-mouse PD-L1 monoclonal antibody (cat. no. BE0101) was obtained from BioXcell (New Hampshire, USA). Anti-mouse PD-1 monoclonal antibody (cat. no. 31445-1-AP) was obtained from Proteintech (Wuhan, China). Anti-mouse GAPDH (catalog no. GB15002) was purchased from Servicebio (Wuhan, China). Fluorescent TUNEL Staining Kit was purchased from Yeasen Biotech (Shanghai, China). L-Arginine Assay Kit was obtained from Solarbio (Beijing, China).

Bacteria Strains and Functional Plasmids

Attenuated *Escherichia coli* Nissle 1917 (EcN) and EcN_{RFP} used in this study was obtained from MiaoLing Technology (Wuhan, China). To construct EcN_{Arg} that can convert tumor-accumulated ammonia, a byproduct of glutamine metabolism, to therapeutically active L-arg, pBF-Arg-M215 plasmid was constructed by inserting the encoding sequence of ArgA and Arg box into the pBF-pTrc-B0035 plasmid using the multiple cloning site. Thereafter, pBF-Arg-M215 plasmid was transformed into EcN through a heat shock transformation method, yielding engineered EcN_{Arg}. To construct EcN_{Arg} that can simultaneously produce L-arg and secrete sPD-1, pBF-Arg+sPD-1 plasmid was constructed by encoding the extracellular domain protein of mouse PD-1 sequence (amino acids 1 to 169, UniProt accession number Q02242) with a pelB signal peptide sequence at the N-terminus into the pBF-Arg-M215 plasmid using the multiple cloning site. Next, pBF-Arg+sPD-1 plasmid was transformed into EcN through a heat shock transformation method, yielding engineered $EcN_{Arg+sPD-1}$. All plasmids were designed using SnapGene (GSL Biotech) and assembled using Gibson Assembly reagents from New England Biolabs (E2621L). Additional genes and all PCR primers were synthesized by Sangon Biotech.

Bacterial Culture for *In Vitro* and *In Vivo* Experiments

All bacteria were cultured in LB broth and grown on LB agar plates supplemented with appropriate antibiotics. Single colonies were picked and inoculated into LB broth, followed by overnight incubation in a shaking incubator (37°C, 200 rpm). For antibiotic selection, kanamycin (Beyotime Biotech, Shanghai, China) was added to the culture medium at a final concentration of 10 μg mL^{-1}. Next day, the optical density at 600 nm (OD_{600}) was measured, and saturated cultures were diluted to an OD_{600} of 0.2. Diluted cultures were grown to the exponential phase (OD_{600} = 1.5) prior to assays. OD_{600} measurements were performed in triplicate using a UV1900 spectrophotometer (SHIMADZU, Japan) in cuvette mode to ensure reproducibility.

Characterization of Engineered Bacterial Strains

Glycerol cryostocks of EcN_{Arg} and $EcN_{Arg+sPD-1}$ stored at -80°C were thawed on ice, and 10 μL aliquots were inoculated into 10 mL of antibiotic-free 2YT medium. Cultures were aerobically incubated at 37°C with shaking (200 rpm) for 3 h to promote bacterial recovery, bacterial concentrations were standardized to 1×10^8 CFU mL^{-1} based on OD_{600} measurements and validated by standard plate counting. To evaluate the potential of EcN_{Arg} and $EcN_{Arg+sPD-1}$ in converting tumor-accumulated ammonia into therapeutically active L-arg, non-engineered EcN,

EcN_{Arg}, and $EcN_{Arg+sPD-1}$ were resuspended in M9 minimal medium supplemented with 0.5% glucose and 5 mM NH_4Cl at a density of 5×10^7 CFU mL^{-1}, and 10 mL aliquots were dispensed into sterile test tubes. Samples were collected 4 h post-resuspension and centrifuged (10000 g, 5 min, 4°C), and then transferred to 1.5 mL microcentrifuge tubes for L-arg quantification using a commercial L-Arginine Assay Kit.

To detect sPD-1 protein secretion, Western blot assay was used. Briefly, $EcN_{Arg+sPD-1}$ (5×10^7 CFU mL^{-1}) was suspended in LB broth. After 24 h incubation, supernatants were collected by centrifugation (10000 g, 5 min, 4°C) and then separated by SDSPAGE followed by transfer onto nitrocellulose membranes (Millipore). Membranes were blocked using PBS containing 5% milk and 0.1% Tween 20 and detected using primary anti-mouse PD-1 (dilution 1:1000) and anti-mouse GAPDH (dilution 1:2000). After incubation with secondary anti-rabbit conjugated with HRP (Sigma-Aldrich), membranes were washed and reacted with chemiluminescence substrate (Millipore) for visualization using ChemiDoc (BioRad, USA). GAPDH in pellet was employed as control.

Cell Culture

All cell lines were procured from the American Type Culture Collection (ATCC). 4T1 murine breast cancer cells were maintained in Dulbecco's Modified Eagle Medium (DMEM) supplemented with 10% (v/v) fetal bovine serum (FBS). B16F10 murine melanoma cells were cultured in Roswell Park Memorial Institute (RPMI)-1640 medium containing 10% (v/v) FBS, 100 U mL^{-1} penicillin, and 100 μg mL^{-1} streptomycin (Biochannel). Cultures were maintained at 37°C in a humidified 5% CO_2 incubator, with medium changes every 2-3 days and passaging at 80% confluence using 0.25% trypsin-EDTA (Biochannel).

Animals

All experimental protocols were conducted within Anhui Medical University guidelines for animal research and approved by Animal Care and Use Committee (LLSC20230877).

Antitumor Efficacy of EcN_{Arg} Combined with αPD-L1

To assess the antitumor efficacy of EcN_{Arg} in combination with αPD-L1, 4T1 tumor-bearing mice were established by subcutaneous implantation of 1×10^6 cells into the left flank of 6-8 weeks old female BALB/c mice. Treatments were administered intravenously (i.v.) on day 7, 10, 13, and 16 (total of 4 doses) post-tumor inoculation. Seven days post-inoculation, tumor-bearing mice were randomized into six groups (six mice per group, n = 6), and intravenously injected with PBS, EcN (1×10^7 CFU EcN per mouse), EcN_{Arg} (1×10^7 CFU EcN_{Arg} per mouse),

αPD-L1 (200 μg αPD-L1 per mouse), EcN+αPD-L1 (1×10^7 CFU EcN and 200 μg αPD-L1 per mouse), and EcN_{Arg}+αPD-L1 (1×10^7 CFU EcN_{Arg} and 200 μg αPD-L1 per mouse). The αPD-L1 was administrated at 24 h after bacterial treatment. Tumor volume (calculated as $V = 0.5 \times length \times width^2$) and body weight were monitored every 3 days.

For flow cytometric analysis, tumor tissues were collected, homogenized, and diluted with PBS at a weight ratio of 1:10. To evaluate T cell-mediated antitumor immune responses, tumor suspensions were stained with APC/Cy7-conjugated anti-mouse CD45, APC-conjugated anti-mouse CD3, FITC-conjugated anti-mouse CD4, PE-conjugated anti-mouse CD8, BV605-conjugated anti-mouse IFN-γ, and FITC-conjugated anti-mouse Ki-67. For anti-mouse Ki-67 and anti-mouse IFN-γ staining, tumor suspensions were pretreated with 0.1% Triton X-100 for 10 min. After staining, all suspensions were analyzed by flow cytometry. Tumor sections were stained with H&E and TUNEL Staining Kit, respectively. Immunofluorescence staining was imaged using a Nikon AlR-SIMe confocal microscope, and H&E-stained slides were scanned using a slide scanner microscope (3DHISTECH).

***In Vivo* Biosafety Evaluation of EcN_{Arg}**

For safety evaluation, healthy BALB/c mice were randomized into six groups (six mice per group, n = 6) and intravenously injected with PBS, EcN (1×10^7 CFU EcN per mouse), EcN_{Arg} (1×10^7 CFU EcN_{Arg} per mouse), αPD-L1 (200 μg αPD-L1 per mouse), EcN+αPD-L1 (1×10^7 CFU EcN and 200 μg αPD-L1 per mouse), and EcN_{Arg}+αPD-L1 (1×10^7 CFU EcN_{Arg} and 200 μg αPD-L1 per mouse). The αPD-L1 was administrated at 24 h after bacterial treatment. Treatments were administered on days 0, 2, 4, and 6 (total of 4 doses). Blood and major organs were collected from mice on day 8 after the first injection. Serum was separated by centrifugation at 2500 g for 10 min, and blood counts, including WBC, NEUT, PLT, were measured using an automated hematology analyzer (Sysmex XT-2000i), with reference ranges validated against murine hematological standards. Serum biochemical parameters, including ALP, ALT, AST, CK, LDH, CREA, and UREA were determined using a clinical chemistry analyzer following the manufacturer's protocols. Major organs (heart, liver, spleen, lung, and kidney) were fixed in 4% paraformaldehyde, sectioned, and subjected to H&E staining, with slides scanned using a slide scanner microscope (3DHISTECH).

Preparation and Characterization of C-EcN_{Arg}

To synthesize C-EcN_{Arg}, 1 mL EcN_{Arg} suspension (1×10^8 CFU mL^{-1}) was centrifuged (3500 g, 5 min, 4°C) in a 1.5 mL sterile microcentrifuge tube, and the supernatant was discarded. The pellet was resuspended in 888 μL of fresh sterile ice-cold PBS. The following monomer

solutions were then added sequentially: 100 μL MPC (50 mg mL^{-1} in PBS), and 10 μL BIS (50 mg mL^{-1} in DMSO). After incubation for 10 min at 4°C, polymerization was initiated by adding 1 μL APS (100 mg mL^{-1} in water) and 1 μL TEMED (775 mg mL^{-1} in water). The mixture was maintained at 4-8°C and shaking (200 rpm) for 2 h. Encapsulated bacteria (C-EcN_{Arg}) were collected by centrifugation, washed with sterile ice-cold PBS, and stored at 4°C for subsequent use.[1-3]

Dynamic light scattering (DLS) was used to measure the size distribution and zeta potential of EcN_{Arg} and C-EcN_{Arg} using a Zetasizer Nano ZS (Malvern Instruments), with each sample analyzed in triplicate and averaged. For transmission electron microscopy (TEM) observation, EcN_{Arg} and C-EcN_{Arg} were prepared as described, diluted in deionized water to 1×10^7 CFU mL^{-1}, and 10 μL sample solutions were dropped onto a copper grid, allowed to stand for 10 min, and excess liquid was blotted off with filter paper. This process was repeated, followed by staining with 10 μL 2% phosphotungstic acid for 5 seconds. Excess liquid was removed with filter paper, and grids were vacuum-dried for 24 h before imaging on a JEM-2100 TEM (JEOL) at 200 kV.

To demonstrate the successful polymerization on single bacterial surface, a fluorescent labeled monomers (Ac-FITC) was synthesized. Briefly, FITC (778 mg, 2.0 mmol) was dissolved in dichloromethane (20 mL), APm (426 mg, 3.0 mmol) were added under ice bath condition and stirred for 2 h. The solvent was then removed through reduced pressure concentration, and the crude product was purified using a C18 reverse phase column (MeOH/DCM = 1/15) to obtain yellow solid Ac-FITC (yield, 968.5 mg, 91%). ^{1}H NMR (400 MHz, DMSO-d_6) δ 10.13 (s, 3H), 8.23 (d, J = 2.0 Hz, 1H), 8.17 (s, 1H), 8.00 (t, J = 5.8 Hz, 1H), 7.75 (d, J = 8.3 Hz, 1H), 7.19 (d, J = 8.3 Hz, 1H), 6.68 (d, J = 2.3 Hz, 2H), 6.64-6.54 (m, 4H), 5.67 (t, J = 1.3 Hz, 1H), 5.35-5.31 (m, 1H), 3.60-3.49 (m, 2H), 3.22-3.16 (m, 2H), 1.90-1.82 (m, 3H), 1.74 (p, J = 6.9 Hz, 2H). ESI-MS: [M+H]: m/z: calcd. ($C_{28}H_{25}N_3O_6S$): 532.15, found: 532.10.

To directly observe polymer network on bacterial surface, Ac-FITC and RFP-labelled EcN_{RFP} were employed to synthesize C-EcN_{RFP}. Briefly, 1 mL EcN_{RFP} suspension (1×10^8 CFU) was centrifuged (3500 g, 5 min, 4°C) in a 1.5 mL sterile microcentrifuge tube, and the supernatant was discarded. The pellet was resuspended in 888 μL of fresh ice-cold PBS. The following monomer solutions were then added sequentially: 80 μL MPC (50 mg mL^{-1} in PBS), 20 μL Ac-FITC (100 mg mL^{-1} in PBS), and 10 μL BIS (50 mg mL^{-1} in DMSO). After incubation for 10 min, polymerization was initiated by adding 1 μL APS (100 mg mL^{-1} in water) and 1 μL TEMED (775 mg mL^{-1} in water). The mixture was maintained at 4-8°C and shaking for 4 h.

Unreacted monomers were removed by centrifugation, C-EcN$_{RFP}$ were observed using confocal laser scanning microscope (ZEISS, Germany), RFP: 561 nm excitation, 580-620 nm emission; FITC: 488 nm excitation, 500-550 nm emission.

To evaluate the potential of C-EcN$_{Arg}$ in converting tumor-accumulated ammonia into L-arg, C-EcN$_{Arg}$ were resuspended in M9 minimal medium supplemented with 0.5% glucose and 5 mM NH_4Cl at a density of 5×10^7 CFU mL^{-1}. Culture medium was replaced with fresh medium every 10 h within 50 h. L-arg concentrations at 0, 10, 20, 30, 40, and 50 h were measured using a commercial L-Arginine Assay Kit.

Bacteria Viability Assays

Live and dead bacteria were visualized using a live/dead bacterial staining kit following the manufacturer's protocols. The staining stock solution was prepared by adding 1 μL NucGreen and 2 μL EthD-III into 8 μL 0.85% (m/m) NaCl. Briefly, 1 mL bacterial suspension (EcN$_{Arg}$ and C-EcN$_{Arg}$) in PBS (density: 1×10^8 CFU mL^{-1}) was seeded into a 1.5 mL centrifuge tube, mixed with 10 μL of the prepared stock solution, and incubated at 37°C for 20 min in the dark. After incubation, bacteria were visualized using CLSM with excitation/emission wavelengths set at 488/525 nm (green for live bacteria) and 561/620 nm (red for dead bacteria).

Bacterial viability was quantitatively analyzed using CCK assay. A volume of 180 μL bacterial suspension (EcN$_{Arg}$ and C-EcN$_{Arg}$) in culture medium (density: 1.8×10^7 CFU mL^{-1}) was seeded into 96-well plates, and 20 μL CCK reagent was added to each well. Plates were incubated at 37°C for 2 h in a 5% CO_2 incubator. Following 2 h incubation, the optical density (OD) at 450 nm was measured using a microplate reader (Benchmark Plus, Bio-Rad), and bacterial viability was calculated relative to the control group.

Growth Curves of EcN$_{Arg}$ and C-EcN$_{Arg}$

To measure the bacterial growth curves, OD_{600} was monitored with a spectrophotometer and cuvettes. EcN$_{Arg}$ and C-EcN$_{Arg}$ suspensions (100 μL, initial $OD_{600} \approx 0.2$) was inoculated into 5 mL of fresh LB medium in a 15 mL sterile centrifuge tube and incubated at 37°C with shaking at 200 rpm. At 0, 1, 2, 3, 4, 5, 6, 7, and 8 h, 100 μL culture was withdrawn and transferred to a 10 mm path-length quartz cuvette. OD_{600} was measured against a sterile LB medium blank control using a spectrophotometer, with the cuvette rinsed with distilled water and dried with lens paper after each measurement to prevent cross-contamination. Experiment was performed in triplicate, and mean OD_{600} values at each time point were recorded.

Synthesis of MMP-2-Cleavable Peptide Crosslinker

Peptide containing MMP-2 substrate (amino acid sequence: GPLGVRGK) was obtained from Nanjing Peptide Biotech Ltd. The MMP-2-cleavable peptide crosslinker was synthesized by reacting GPLGVRGK with NAS. Briefly, 10 mg peptide (0.12 mM) was dissolved in 1 mL of borate buffer (20 mM, pH 8.5), and 21.6 mg NAS (0.6 mM) dissolved in 50 μL of anhydrous dimethyl sulfoxide (DMSO) was slowly added with stirring. Reaction proceeded under nitrogen atmosphere with light exclusion at 25°C for 2 h. The resulting solution was dialyzed overnight against DI water to remove unreacted compounds and by-products, lyophilized overnight, and stored at -20°C for later use. ESI-MS: [M+H]: m/z: calcd. ($C_{40}H_{66}N_{12}O_{11}$): 890.49, found: 891.50.

Preparation and Characterization of C-EcN$_{Arg+sPD-1}$ and DC-EcN$_{Arg+sPD-1}$

To synthesize non-degradable bacterial capsule (C-EcN$_{Arg+sPD-1}$), EcN$_{Arg+sPD-1}$ (1 × 10^8 CFU mL^{-1}) resuspended in 888 μL of fresh sterile ice-cold PBS. The following monomer solutions were then added sequentially: 100 μL MPC (50 mg mL^{-1} in PBS) and 10 μL BIS (50 mg mL^{-1} in DMSO). After incubation for 10 min, polymerization was initiated by adding 1 μL APS (100 mg mL^{-1} in water) and 1 μL TEMED (775 mg mL^{-1} in water). The mixture was maintained at 4-8°C and shaking for 2 h. Encapsulated EcN$_{Arg+sPD-1}$ (C-EcN$_{Arg+sPD-1}$) were collected by centrifugation, washed with sterile ice-cold PBS, and stored at 4°C for subsequent use. Degradable bacterial capsule (DC-EcN$_{Arg+sPD-1}$) was synthesized by replacing BIS with an equimolar amount of degradable peptide crosslinker (Ac-GPLGVRGK-Ac, 10 μL, 289 mg mL^{-1} in PBS). The successful preparation of C-EcN$_{Arg+sPD-1}$ and DC-EcN$_{Arg+sPD-1}$ was characterized using DLS and TEM measurements.

MMP-2-Responsive Degradation of Surface Polymer Networks

To directly observe the degradation of polymer network of DC-EcN$_{Arg+sPD-1}$, DC-EcN$_{RFP}$ was prepared with a similar synthesis method of C-EcN$_{RFP}$ by replacing BIS with an equimolar amount of degradable peptide crosslinker (10 μL, 289 mg mL^{-1} in PBS). Unreacted monomers were removed by centrifugation (3500 g, 5 min, 4°C), C-EcN$_{RFP}$ and DC-EcN$_{RFP}$ at a density of 1 × 10^7 CFU mL^{-1} were then resuspended in PBS with or without 10 nM MMP-2. After 4 h incubation, C-EcN$_{RFP}$ and DC-EcN$_{RFP}$ were observed using CLSM (ZEISS, Germany), RFP: 561 nm excitation, 580-620 nm emission; FITC: 488 nm excitation, 500-550 nm emission.

On-Demand Proliferation of DC-EcN$_{Arg+sPD-1}$

To demonstrate the on-demand proliferation of DC-EcN$_{Arg+sPD-1}$, EcN$_{Arg+sPD-1}$, C-EcN$_{Arg+sPD-1}$, and DC-EcN$_{Arg+sPD-1}$ at a density of 1 × 10^7 CFU mL^{-1} were suspended in LB broth with or

without 10 nM MMP-2, respectively. After 4 h incubation, these bacterial suspension (100 μL, initial OD_{600} ≈ 0.2) were resuspended into 5 mL of fresh LB medium in a 15 mL sterile centrifuge tube and incubated at 37°C with shaking at 200 rpm. At 0, 1, 2, 3, 4, 5, 6, 7, and 8 h, 100 μL culture medium was withdrawn and transferred to a 10 mm path-length quartz cuvette. OD_{600} was measured against a sterile LB medium blank control using a spectrophotometer, with the cuvette rinsed with distilled water and dried with lens paper after each measurement to prevent cross-contamination. Experiment was performed in triplicate, and mean OD_{600} values at each time point were recorded. For direct observation, C-EcN_{RFP}, and DC-EcN_{RFP} were used. Briefly, C-EcN_{RFP}, and DC-EcN_{RFP} at a density of 1 × 10^7 CFU mL^{-1} were suspended in LB broth with or without 10 nM MMP-2, respectively. After 4 h incubation, 100 μL samples were plated on LB agarose plates containing 100 μg mL^{-1} kanamycin. After 24 h incubation, LB agarose plates were imaged using an IVIS system (Spectral Instruments).

MMP-2-Responsive Release of sPD-1

To demonstrate the MMP-2-responsive sPD-1 release, $EcN_{Arg+sPD-1}$, C-$EcN_{Arg+sPD-1}$, and DC-$EcN_{Arg+sPD-1}$ at a density of 5 × 10^7 CFU mL^{-1} were suspended in LB broth. After 24 h incubation, MMP-2 was added into the culture medium to a concentration of 10 nM and incubated for 4 h. To eliminate the impact of bacterial proliferation, bacterial density was uniformly diluted to 5 × 10^7 CFU mL^{-1}. Next, supernatants were collected by centrifugation (10000 g, 5 min, 4°C), and separated by SDS-PAGE followed by transfer onto nitrocellulose membranes (Millipore). Membranes were blocked and detected using primary anti-mouse PD-1 (dilution 1:1000). After incubation with the secondary anti-rabbit conjugated with HRP (Sigma-Aldrich), membranes were washed and reacted with chemiluminescence substrate (Millipore) for visualization using ChemiDoc (BioRad, USA).

Immunogenicity Evaluation of C-EcN_{Arg} and DC-$EcN_{Arg+sPD-1}$

To evaluate the immunogenicity of C-EcN_{Arg}, and DC-$EcN_{Arg+sPD-1}$, female BALB/c mice (6-8 weeks old) were randomized into five groups (three mice per group, n = 3) and intravenously injected with PBS, EcN_{Arg} (1 × 10^7 CFU), $EcN_{Arg+sPD-1}$ (1 × 10^7 CFU), C-EcN_{Arg} (1 × 10^7 CFU), and DC-$EcN_{Arg+sPD-1}$ (1 × 10^7 CFU), respectively. Treatments were administered every 3 days for a total of 4 doses over 12 days. Serum samples were collected from the retro-orbital plexus at days 13 post-injection. Mouse serum was separated by centrifugation at 3000g for 10 min at 4°C. Serum concentrations of TNF-α and IL-6 were quantified using commercial ELISA kits according to the manufacturer's instructions.

***In Vivo* Biodistribution and Pharmacokinetic Analysis**

Bacterial concentration was adjusted to 1×10^8 CFU mL^{-1}. Bacteria were labeled by incubation with Cy5.5-NHS (final concentration 1.5 μM) at 37°C for 30 min. Unreacted Cy5.5-NHS was removed by washing the bacteria three times with sterile PBS *via* centrifugation (3500 g, 5 min). Labeling efficiency was quantified by measuring bacterial fluorescence intensity (Ex: 680 nm, Em: 710 nm). Bacteria (1×10^7 CFU per mouse) were intravenously injected into 4T1-bearing mouse *via* the tail vein (n = 3). At specified time points, mice were imaged using an IVIS system (Spectral Instruments) to evaluate bacterial biodistribution and tumor accumulation. At 72 h post-injection, mice were sacrificed, and major organs (heart, lung, liver, kidney, spleen, and tumor) were collected for *ex vivo* fluorescence imaging.

To quantify bacterial counts in each organ (CFU per gram of tissue), organs (heart, liver, spleen, lung, kidney, and tumor) were collected at 6, 12, 24, 48, 72, and 120 h post-injection, weighed, homogenized in sterile PBS on ice, and the tissue homogenate was serially diluted (10-fold to 10000-fold) in PBS. A 100 μL aliquot of the homogenate was plated on LB plates containing 100 μg mL^{-1} kanamycin. Colonies were counted after incubation at 37°C for 12 h.

To assess the pharmacokinetic of C-EcN_{Arg}, female BALB/c mice were randomized into two groups (three mice per group, n = 3) and received tail vein injection of 100 μL Cy5.5-labeled EcN_{Arg} and C-EcN_{Arg} (1×10^7 CFU). Blood samples were collected at 0, 4, 8, 12, 18, 24, 36, and 48 h post-injection, serum was separated by centrifugation at 3000 g for 10 min at 4°C. Next, serum fluorescence intensity (Ex: 680 nm, Em: 710 nm) was quantified in triplicate using a fluorescence spectrophotometer and normalized to PBS as a blank control.

***In Vivo* Biosafety Evaluation of C-EcN_{Arg} and DC-$EcN_{Arg+sPD-1}$**

For safety evaluation, BALB/c mice were randomized into five groups (four mice per group, n = 4) and intravenously injected with PBS, EcN (1×10^7 CFU), EcN_{Arg} (1×10^7 CFU), C-EcN_{Arg} (1×10^7 CFU), $EcN_{Arg+sPD-1}$ (1×10^7 CFU), and DC-$EcN_{Arg+sPD-1}$ (1×10^7 CFU), respectively. Treatments were administered on day 0, 2, 4, and 6 (total of 4 doses). Blood and major organs were collected from mice on day 8 after first injection. Serum was separated by centrifugation at 2500 g for 10 min at 4°C, and blood counts, including WBC, NEUT, and PLT counts, were measured using an automated hematology analyzer (Sysmex XT-2000i), with reference ranges validated against murine hematological standards. Serum biochemical parameters, including HGB, HCT, AST, BUN, ALP, CRE, and LDH were determined using a clinical chemistry analyzer following the manufacturer's protocols. Organs (heart, liver, spleen, lung, and kidney) were fixed in 4% paraformaldehyde, sectioned, and subjected to H&E staining, with slides scanned using a slide scanner microscope (3DHISTECH).

Antitumor Efficacy of C-EcN$_{Arg}$ Combined with αPD-L1

To evaluate the antitumor efficacy of C-EcN$_{Arg}$ in combination with αPD-L1, breast cancer and melanoma model were firstly constructed through subcutaneous implantation of 1×10^6 4T1 or B16F10 cells into the left flank of 6-8 weeks old female BALB/c or C57BL/6 mice, respectively. Seven days post-implantation, mice were randomized into five groups (six mice per group, n = 6): PBS, αPD-L1 (200 μg αPD-L1 per mouse), EcN$_{Arg}$+αPD-L1 (1×10^7 CFU EcN$_{Arg}$ and 200 μg αPD-L1 per mouse), C-EcN$_{Arg}$ (1×10^7 CFU EcN$_{Arg}$), and C-EcN$_{Arg}$+αPD-L1 (1×10^7 CFU EcN$_{Arg}$ and 200 μg αPD-L1). The αPD-L1 was administrated at 24 h after bacterial treatment. All treatments were administered every 3 days for a total of 4 doses. Tumor volume (calculated as $V = 0.5 \times \text{length} \times \text{width}^2$) and body weight were monitored every 3 days.

For flow cytometric analysis, tumor tissues were minced, homogenized, and diluted with PBS at a weight ratio of 1:10. To evaluate T cell-mediated antitumor immunity activation, single-cell suspensions prepared from tumor tissues were stained with the following antibody panels: FITC-conjugated anti-mouse CD4, APC-conjugated anti-mouse CD3, PE-conjugated anti-mouse CD8, APC/Cy7-conjugated anti-mouse CD45, PE-conjugated anti-mouse CD45, BV605-conjugated anti-mouse IFN-γ, FITC-conjugated anti-mouse Ki67, APC-conjugated anti-mouse CD11c, PE-conjugated anti-mouse CD86, FITC-conjugated anti-mouse CD80, BV605-conjugated anti-mouse CD11b, APC/Cy7-conjugated anti-mouse CD49b (4T1 model), APC/Cy7-conjugated anti-mouse NK1.1 (B16F10 model), BV510-conjugated anti-mouse F4/80, APC-conjugated anti-mouse Gr-1, FITC-conjugated anti-mouse CD206, BV421-conjugated anti-mouse Foxp3. Prior to intracellular staining for Ki-67, Foxp3, and IFN-γ, tumor cells were fixed and permeabilized by incubation with 0.1% Triton X-100 for 10 min. After staining, all suspensions were analyzed by flow cytometry.

To investigate the expression levels of tumor associated cytokines, IFN-γ and TNF-α in the supernatant of tumor homogenates were then measured using ELISA assay kits. For H&E and immunofluorescence staining, tumors were fixed with 4% paraformaldehyde and sliced to sections. Next, tumor sections were stained with H&E and TUNEL staining kit, respectively. Immunofluorescence staining was performed on a Nikon AlR-SIMe confocal microscope, and H&E-stained slides were scanned using a slide scanner microscope (3DHISTECH).

Antitumor Efficacy of DC-EcN$_{Arg+sPD-1}$

To evaluate the antitumor efficacy of DC-EcN$_{Arg+sPD-1}$, B16F10-bearing mice were established by subcutaneous implantation of 1×10^6 cells into the left flank of 6-8 weeks old female C57BL/6 mice. Seven days post-implantation, mice were randomized into five groups (six mice

per group, n = 6): PBS, C-EcN_{Arg}+αPD-L1 (1×10^7 CFU EcN_{Arg} and 200 μg αPD-L1 per mouse), $EcN_{Arg+sPD\text{-}1}$ (1×10^7 CFU $EcN_{Arg+sPD\text{-}1}$ per mouse), C-$EcN_{Arg+sPD\text{-}1}$ (1×10^7 CFU $EcN_{Arg+sPD\text{-}1}$ per mouse), and DC-$EcN_{Arg+sPD\text{-}1}$ (1×10^7 CFU $EcN_{Arg+sPD\text{-}1}$ per mouse). The αPD-L1 was administrated at 24 h after bacterial treatment. All treatments were administered every 3 days for a total of 4 doses over 12 days. Tumor volume (calculated as $V = 0.5 \times \text{length} \times \text{width}^2$) and body weight were monitored every 3 days.

For flow cytometric analysis, tumor tissues were minced, homogenized, and diluted with PBS at a weight ratio of 1:10. To evaluate T cell-mediated antitumor immunity activation, single-cell suspensions prepared from tumor tissues were stained with the following antibody panels: FITC-conjugated anti-mouse CD4, APC-conjugated anti-mouse CD3, PE-conjugated anti-mouse CD8, APC/Cy7-conjugated anti-mouse CD45, PE-conjugated anti-mouse CD45, BV605-conjugated anti-mouse IFN-γ, FITC-conjugated anti-mouse Ki67, APC-conjugated anti-mouse CD11c, PE-conjugated anti-mouse CD86, FITC-conjugated anti-mouse CD80, BV605-conjugated anti-mouse CD11b, APC/Cy7-conjugated anti-mouse CD49b, APC/Cy7-conjugated anti-mouse NK1.1, BV510-conjugated anti-mouse F4/80, APC-conjugated anti-mouse Gr-1, FITC-conjugated anti-mouse CD206, BV421-conjugated anti-mouse Foxp3. Prior to intracellular staining for Ki-67, Foxp3, and IFN-γ, tumor cells were fixed and permeabilized by incubation with 0.1% Triton X-100 for 10 min. After staining, all suspensions were analyzed using flow cytometry.

To investigate the expression levels of tumor associated cytokines, IFN-γ and TNF-α in the supernatant of tumor homogenates were then measured using ELISA assay kits. For H&E and immunofluorescence staining, tumors were fixed with 4% paraformaldehyde and sliced to sections. Next, tumor sections were stained with H&E and TUNEL staining kit, respectively. Immunofluorescence staining was performed on a Nikon AlR-SIMe confocal microscope, and H&E-stained slides were scanned using a slide scanner microscope (3DHISTECH).

***In Vivo* L-arg Production and sPD-1 Secretion**

To assess the ability of DC-$EcN_{Arg+sPD\text{-}1}$ to produce L-arg and secrete sPD-1, tumor tissues were weighed and homogenized using a tissue dissociator. The homogenates were then centrifuged (3500 g, 5 min, 4°C) to obtain the supernatant. L-arg levels in the supernatant were detected using a L-Arginine Assay Kit. To detect the secretion of sPD-1, supernatants were separated by SDS-PAGE followed by transfer onto nitrocellulose membranes (Millipore). Membranes were blocked and detected using primary anti-mouse PD-1 (dilution 1:1000). After incubation with

the secondary anti-rabbit conjugated with HRP (Sigma-Aldrich), membranes were washed and reacted with chemiluminescence substrate (Millipore) for visualization.

Tumor Rechallenge and Metastasis Experiments

To investigate the long-term immune memory effects of combinational therapy, a rechallenged tumor model was established. Briefly, B16F10-bearing mice were treated on day 6, 8, and 10 as described previously. Primary tumors were surgically resected on day 13 post-tumor inoculation. Mice were then rechallenged by subcutaneous injection of 1×10^6 B16F10 cells into the contralateral (right) flank. Rechallenged tumor volumes were monitored for 19 days post-rechallenge. To assess the activation of T cell-based antitumor immunity in rechallenged tumor, rechallenged tumors were harvested and stained with FITC-conjugated anti-CD4, APC-conjugated anti-mouse CD3, PE-conjugated anti-mouse CD8, and APC/Cy7-conjugated anti-mouse CD45 for cytometric analysis.

To evaluate the antimetastatic potential of DC-EcN$_{Arg+sPD-1}$, B16F10-bearing mice were treated on day 6, 8, and 10 as described previously. At day 13 post-tumor inoculation, the mice were intravenously challenged with B16F10 cells. To evaluate lung metastasis, lungs were harvested at day 25 post-tumor inoculation, metastatic tumor nodules on the lung surface were counted and analyzed by H&E staining. To analyze memory T cells, spleens were harvested and stained with APC/Cy7-conjugated anti-mouse CD45, APC-conjugated anti-mouse CD3, FITC-conjugated anti-mouse CD4, PE-conjugated anti-mouse CD8, FITC-conjugated anti-mouse CD44, and APC-conjugated anti-mouse CD62L, followed by flow cytometry analysis.

Single-Cell RNA Sequencing Analysis of Tumor Tissues

Single-cell RNA sequencing (scRNA-seq) was conducted on tumor tissues harvested on day 17 from B16F10-bearing mice treated with PBS or DC-EcN$_{Arg+sPD-1}$. Tumors were enzymatically digested to generate single cell suspensions. The cDNA library was prepared using an oligo-dT primer for mRNA capture and was subsequently barcoded through droplet partitioning using the Chromium Single-Cell Controller (10x Genomics) in the NCI-CCR Single-Cell Analysis Tool. Prior to loading, dead cells were removed, and cell viability was confirmed to exceed 90% *via* Trypan blue exclusion. Approximately 10000 viable cells were loaded per channel onto the Chromium Controller. Sequencing was carried out on an Illumina NovaSeq system at the NCI-CCR Sequencing Facility. Raw sequencing data were processed with the following quality control filters: cells were retained only if they expressed between 500 and 6000 genes, exhibited a mitochondrial gene fraction below 15% (a threshold established for mouse cells), and had a predicted doublet rate of less than 5% as determined by the Scrublet algorithm. Bioinformatic

analyses were performed using OmicStudio tools (LC-Bio Technology Co., Ltd., Hangzhou, China; https://www.omicstudio.cn/tool). This platform was used for cell type identification and visualization, differential gene expression analysis and pathway enrichment analysis. Single-cell RNA-seq raw data have been deposited into the NCBI Sequence Read Archive (SRA) under BioProject accession number PRJNA1334278 (https://www.ncbi.nlm.nih.gov/bioproject/PRJNA1334278) and will be made publicly available upon publication.

Statistical Analysis

All data were represented as mean ± standard deviation (mean ± s.d.) from more than three independent experiments ($n \geq 3$). Statistical comparisons were performed using Student's *t*-test for two group comparisons, and one-way analysis of variance (ANOVA) with Tukey's test or two-way ANOVA with Sidak's test for multiple comparisons where appropriate. All statistical analyses were obtained using GraphPad Prism (Prism 10.1.2). Flow cytometric data were analyzed by FlowJo software (v10.8.1). The significant levels are shown as ns > 0.05, *$p < 0.05$, **$P < 0.01$, ***$P < 0.001$, and ****$P < 0.0001$ as indicated.

References

1. Y. Xing, J. Li, L. Wang, et al., "A bifunctional lysosome-targeting chimera nanoplatform for tumor-selective protein degradation and enhanced cancer immunotherapy," *Advanced Materials* **37** (2025): e2417942, https://doi.org/10.1002/adma.202417942.
2. Z. Zhang, Z. Pan, Q. Li, Q. Huang, L. Shi, and Y. Liu, "Rational design of ICD-inducing nanoparticles for cancer immunotherapy," *Science Advances* **10** (2024): eadk0716, https://doi.org/10.1126/sciadv.adk0716.
3. Y. Liu, J. Du, M. Yan, et al., "Biomimetic enzyme nanocomplexes and their use as antidotes and preventive measures for alcohol intoxication," *Nature Nanotechnology* **8** (2013): 187-192, https://doi.org/10.1038/nnano.2012.264.

Supplementary Figures and Tables

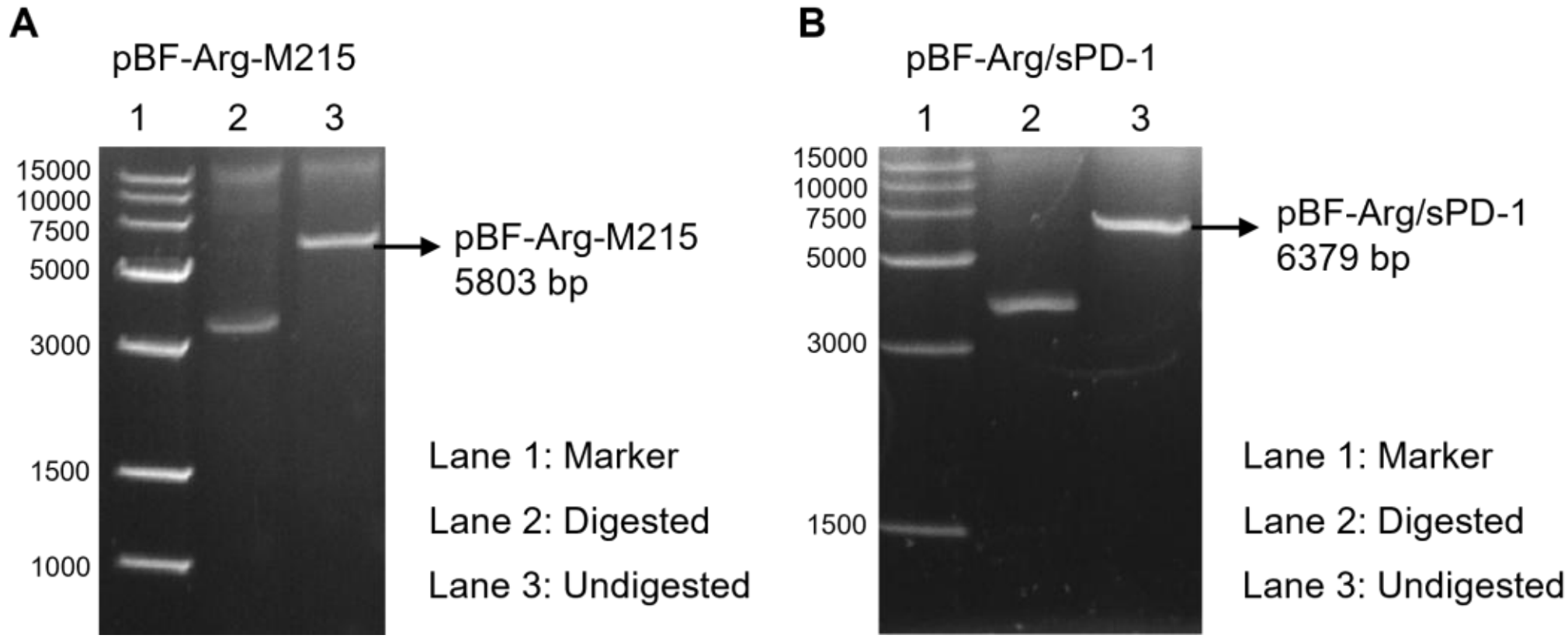

Figure S1. Restriction enzyme digestion maps of plasmids pBF-Arg-M215 (A) and pBF-Arg/sPD-1 (B), respectively.

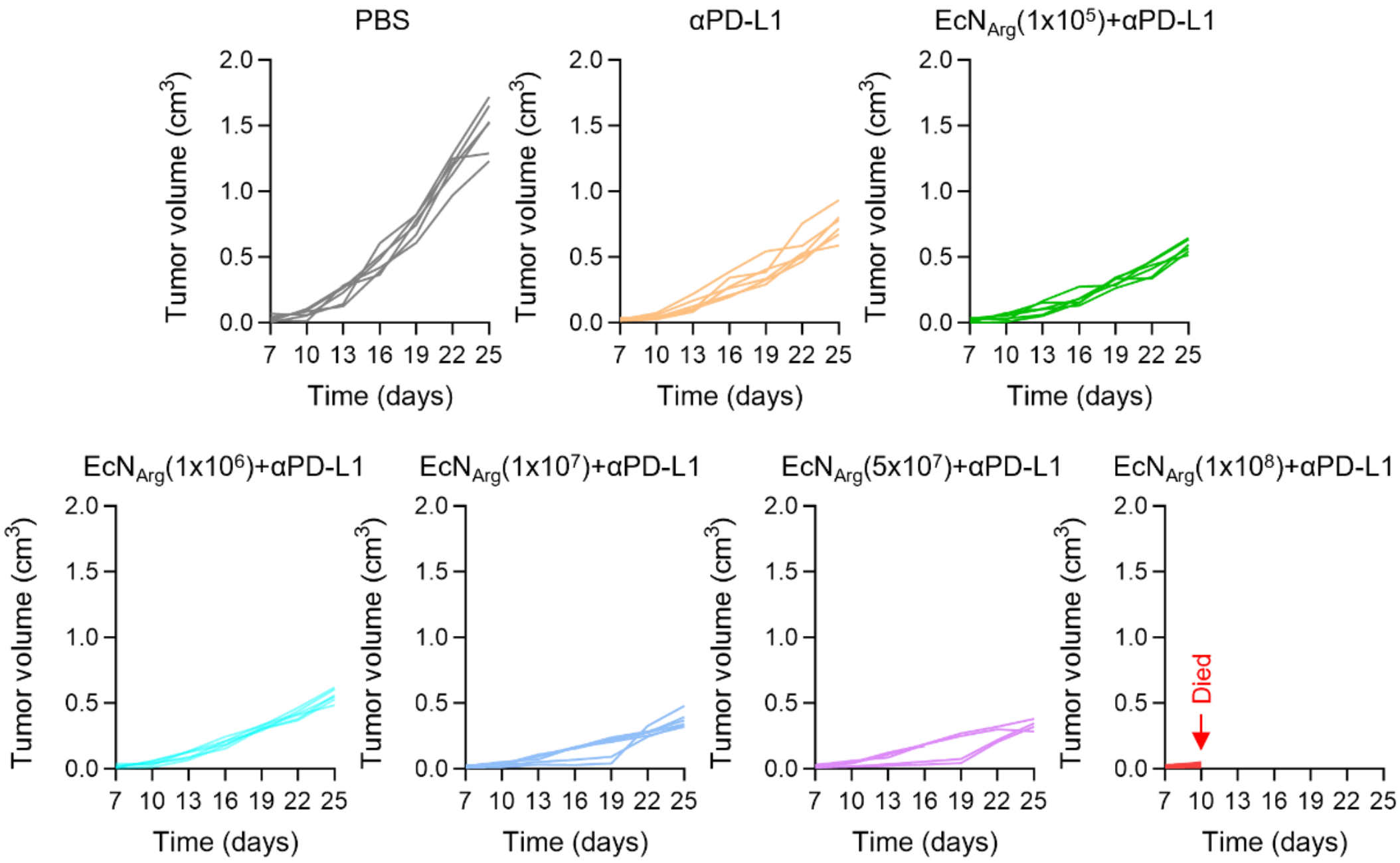

Figure S2. Individual tumor growth curves after different treatments (n = 6).

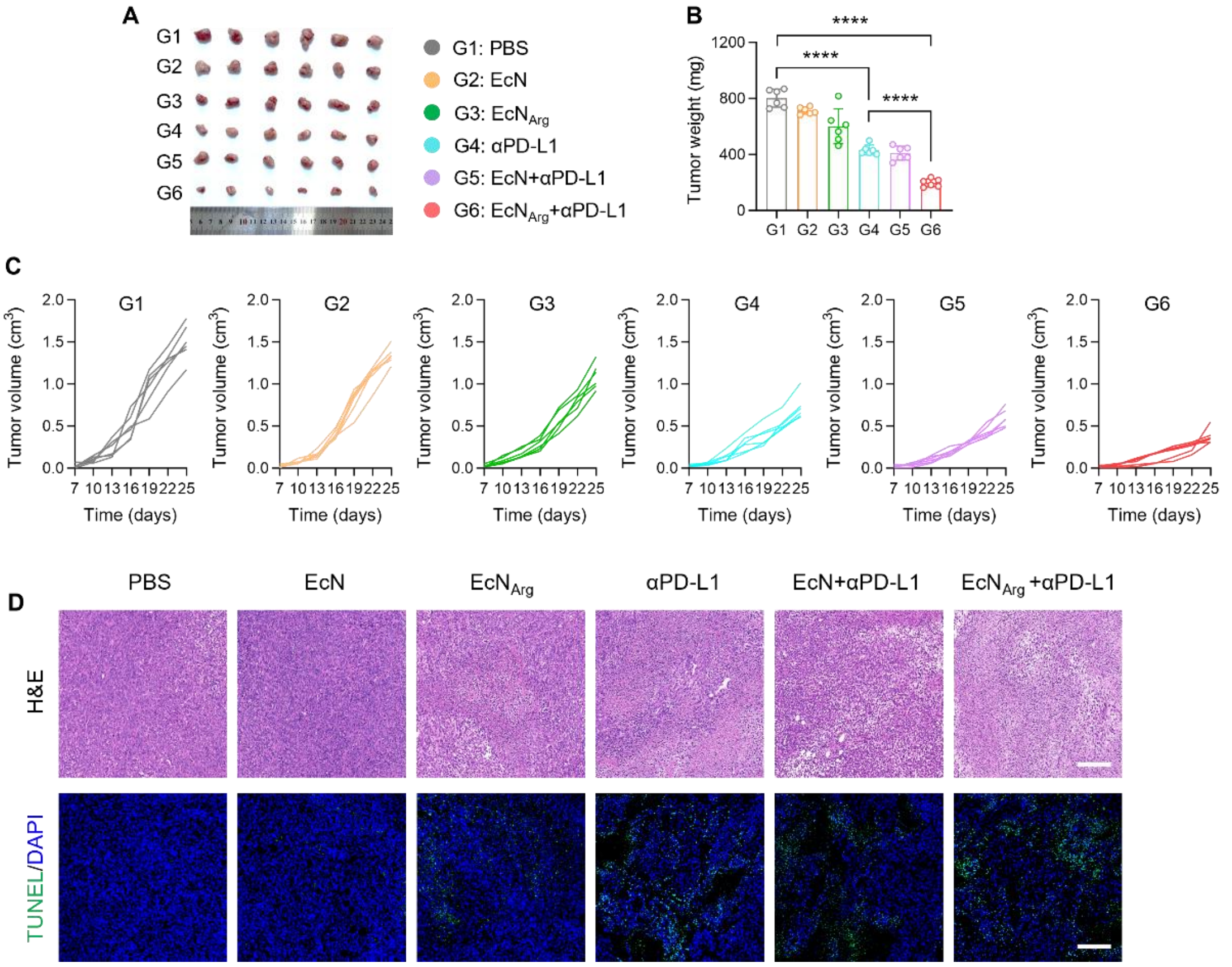

Figure S3. (A) Individual tumor growth curves after different treatments. (B) Tumor images after different treatments. (C) Tumor weight after different treatments. Data in (C) are presented as means ± s.d. from six independent experiments (n = 6). (D) H&E and TUNEL staining of tumor sections from the mice after different treatments; Scale bars: 200 μm. Statistical analysis in (C) was performed using one-way ANOVA followed by Tukey's test with multiple comparisons. The significant level is as ****$P < 0.0001$.

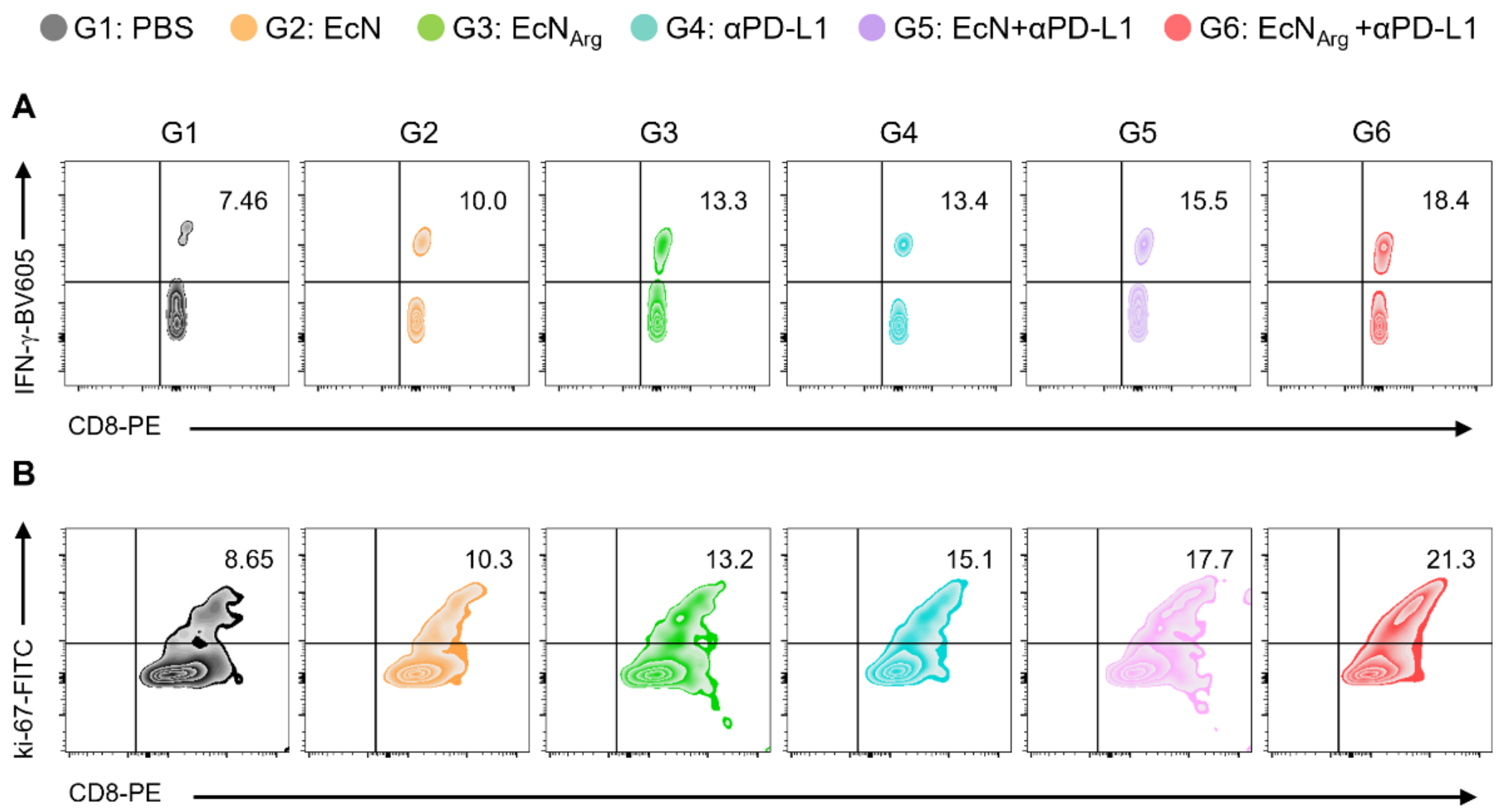

Figure S4. Representative flow cytometric analysis of tumor-infiltrating IFN-γ^+ $CD8^+$ (A) and Ki-67^+ $CD8^+$ (B) T cells after different treatments.

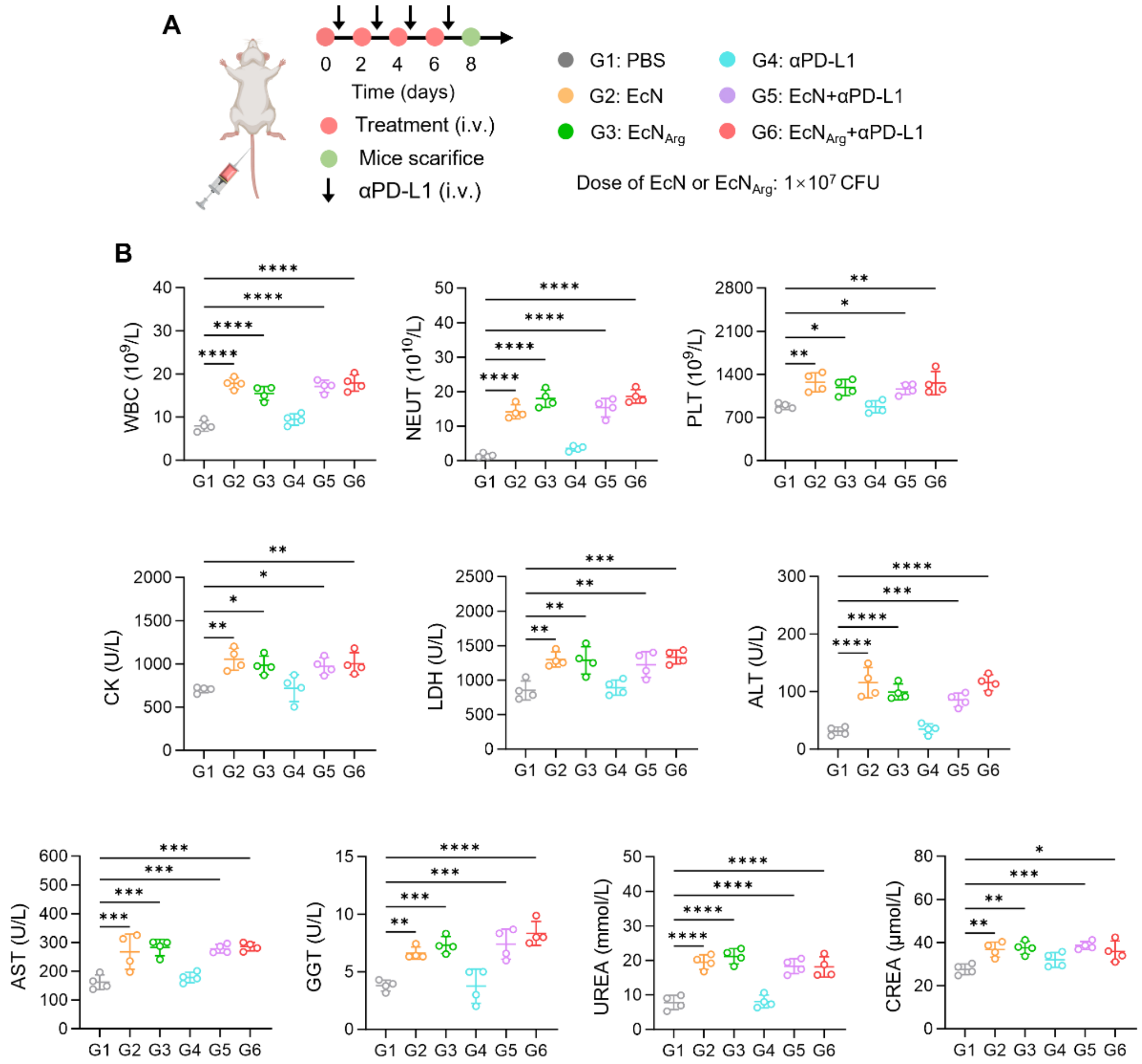

Figure S5. (A) Schematic illustration for evaluating the biosafety of EcN_{Arg}. (B) Blood biochemical and routine blood tests after different treatments, including white blood cell (WBC), neutrophil (NEUT), platelet (PLT), creatine kinase (CK), dehydrogenase (LDH), alanine aminotransferase (ALT), aspartate transaminase (AST), γ-glutamyl transferase (GGT), urea (UREA), and creatinine (CREA). Data in (B) are presented as means ± s.d. from four independent experiments (*n* = 4). Statistical analysis in (B) was performed using one-way ANOVA followed by Tukey's test with multiple comparisons. The significant levels are shown as $^*P < 0.05$, $^{**}P < 0.01$, $^{***}P < 0.001$, and $^{****}P < 0.0001$.

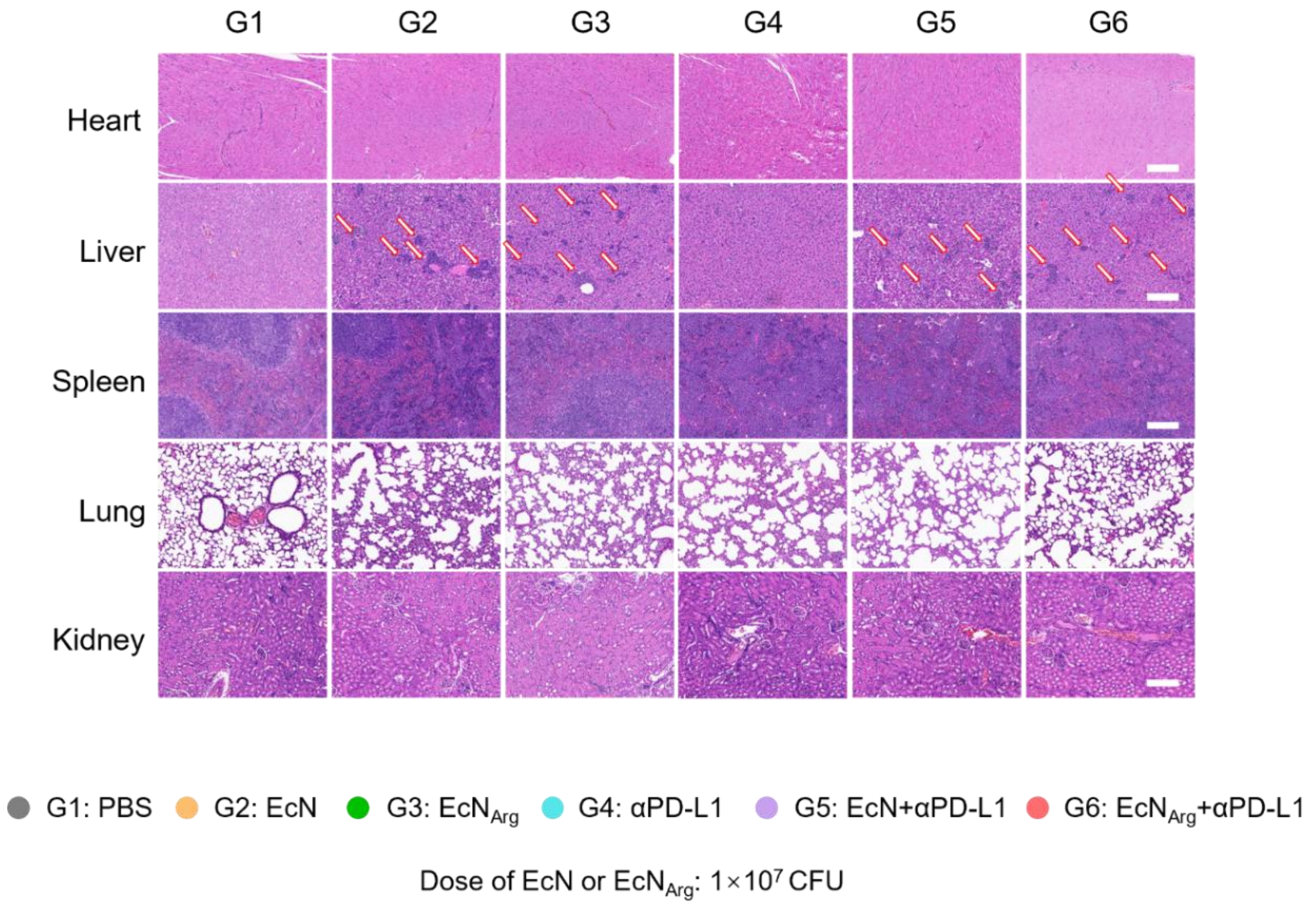

Figure S6. Representative H&E staining images of major organs of mice treated with various formulations; Scale bars: 200 μm.

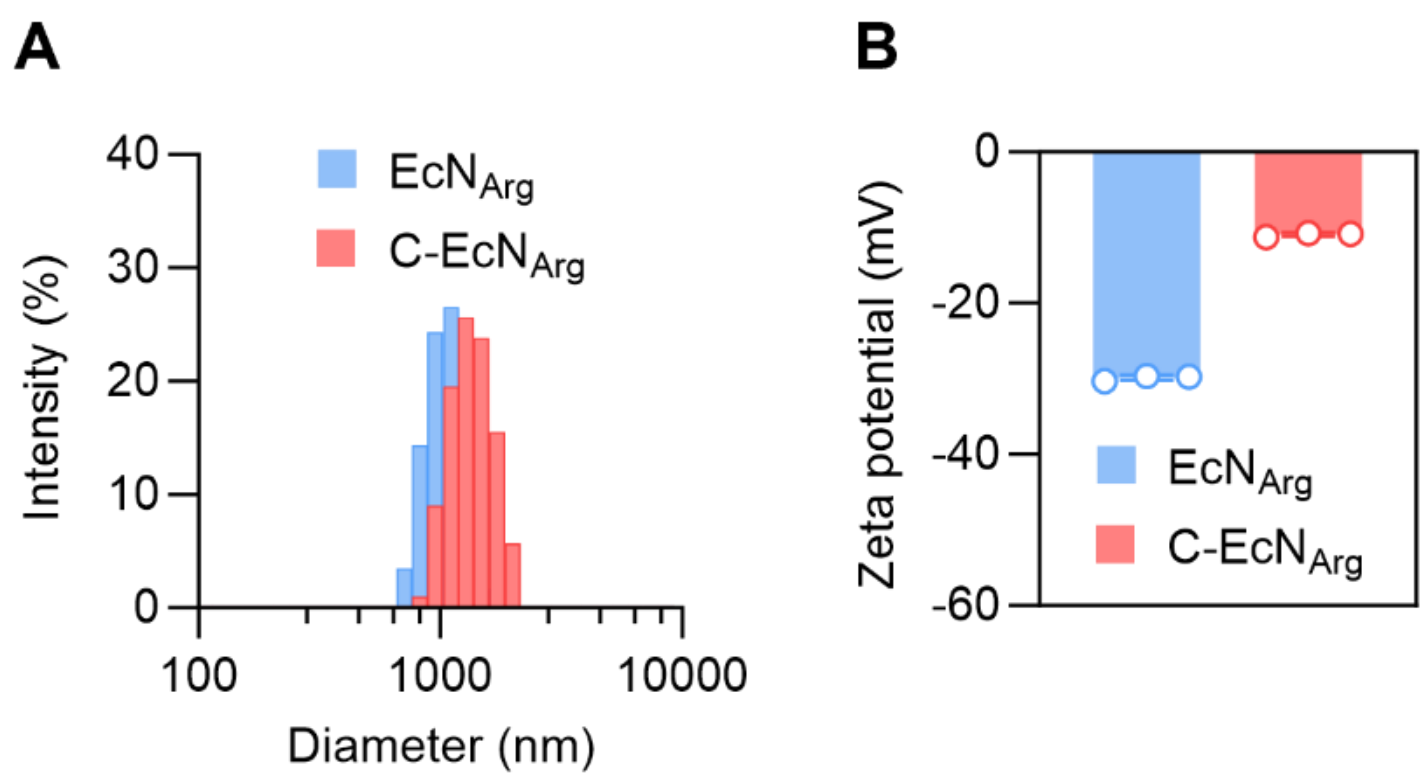

Figure S7. (A) Size distribution of EcN$_{Arg}$ and C-EcN$_{Arg}$. (B) Zeta potential of EcN$_{Arg}$ and C-EcN$_{Arg}$. Data in (B) are presented as means ± s.d. from three independent experiments (n = 3).

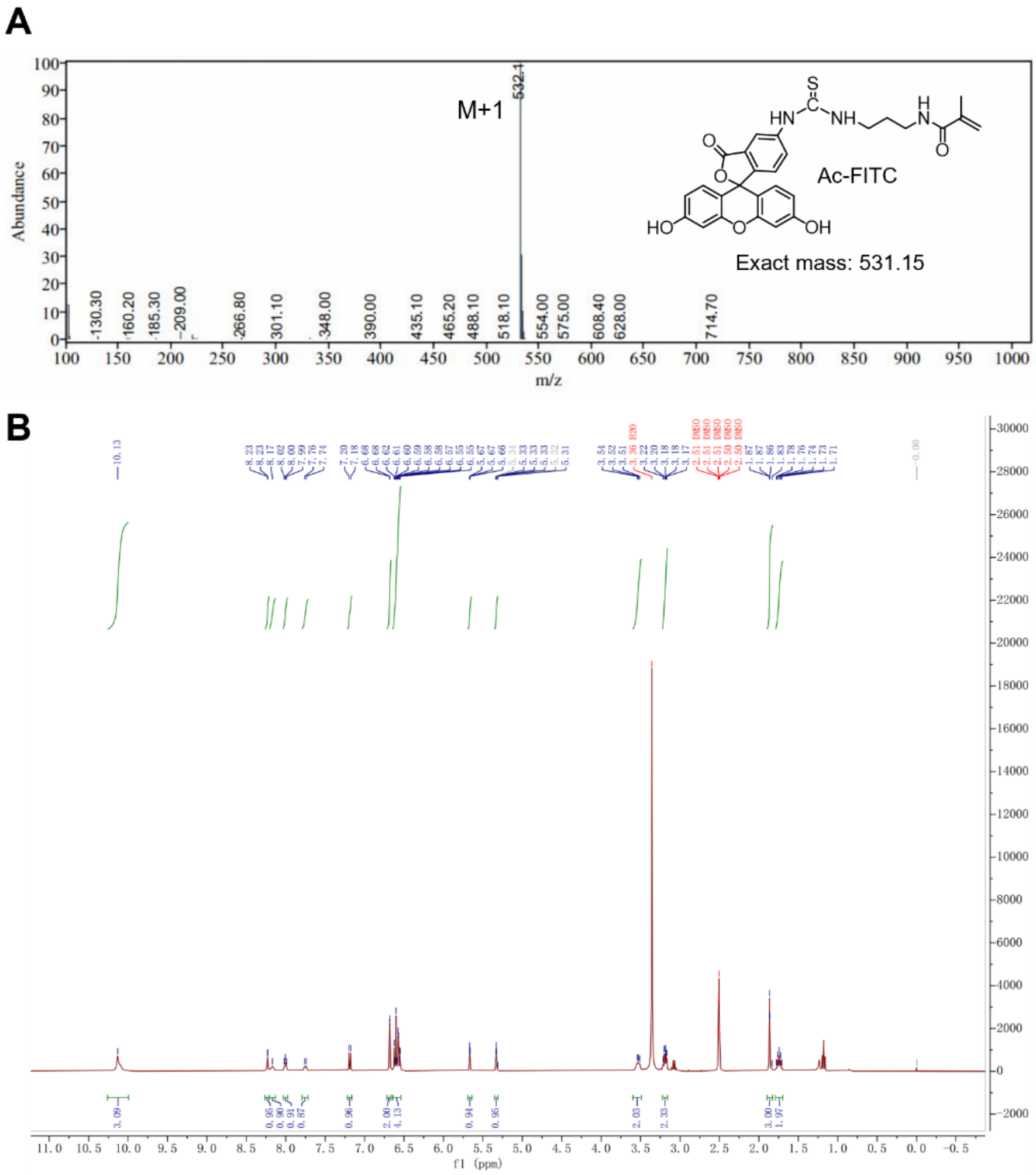

Figure S8. Electrospray ionization mass spectrometry (ESI-MS) analysis (A) and ^{1}H NMR spectrum (B) of Ac-FITC in D_2O, 400 MHz, 25˚C.

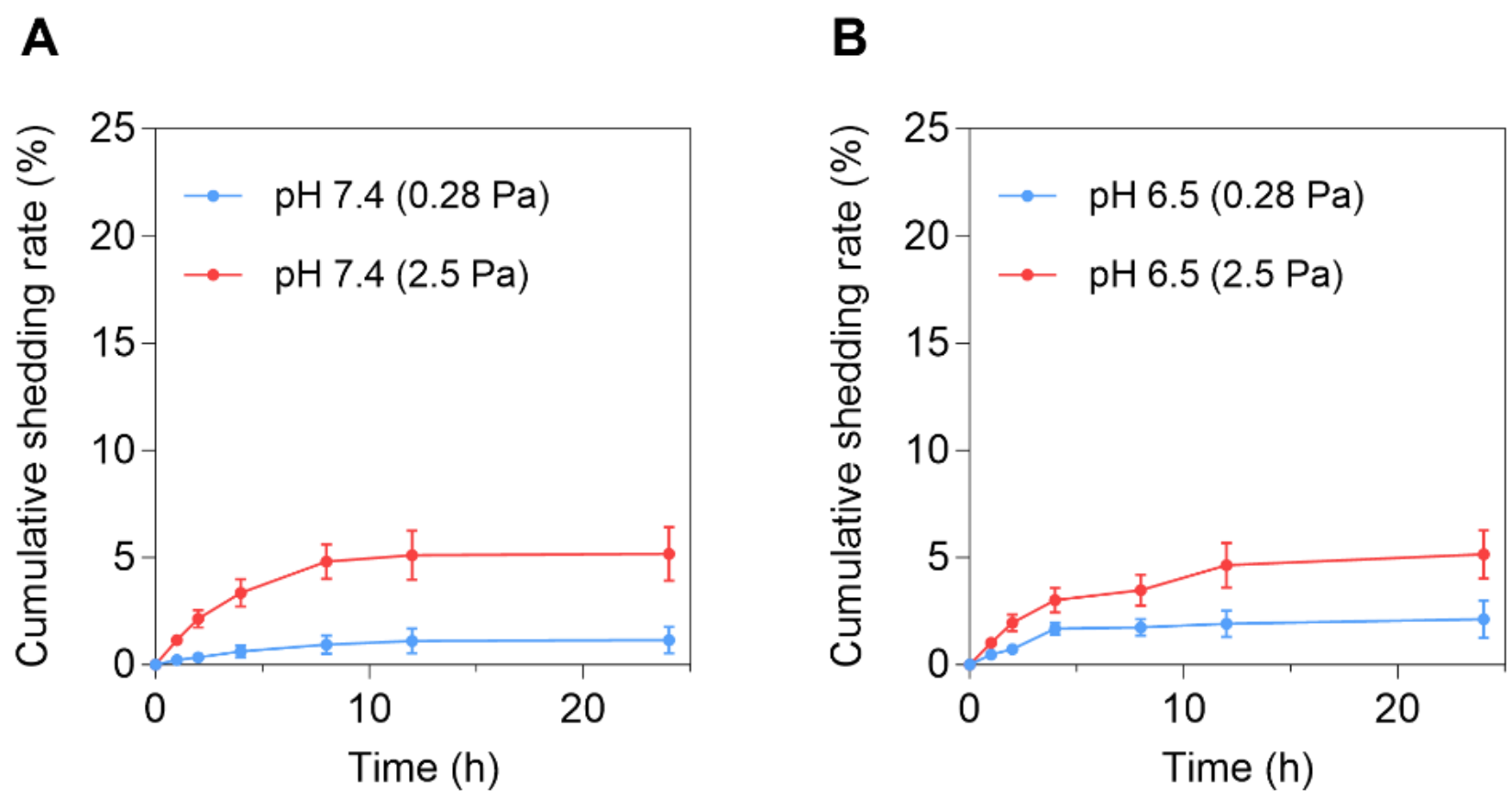

Figure S9. Cumulative shedding rates of surface polymer shell from C-EcN$_{Arg}$ capsules over 24 hours after incubation in simulated dynamic fluid environments. (A) Cumulative shedding rates in simulated blood circulation medium at pH 7.4 under shear stress of venous system (0.28 Pa) and resting large arteries (2.5 Pa). (B) Cumulative shedding rates in simulated blood circulation medium at pH 6.5 under shear stress of venous system (0.28 Pa) and resting large arteries (2.5 Pa). Data are presented as mean ± s.d. based on three independent experimental replicates (n = 3).

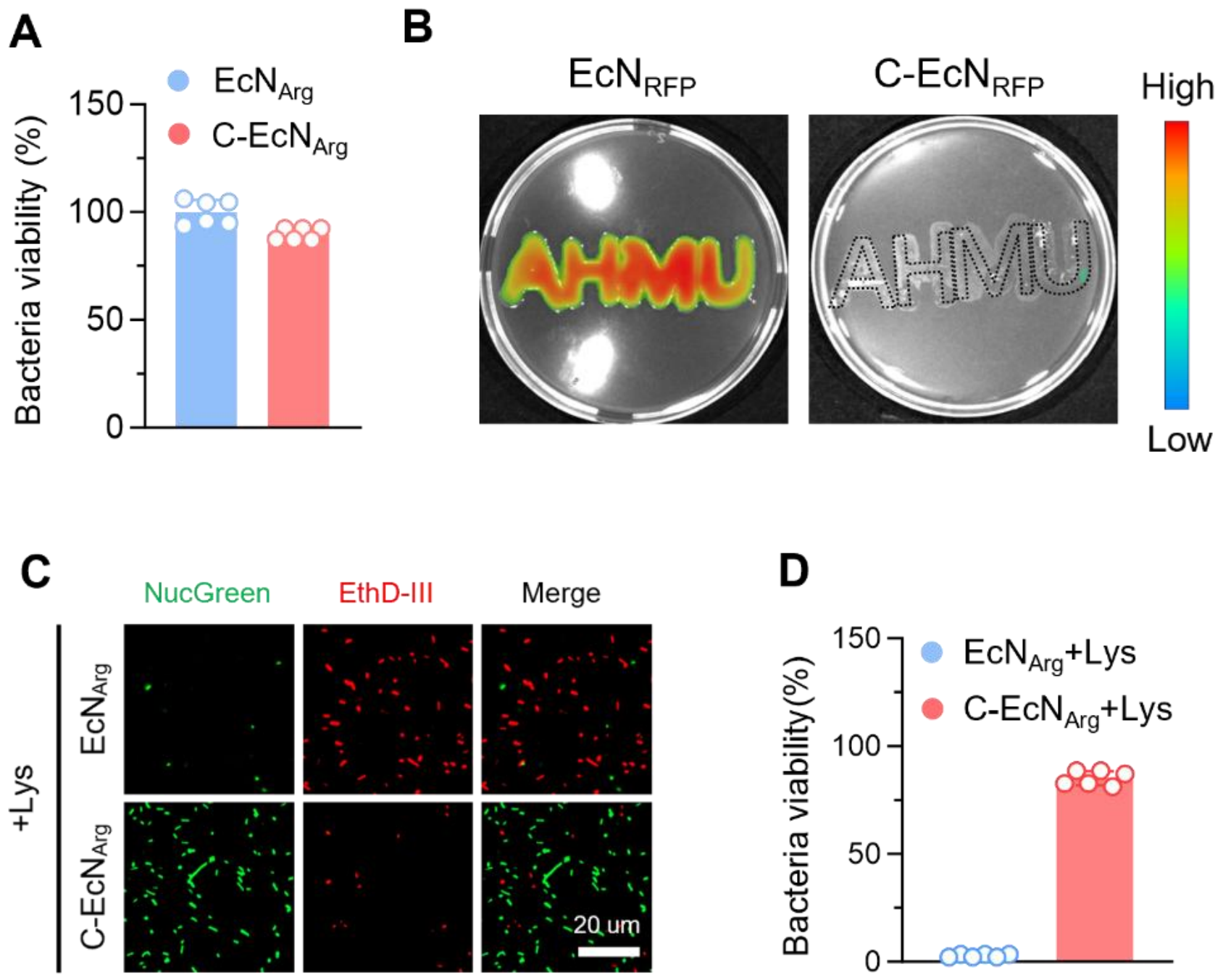

Figure S10. (A) Bacterial viability post encapsulation (C-EcN$_{Arg}$). (B) Fluorescence photographs of LB agar plates spread with EcN$_{RFP}$ and C-EcN$_{RFP}$ after 10 h incubation. (C) NucGreen (living bacteria, green) and EthD-III (dead bacteria, red) staining images of EcN$_{Arg}$ and C-EcN$_{Arg}$ in the presence of lysozyme (50 μg mL^{-1}); Scale bar: 20 μm. (D) Bacterial viability of EcN$_{Arg}$ and C-EcN$_{Arg}$ in the presence of lysozyme (50 μg mL^{-1}). Data in (A) and (B) are presented as means ± s.d. from six independent experiments (n = 6).

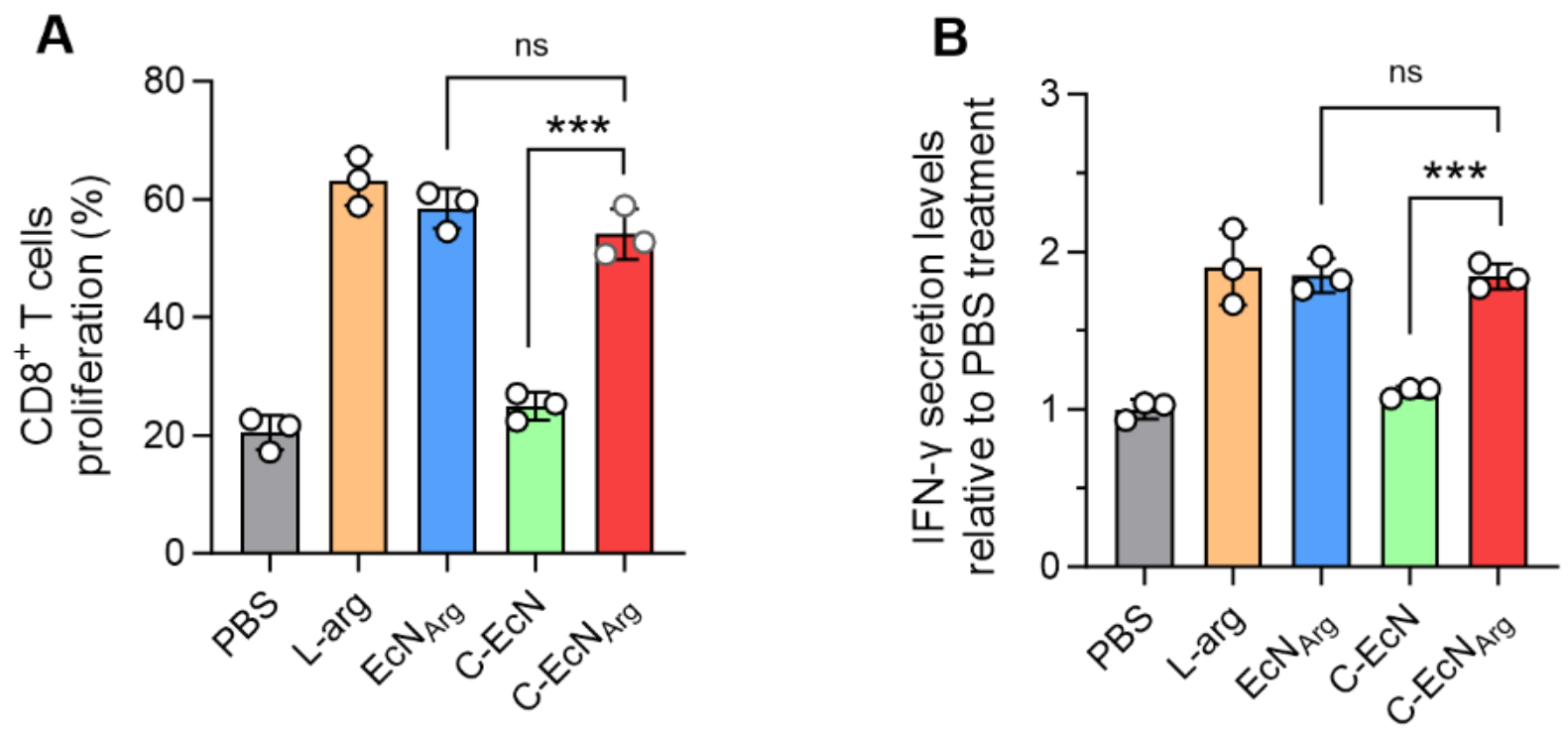

Figure S11. (A) Quantitative analysis of T cells proliferation after different treatments. (B) IFN-γ secretion levels of T cells after different treatments. Data are presented as mean ± s.d. based on three independent experimental replicates ($n = 3$). Statistical analysis was performed using one-way ANOVA followed by Tukey's test with multiple comparisons. The significant levels are shown as ns > 0.05 and ***$P < 0.001$.

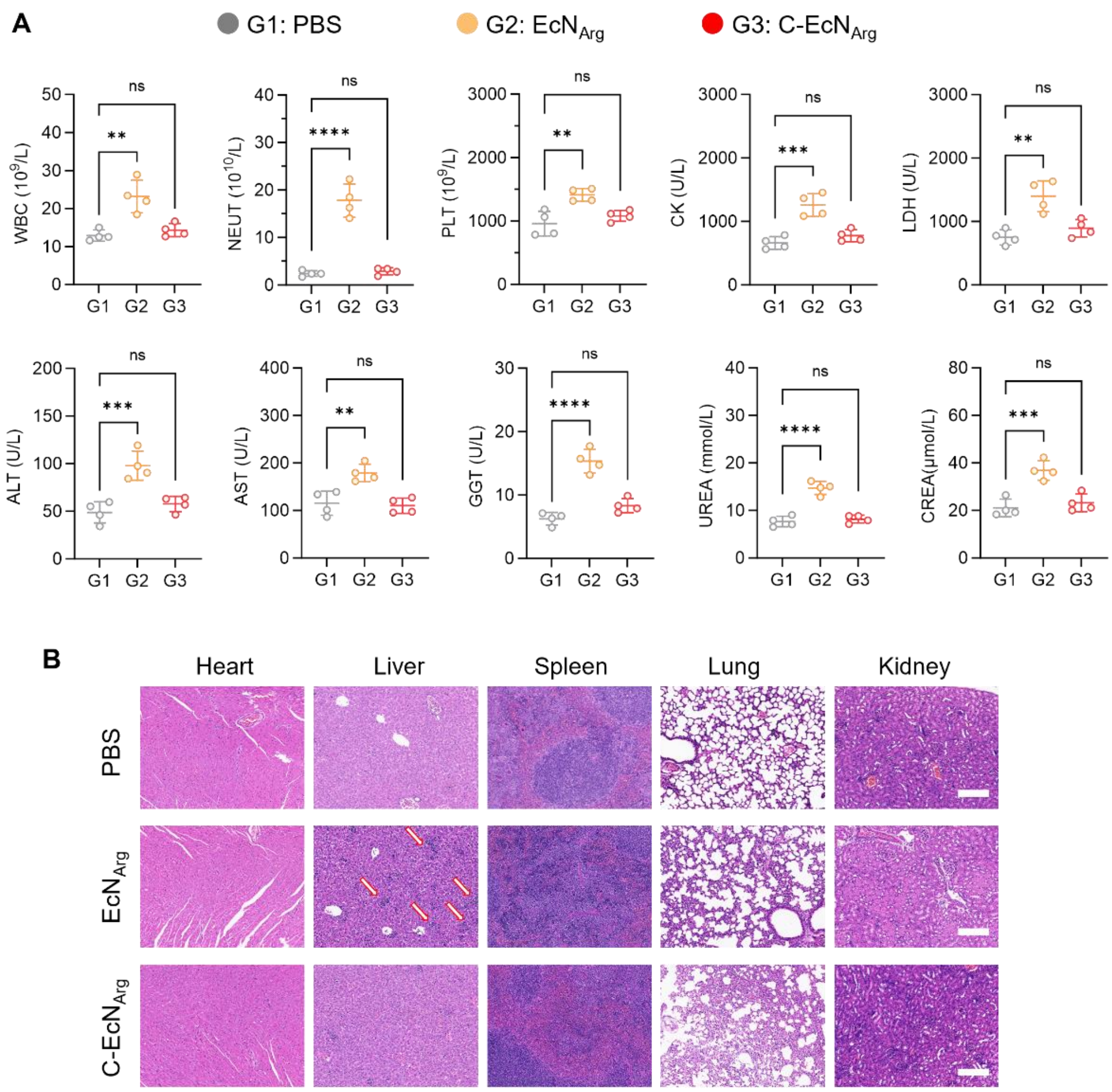

Figure S12. (A) Blood biochemical and routine blood tests after different treatments, including white blood cell (WBC), neutrophil (NEUT), platelet (PLT), creatine kinase (CK), dehydrogenase (LDH), alanine aminotransferase (ALT), aspartate transaminase (AST), γ-glutamyl transferase (GGT), urea (UREA), and creatinine (CREA). Data are presented as means ± s.d. from four independent experiments (n = 4). (B) Representative H&E staining images of major organs (heart, liver, spleen, lung, and kidney) of mice treated with various formulations; Scale bar: 200 μm. Statistical analysis in (A) was performed using one-way ANOVA followed by Tukey's test with multiple comparisons. The significant levels are shown as ns > 0.05, $^{**}P < 0.01$, $^{***}P < 0.001$, and $^{****}P < 0.0001$.

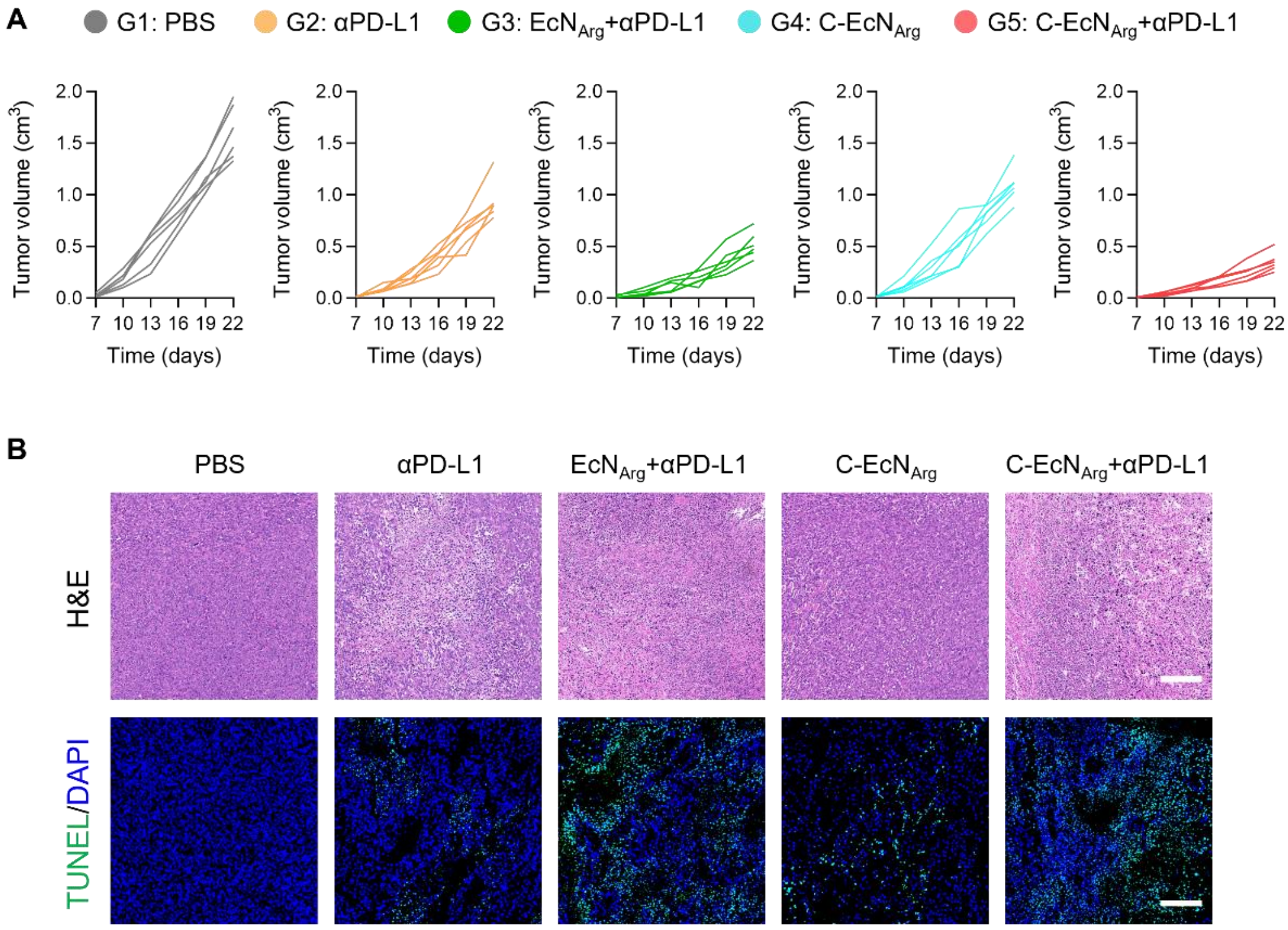

Figure S13. (A) Individual tumor growth curves after different treatments (n = 6). (B) H&E and TUNEL staining of tumor sections from the mice after different treatments; Scale bars: 200 μm.

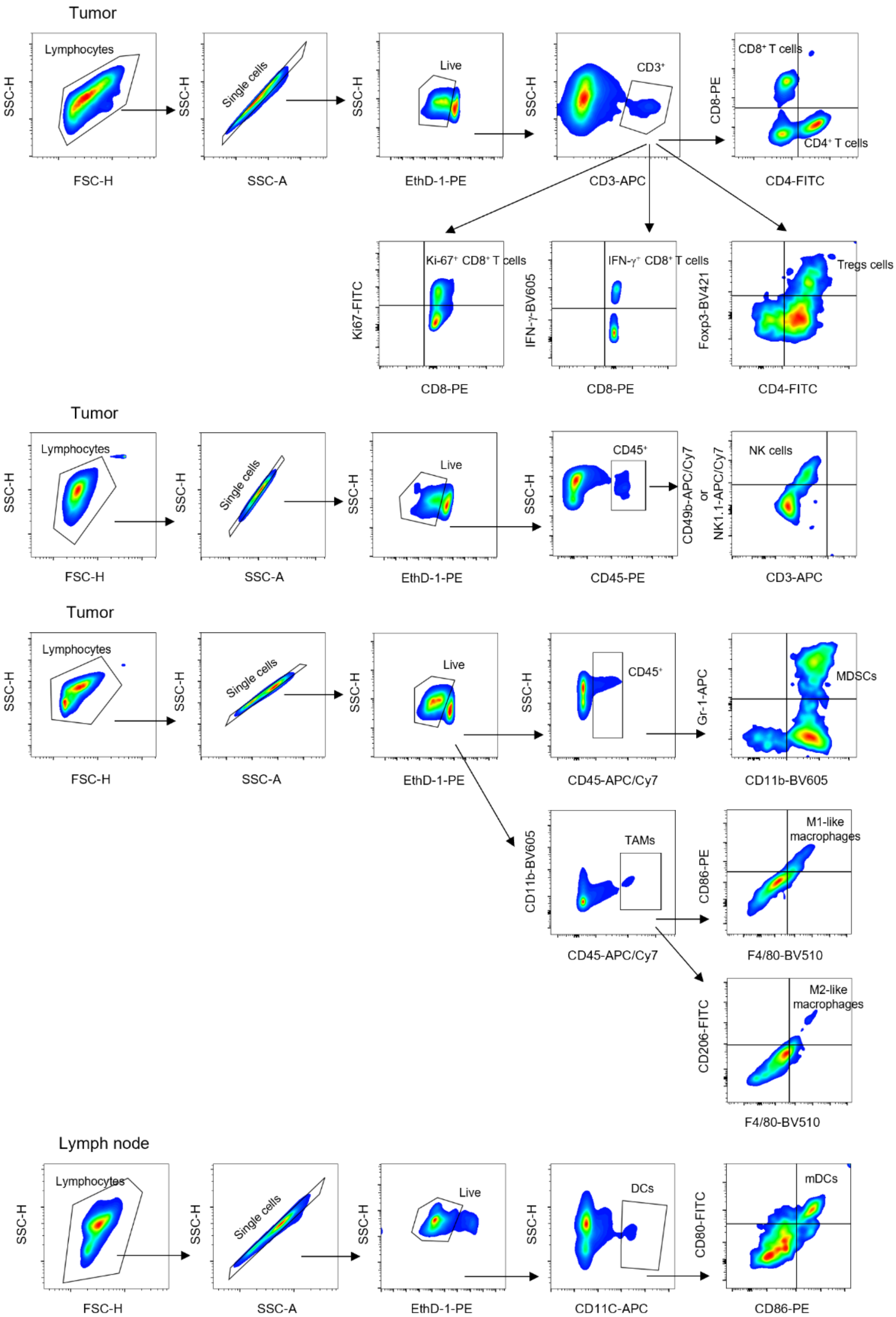

Figure S14. Flow cytometric gating strategies for the analysis of CD8$^+$ T cells, NK cells, MDSCs, tumor-associated macrophages, and Tregs in tumors, and DCs in LNs after treatments.

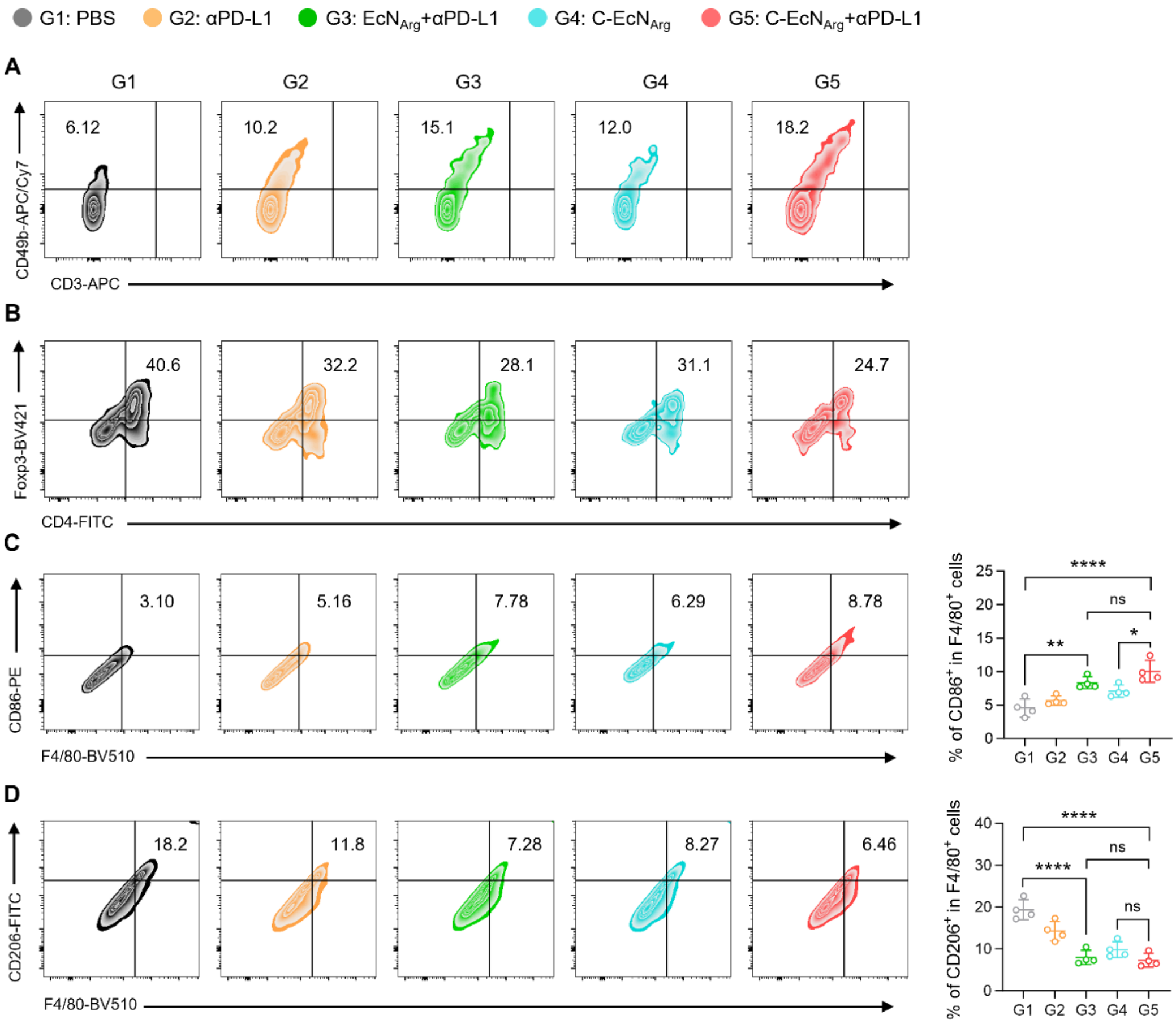

Figure S15. (A, B) Representative flow cytometric analysis of tumor-infiltrating NK cells (A) and Tregs (B) after different treatments. (C, D) Flow cytometric analysis of tumor-infiltrating M1-like (C) and M2-like (D) macrophages after different treatments. Data in (C) and (D) are presented as means ± s.d. from four independent experiments (n = 4). Statistical analysis was performed using one-way ANOVA followed by Tukey's test with multiple comparisons. The significant levels are shown as ns > 0.05, *P < 0.05, **P < 0.01, and ****P < 0.0001.

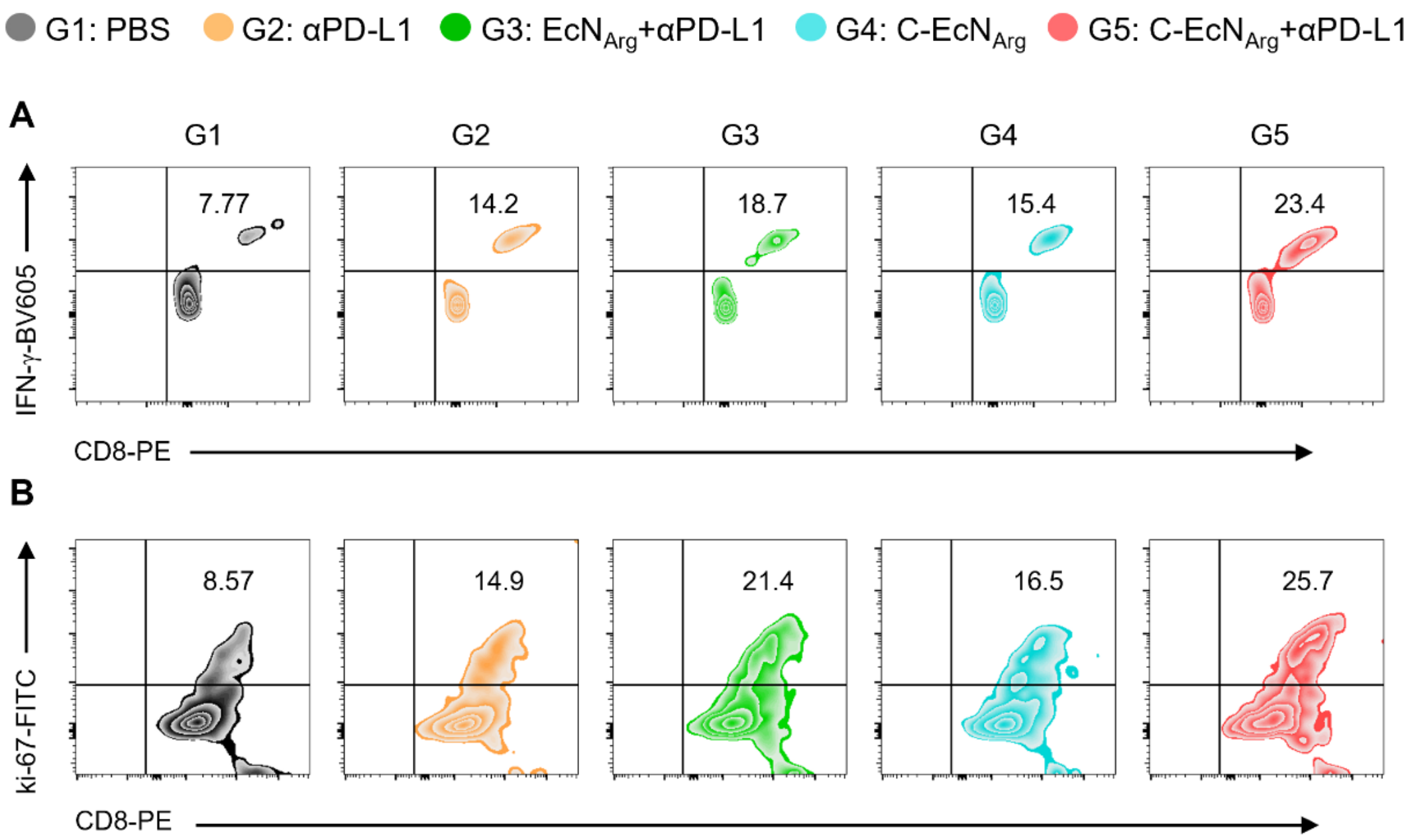

Figure S16. Representative flow cytometric analysis of tumor-infiltrating IFN-γ^{+} CD8^{+} (A) and Ki-67^{+} CD8^{+} (B) T lymphocytes.

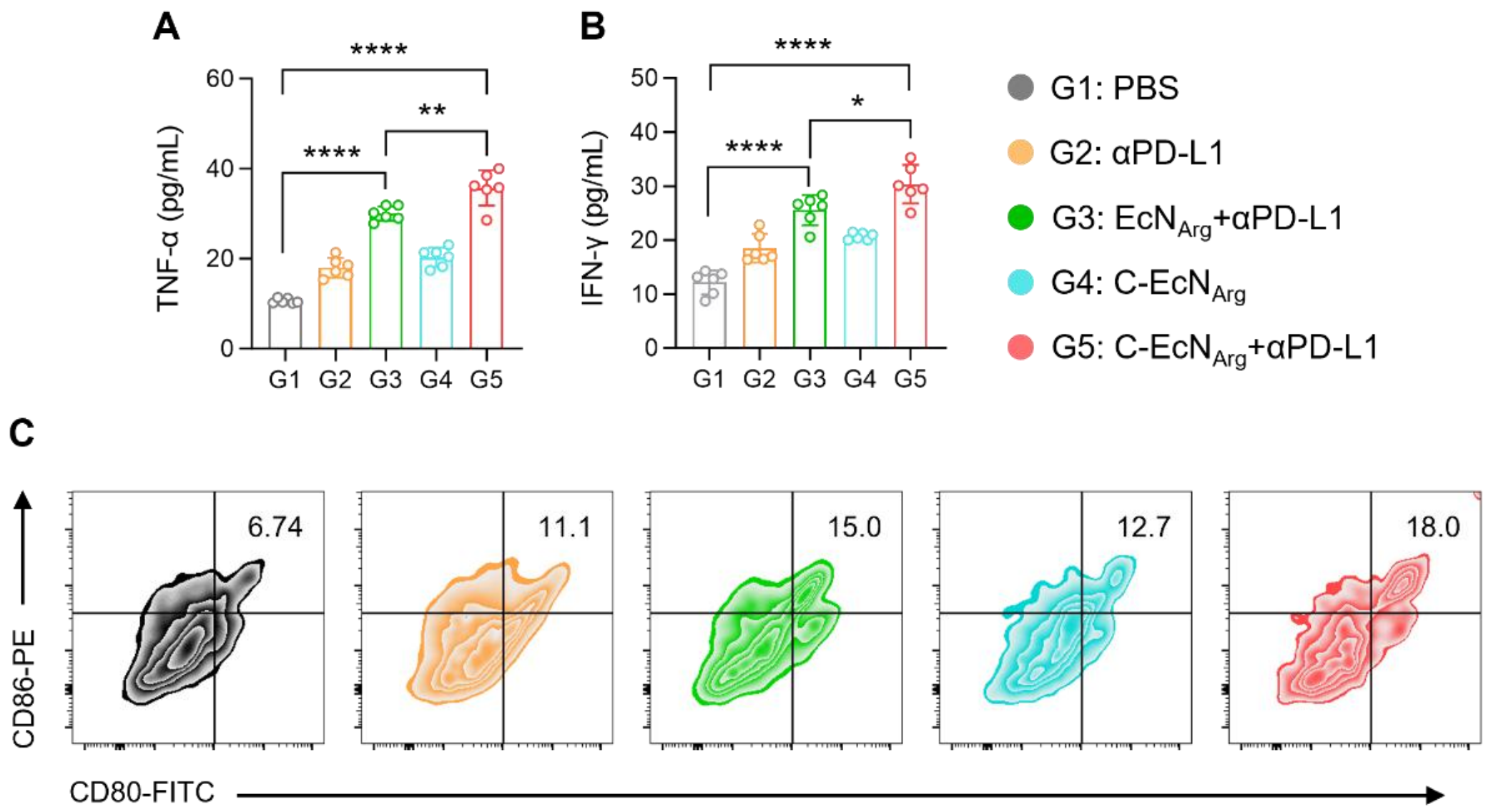

Figure S17. (A, B) Relative expression levels of TNF-α (A) and IFN-γ (B) in tumor tissues after different treatments. (C) Representative flow cytometric analysis of tumor-infiltrating DCs in LNs after different treatments. Data in (A) and (B) are presented as mean ± s.d. from six biologically independent samples (n = 6). Statistical analyses in (A) and (B) were performed using one-way ANOVA followed by Tukey's test with multiple comparisons. The significant levels are shown as *P < 0.05, **P < 0.01, and ****P < 0.0001.

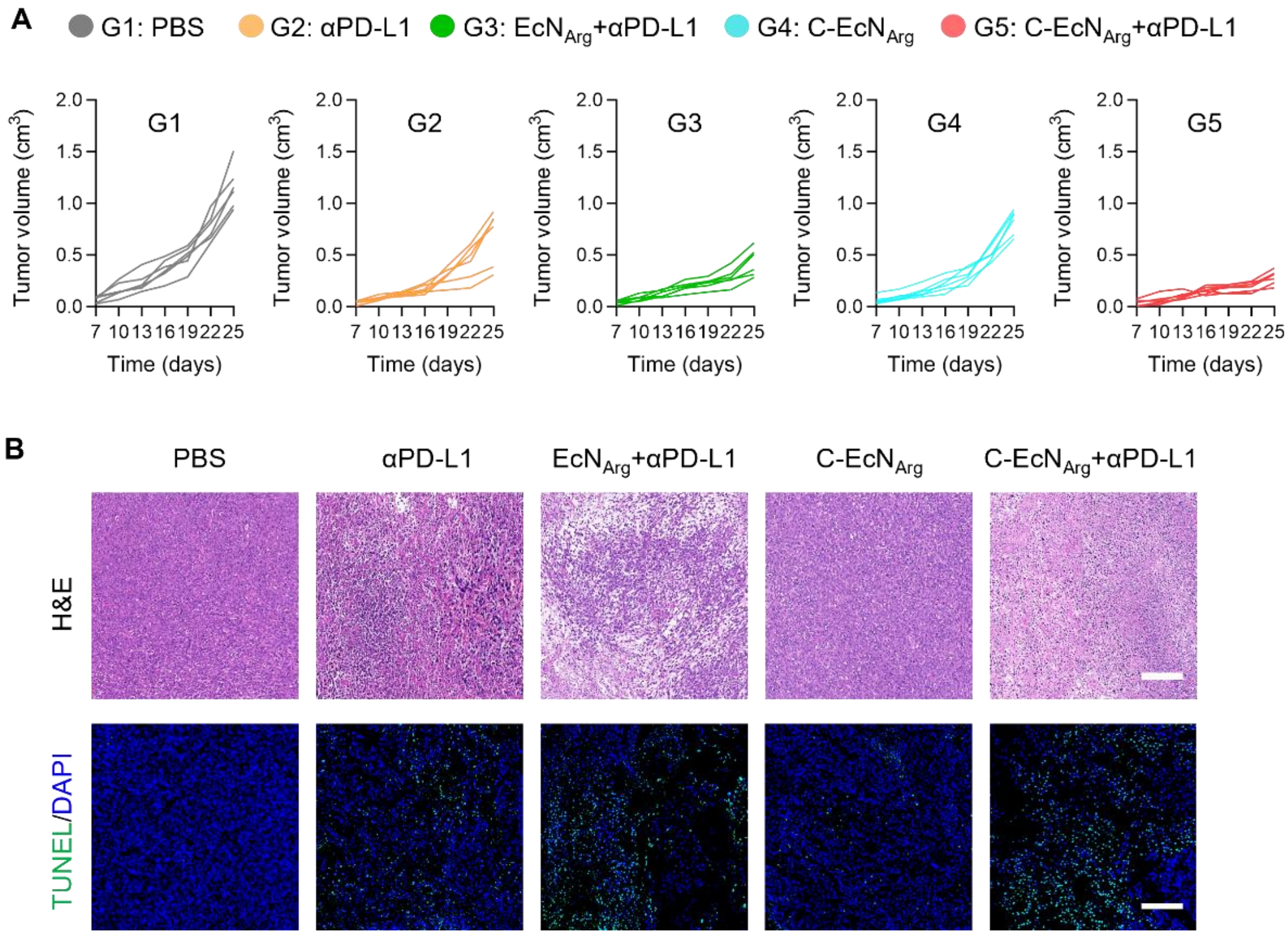

Figure S18. (A) Individual tumor growth curves after different treatments (n = 6). (B) H&E and TUNEL staining of tumor sections from the mice after different treatments; Scale bars: 200 µm.

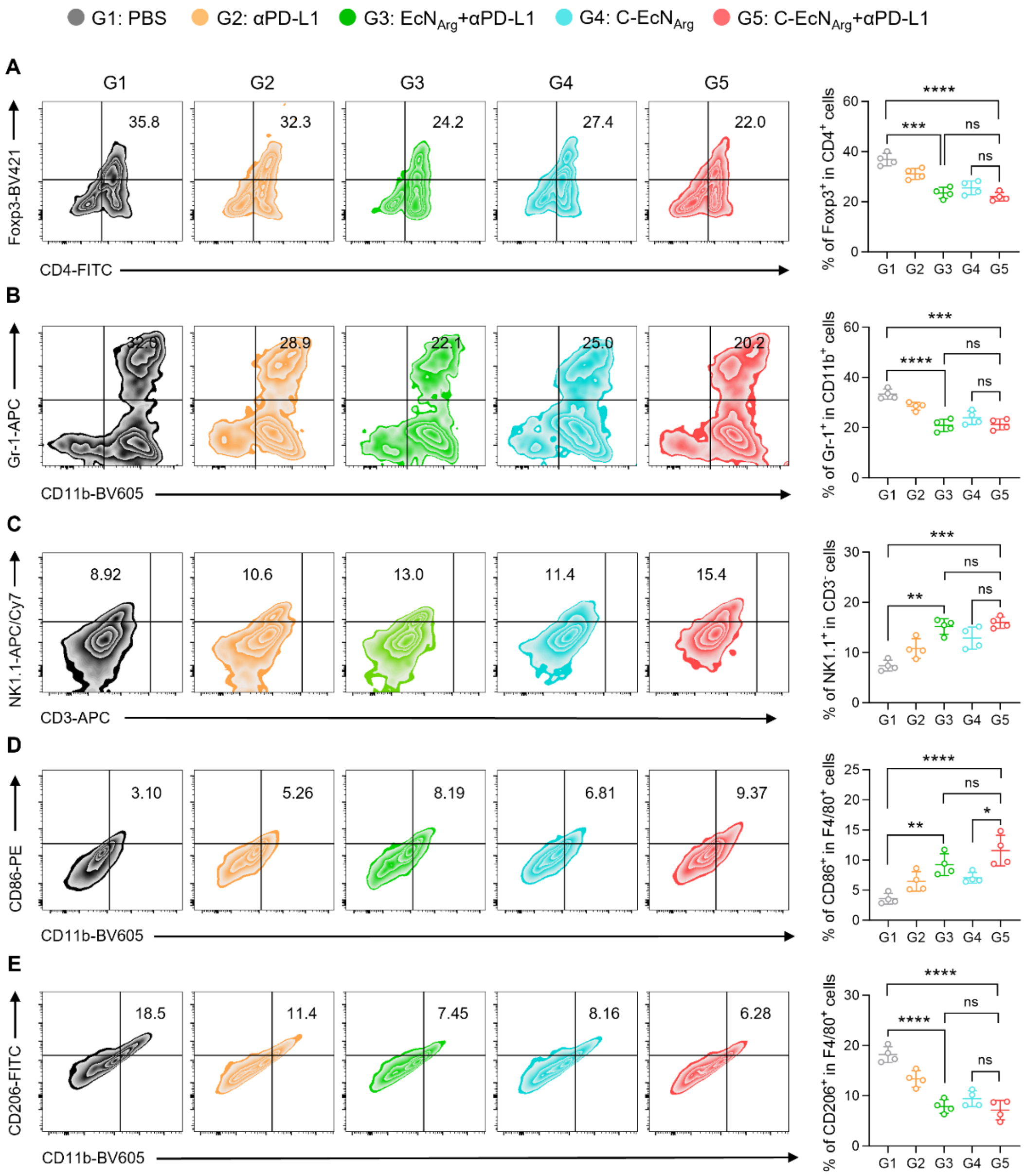

Figure S19. Flow cytometric analysis of the tumor infiltration of Tregs (A), MDSCs (B), NK cells (C), M1-like macrophages (D), and M2-like macrophages (E) after different treatments. Data are presented as means ± s.d. from four independent experiments (n = 4). Statistical analysis was performed using one-way ANOVA followed by Tukey's test with multiple comparisons. The significant levels are shown as ns > 0.05, ***$P < 0.001$, and ****$P < 0.0001$.

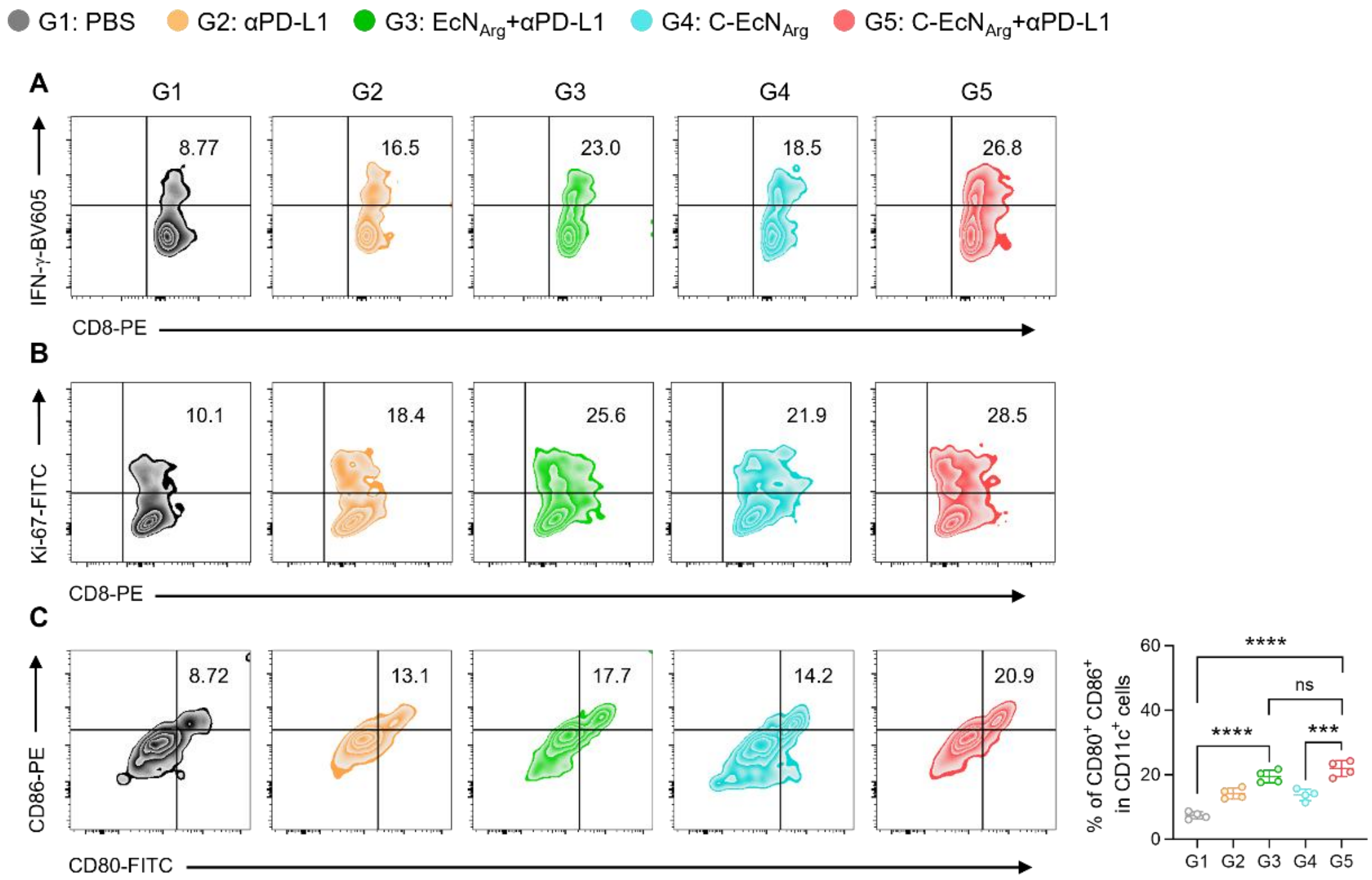

Figure S20. (A, B) Representative flow cytometric analysis of the tumor infiltration of IFN-γ^+ $CD8^+$ (A) and Ki-67$^+$ $CD8^+$ T lymphocytes (B) in tumor tissues after different treatments. (C) Flow cytometric analysis of DCs in LNs after different treatments. Data are presented as means ± s.d. from four independent experiments (n = 4). Statistical analysis was performed using one-way ANOVA followed by Tukey's test with multiple comparisons. The significant levels are shown as ns > 0.05, ***P < 0.001, and ****P < 0.0001.

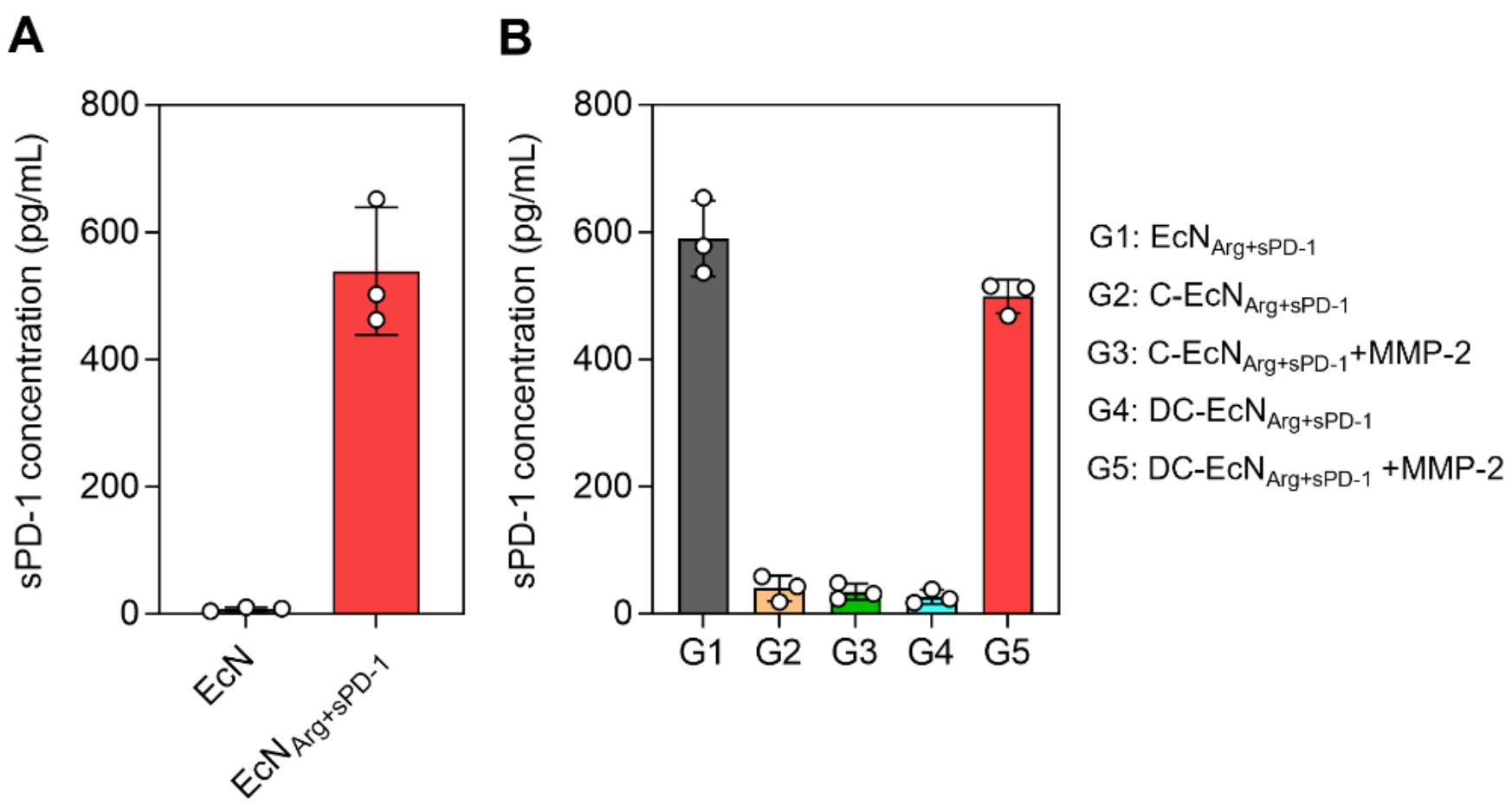

Figure S21. (A) sPD-1 expression in the supernatant of EcN and EcN$_{Arg+sPD-1}$. (B) Western blot sPD-1 expression in the supernatant of C-EcN$_{Arg+sPD-1}$ and DC-EcN$_{Arg+sPD-1}$ in the absence or presence of MMP-2 (10 nM). All sPD-1 levels were detected using a commercial mouse sPD-1 ELISA kit. Data are presented as mean ± s.d. derived from three independent biological replicates (*n* = 3).

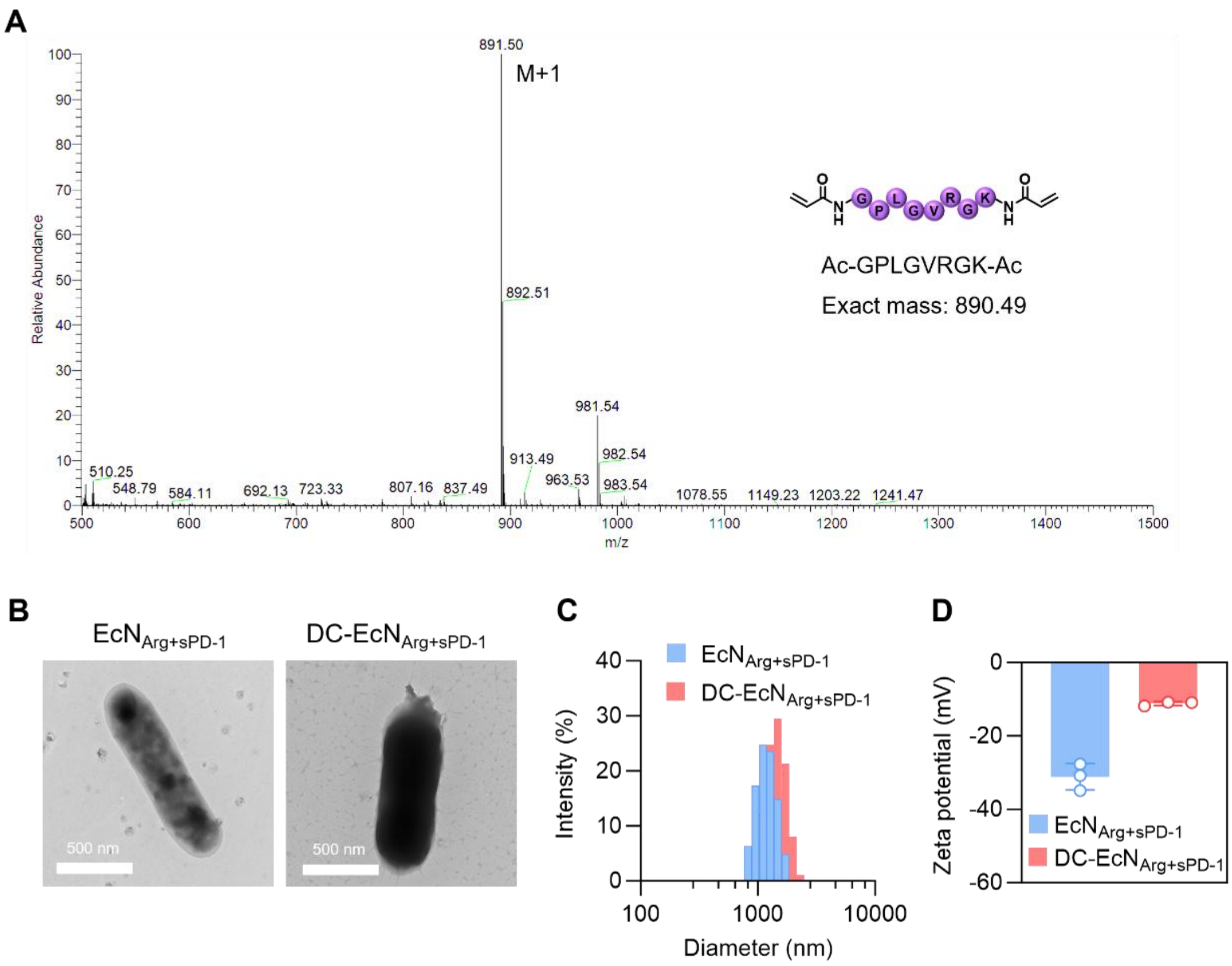

Figure S22. (A) ESI-MS analysis of MMP-2-cleavable peptide crosslinker (Ac-GPLGVRGK-Ac). (B) TEM images of EcN$_{Arg+sPD-1}$ and DC-EcN$_{Arg+sPD-1}$; Scale bars: 500 nm. (C, D) Size distribution (C) and zeta potential (D) of EcN$_{Arg+sPD-1}$ and DC-EcN$_{Arg+sPD-1}$. Data in (D) are presented as means ± s.d. from three independent experiments (*n* = 3).

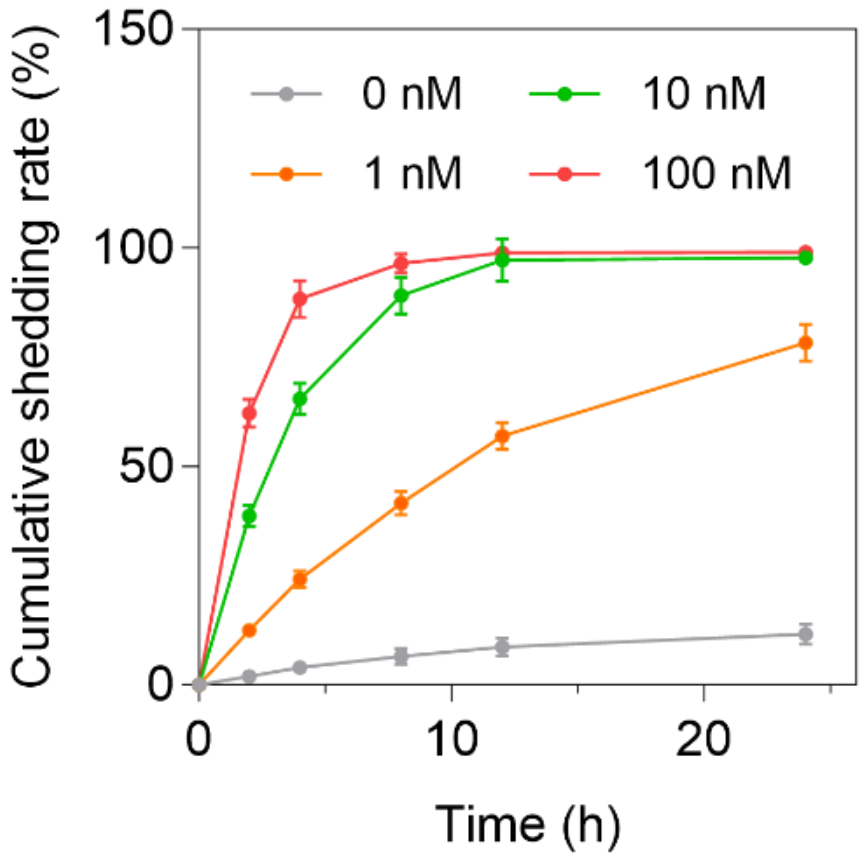

Figure S23. MMP-2 responsive degradation kinetics of the programmable bacterial capsules under gradient enzyme concentrations. Data are presented as mean ± s.d. based on three independent experimental replicates ($n = 3$).

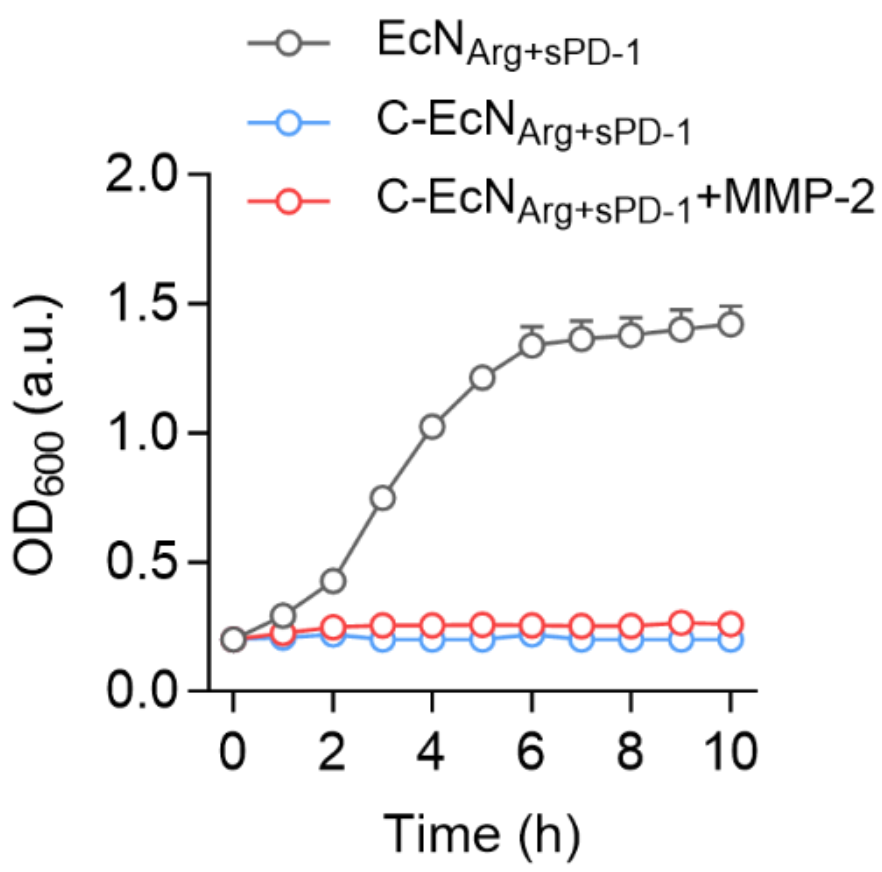

Figure S24. Time-dependent growth curves of $EcN_{Arg+sPD-1}$ and $C\text{-}EcN_{Arg+sPD-1}$ in the presence or absence of MMP-2 (10 nM) cultured at 37°C. Data are presented as means ± s.d. from three independent experiments (n = 3).

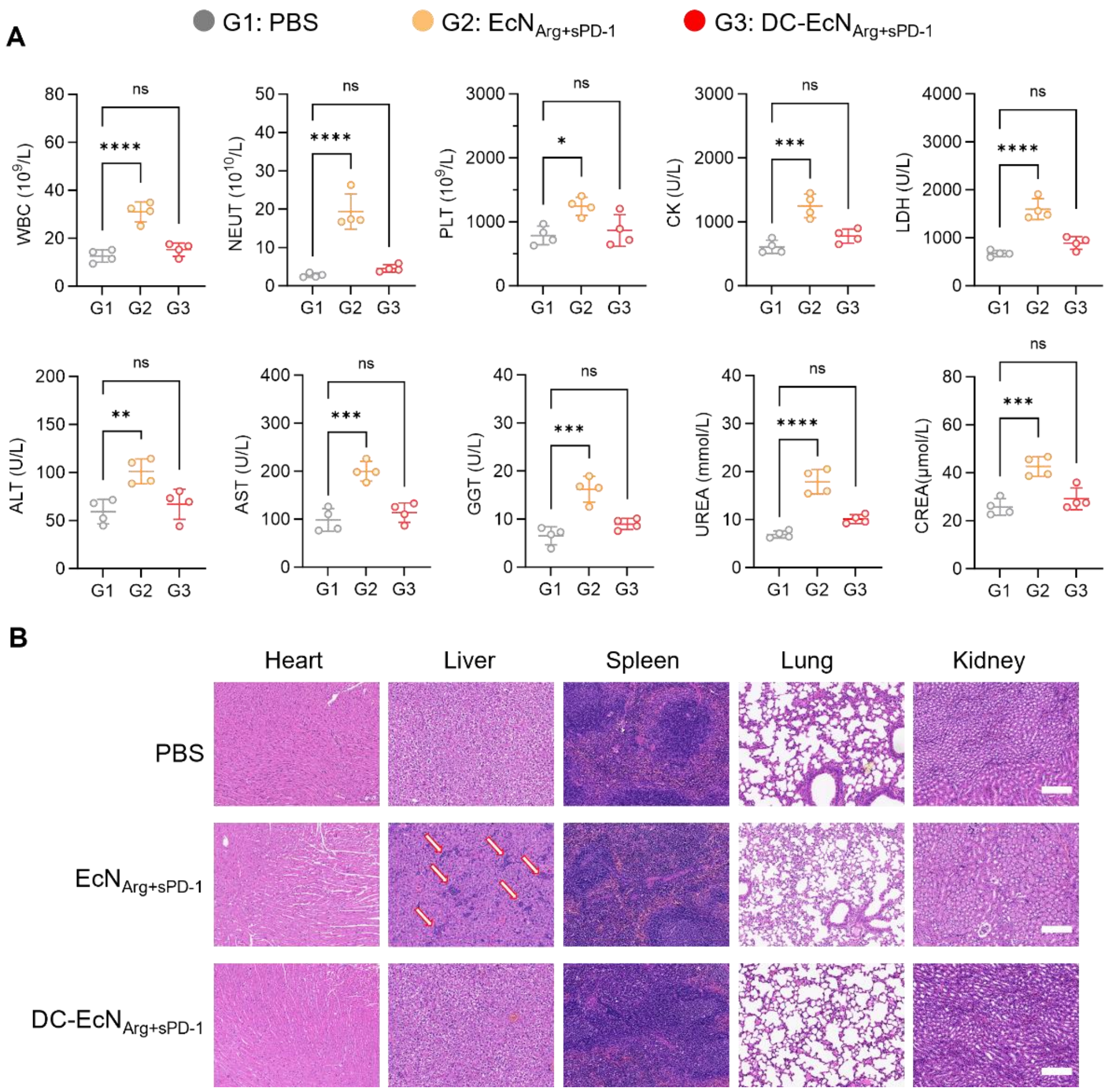

Figure S25. (A) Blood biochemical and routine blood tests after different treatments, including WBC, NEUT, PLT, CK, LDH, ALT, AST, GGT, UREA, and CREA. (B) Representative H&E staining images of major organs (heart, liver, spleen, lung, and kidney) of mice treated with various formulations; Scale bars: 200 µm. Data in (A) are presented as means ± s.d. from four independent experiments (*n* = 4). Statistical analysis in (A) was performed using one-way ANOVA followed by Tukey's test with multiple comparisons. The significant levels are shown as ns > 0.05, *$P < 0.05$, **$P < 0.01$, ***$P < 0.001$, and ****$P < 0.0001$.

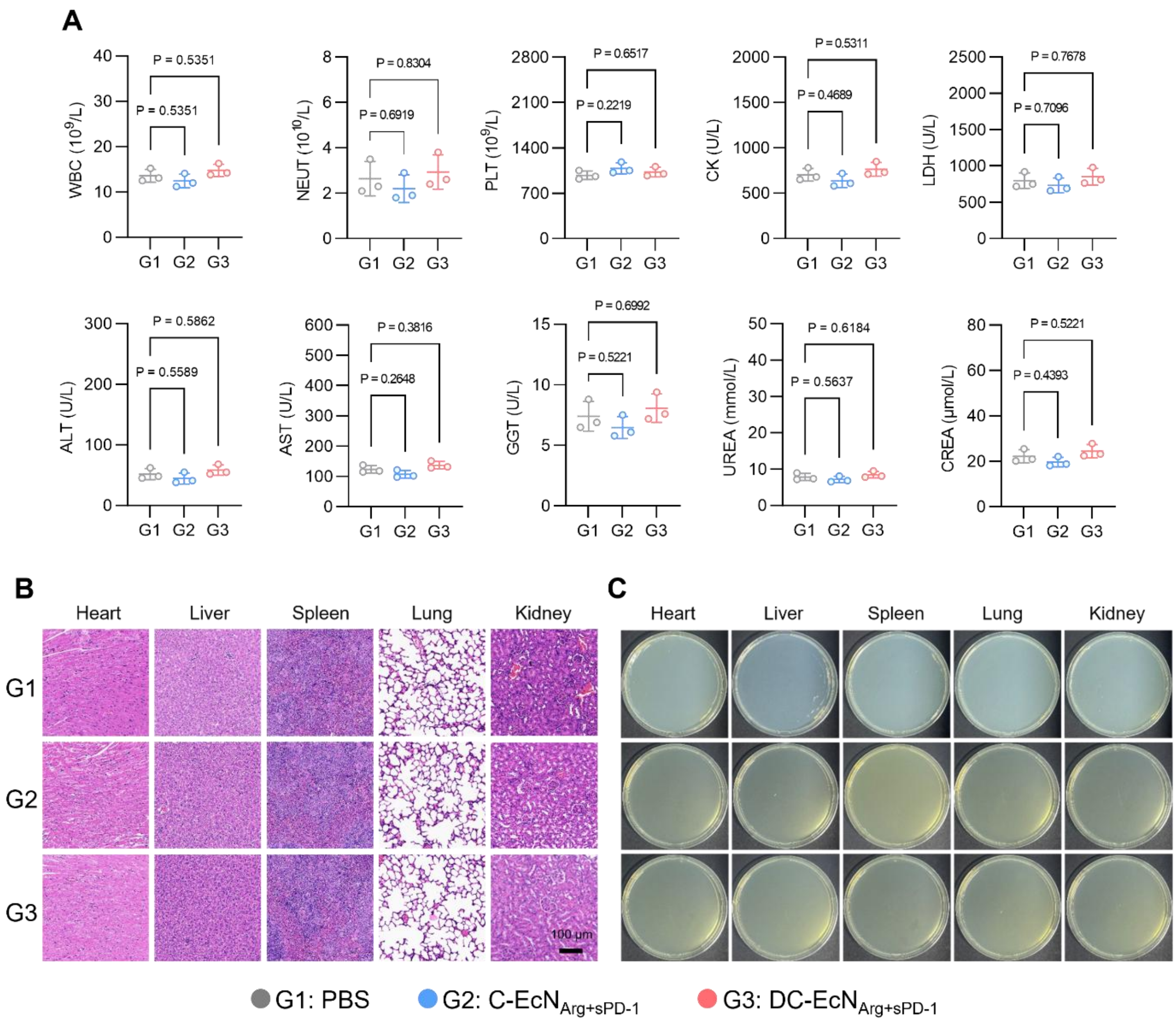

Figure S26. Long-term hematological, blood biochemical, and histopathological biosafety profiling at day 30 post-treatments. (A) Blood biochemical and routine blood tests after different treatments, including WBC, NEUT, PLT, CK, LDH, ALT, AST, GGT, UREA, and CREA. (B) Representative H&E staining images of major organs (heart, liver, spleen, lung, and kidney) of mice treated with various formulations. (C) Representative photographs of LB agar plates spread with tissue homogenate of major organs at day 30 after intravenous injection of PBS, C-EcN$_{Arg+sPD-1}$, and DC-EcN$_{Arg+sPD-1}$. Data are presented as means ± s.d. from three independent experiments (*n* = 3) Statistical analysis was performed using one-way ANOVA followed by Tukey's test with multiple comparisons.

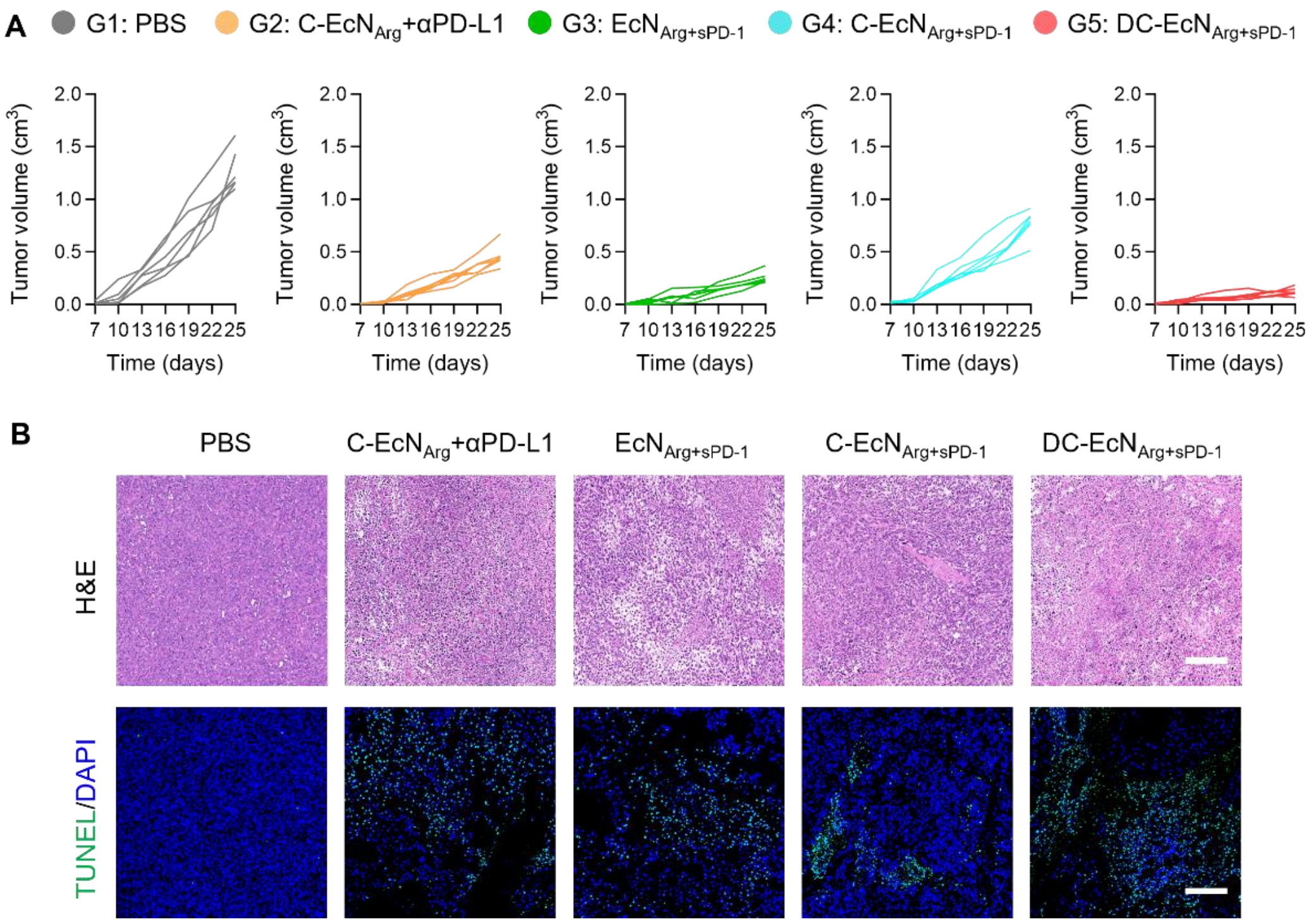

Figure S27. (A) Individual tumor growth curves after different treatments (n = 6). (B) H&E and TUNEL staining of tumor sections from the mice after different treatments; Scale bars: 200 μm.

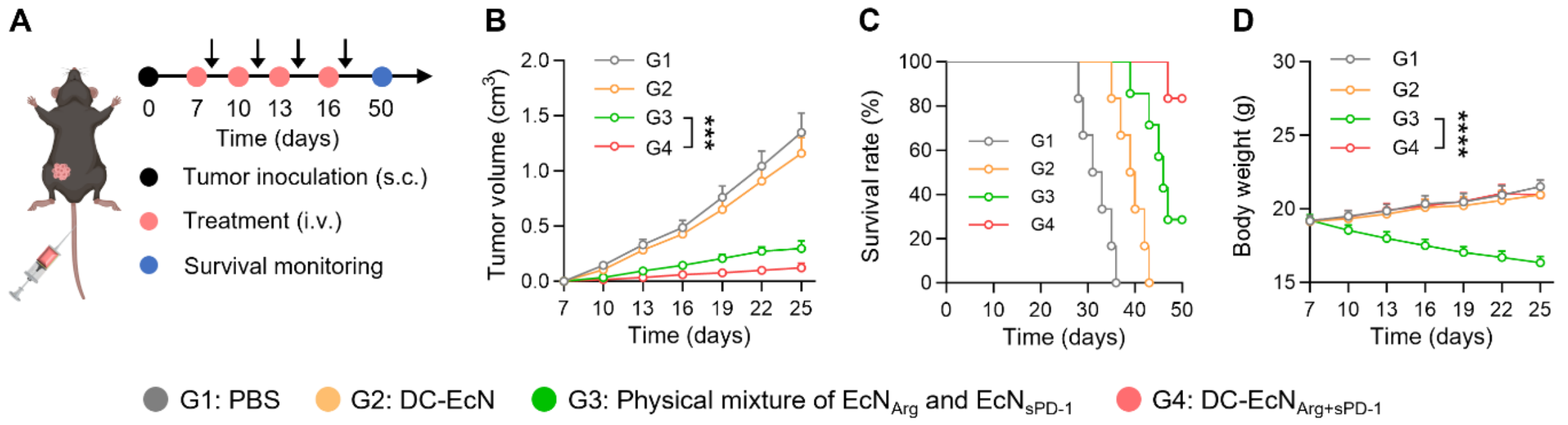

Figure S28. (A) Schematic illustration for evaluating the antitumor effect of various formations in B16F10-bearing mice. (B) Tumor growth curves in B16F10-bearing mice after different treatments. (C) Survival curves of B16F10-bearing mice after different treatments. (D) Body weight changes of B16F10-bearing mice after different treatments. Data are presented as mean ± s.d. based on six independent experimental replicates (*n* = 6). Statistical analysis was performed using two-way ANOVA followed by Sidak's test with multiple comparisons.

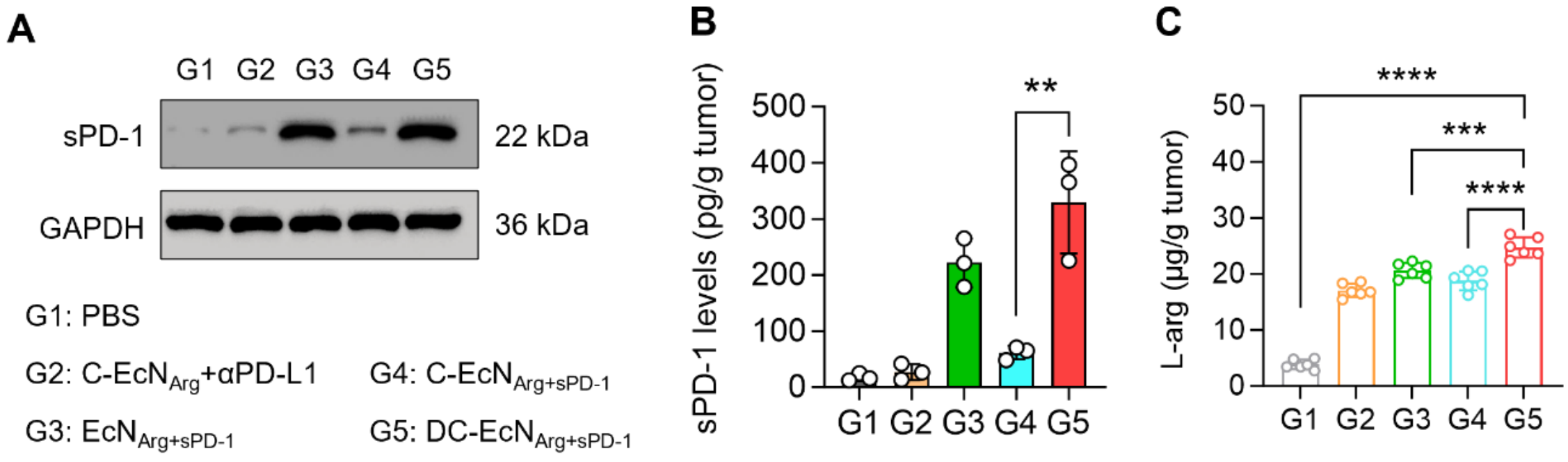

Figure S29. (A) Western blotting analysis of sPD-1 expression in tumor tissues after different treatments. (B) sPD-1 expression in tumor tissues after different treatments. (C) Intratumoral L-arg levels after different treatments. Data in (B) are presented as means ± s.d. from three independent experiments (n = 3). Data in (C) are presented as means ± s.d. from six independent experiments (n = 6). Statistical analysis was performed using one-way ANOVA followed by Tukey's test with multiple comparisons. The significant levels are shown as $^{**}P < 0.01$, $^{***}P < 0.001$ and $^{****}P < 0.0001$.

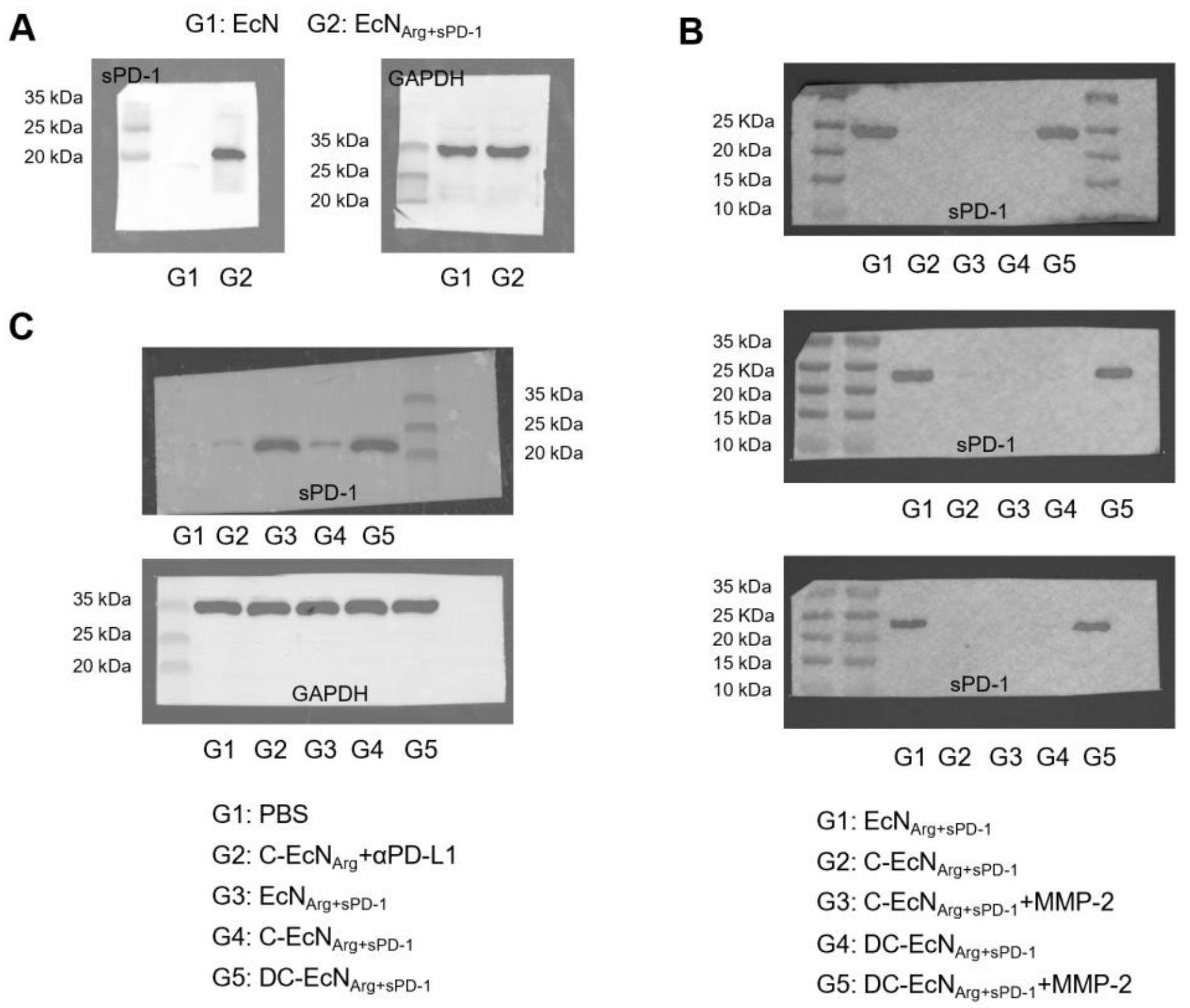

Figure S30. The unprocessed western blot images for Figure 5B (A), Figure 5G (B), and Figure S29A (C).

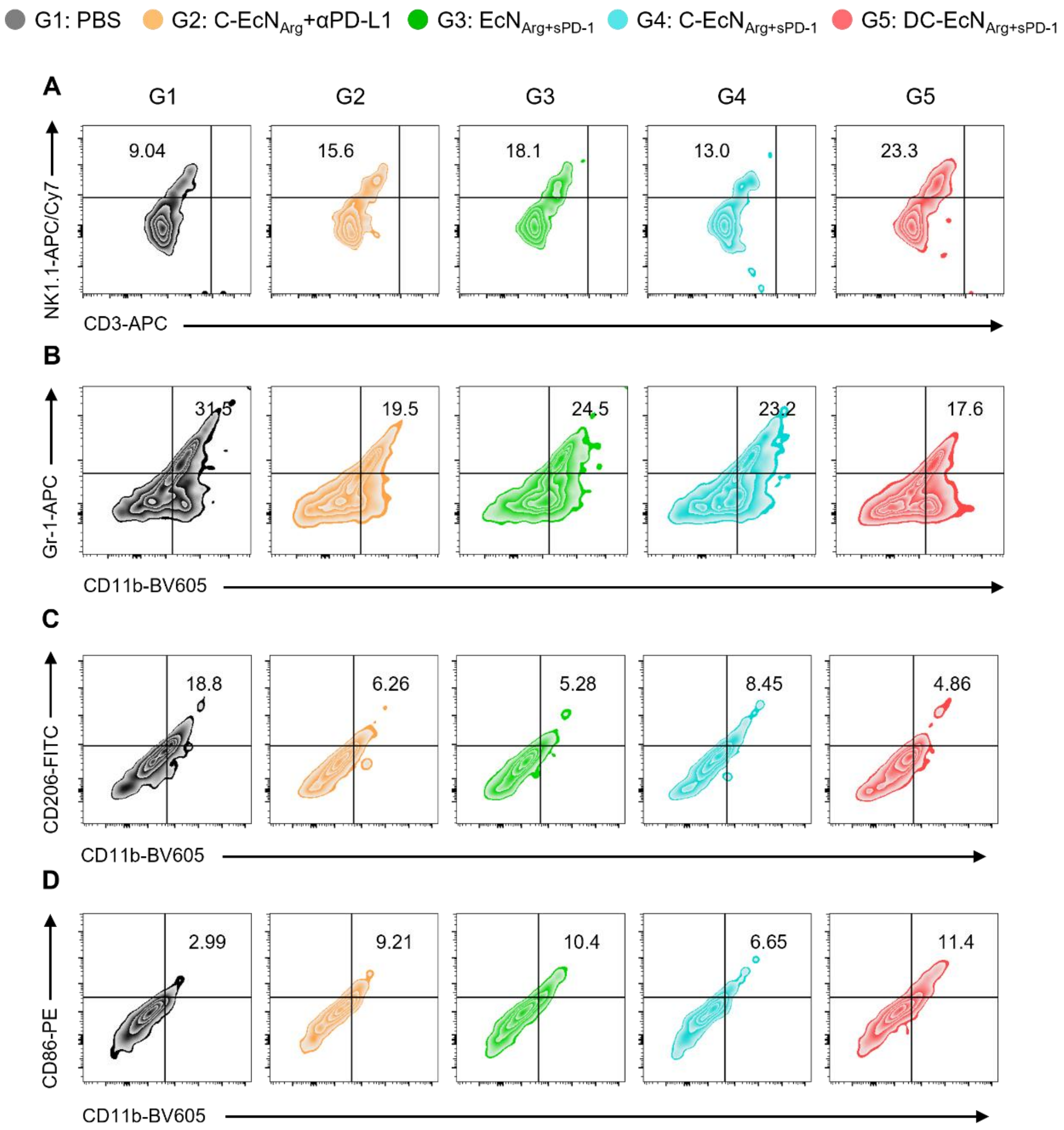

Figure S31. Representative flow cytometric analysis of the tumor infiltration of NK cells (A), MDSCs (B), M2-like (C), and M1-like macrophages (D) after different treatments.

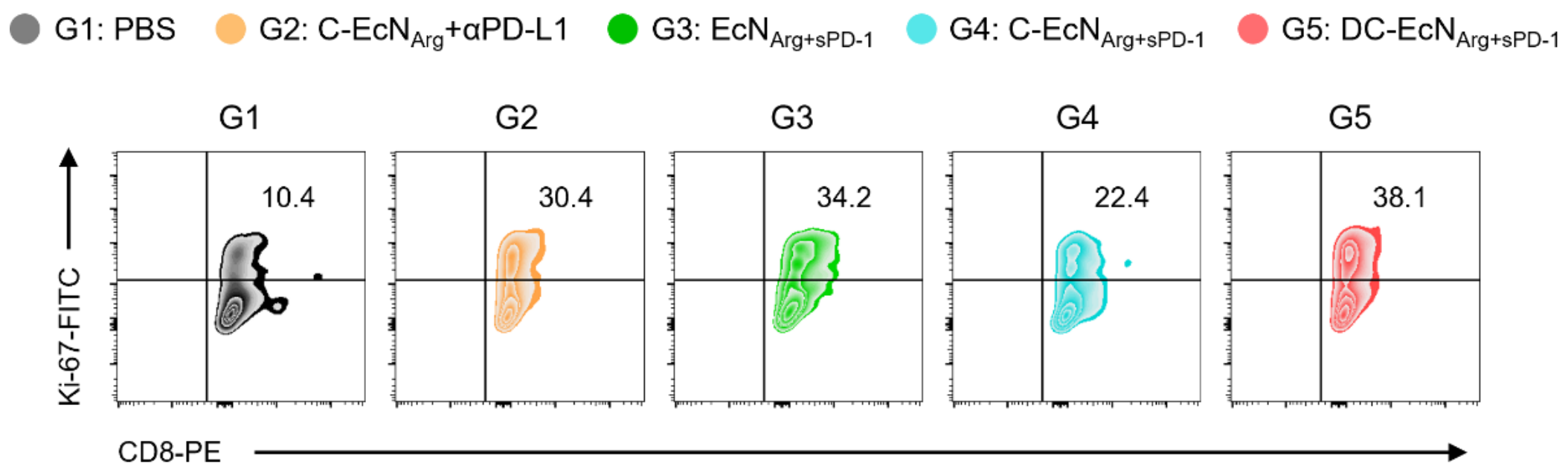

Figure S32. Representative flow cytometric analysis of the tumor infiltration of Ki-67$^+$ CD8$^+$ T cells after different treatments.

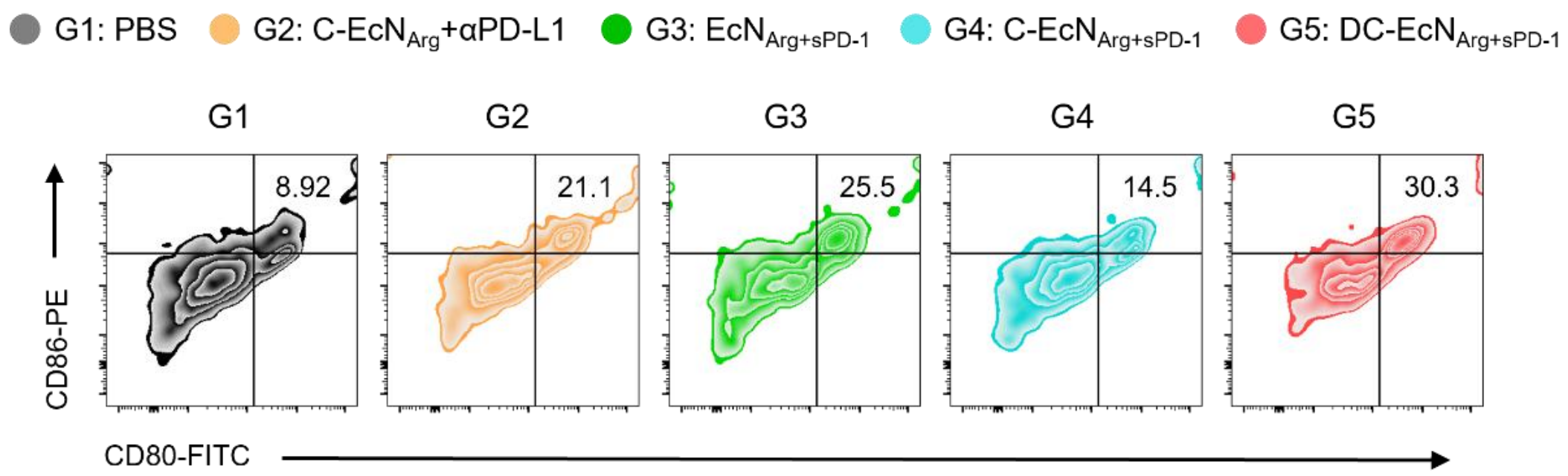

Figure S33. Representative flow cytometric analysis of DCs maturation in LNs after different treatments.

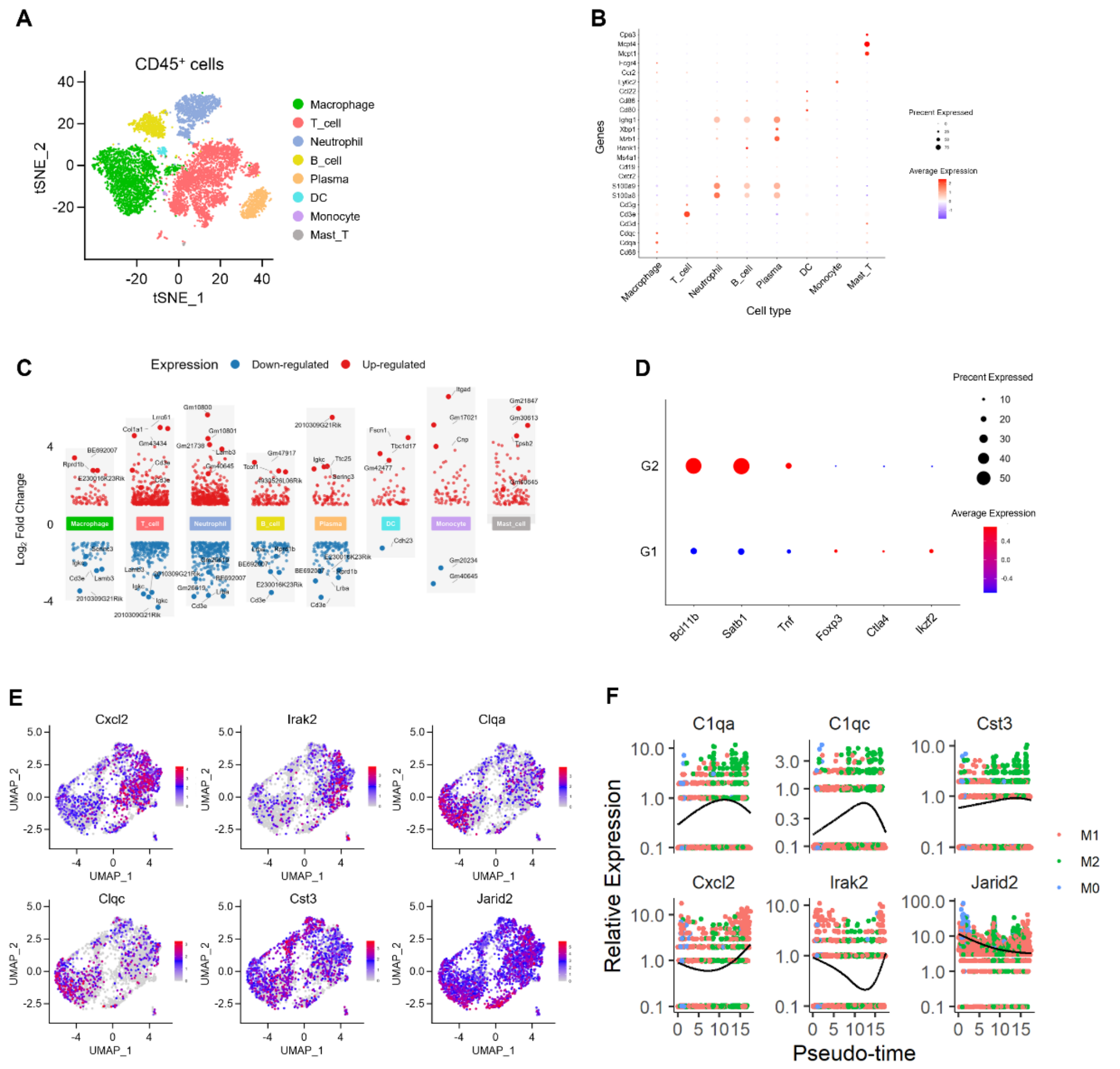

Figure S34. (A) The t-SNE plot for subgrouping analysis of CD45-positive cells in the tumors. (B) Dot plot of normalized expression of macrophage, T cell, neutrophil, B cell, plasma, DC, monocyte, and mast cell marker genes expression. (C) Dot plot of differentially expressed genes among eight cell types in DC-EcN$_{Arg+sPD-1}$-treated tumors compared to that of PBS group. (D) Dot plot of representative gene expression in CD8^{+} T cells (G1: PBS and G2: DC-EcN$_{Arg+sPD-1}$). (E) The UMAP plot of specific marker genes coloured by cluster in the macrophage cluster. (F) The specific marker genes expression in developmental trajectory.

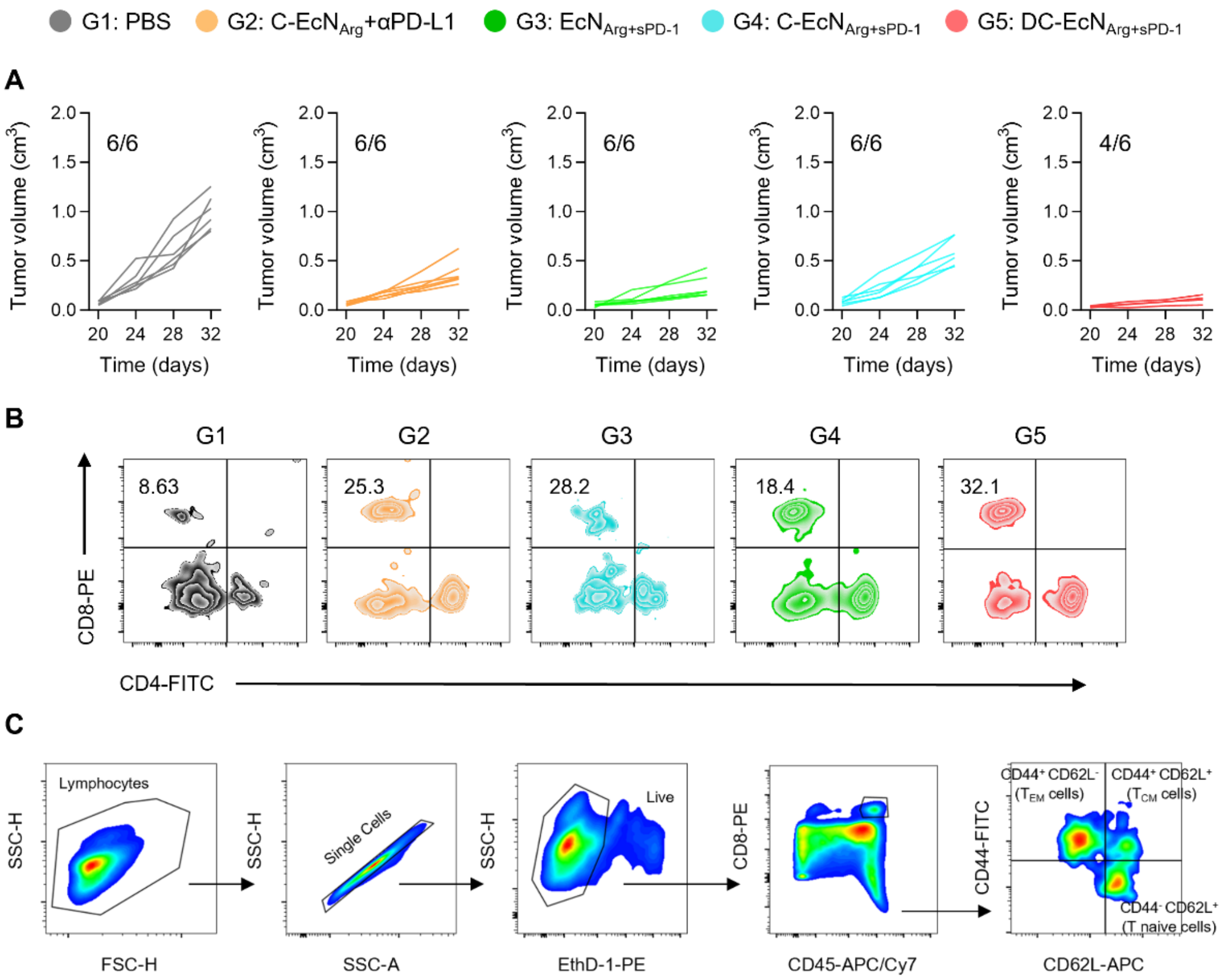

Figure S35. (A) Individual tumor growth curves after different treatments (n = 6). (B) Representative flow cytometric analysis of $CD8^+$ T cells in tumors after different treatments. (C) Flow cytometric gating strategies for the analysis of T_{EM} and T_{CM} in spleens after treatments.

Table S1. Gene sequences.

Gene name	Sequence (5'-3')
ArgA	ATGGTAAAGGAACGTAAAACCGAGTTGGTCGAGGGATTCCGCC<br>ATTCGGTTCCCTGTATCAATACCCACCGGGGAAAAACGTTTGTC<br>ATCATGCTCGGCGGTGAAGCCATTGAGCATGAGAATTTCTCCAG<br>TATCGTTAATGATATCGGGTTGTTGCACAGCCTCGGCATCCGTCT<br>GGTGGTGGTCTATGGCGCACGTCCGCAGATCGACGCAAATCTG<br>GCTGCGCATCACCACGAACCGCTGTATCACAAGAATATACGTGT<br>GACCGACGCCAAAACACTGGAACTGGTGAAGCAGGCTGCGGG<br>AACATTGCAACTGGATATTACTGCTCGCCTGTCGATGAGTCTCA<br>ATAACACGCCGCTGCAGGGCGCGCATATCAACGTCGTCAGTGG<br>CAATTTTATTATTGCCCAGCCGCTGGGCGTCGATGACGGCGTGG<br>ATTACTGCCATAGCGGGCGTATCCGGCGGATTGATGAAGACGCG<br>ATCCATCGTCAACTGGACAGCGGTGCAATAGTGCTAATGGGGCC<br>GGTCGCTGTTTCAGTCACTGGCGAGAGCTTTAACCTGACCTCG<br>GAAGAGATTGCCACTCAACTGGCCATCAAACTGAAAGCTGAAA<br>AGATGATTGGTTTTTGCTCTTCCCAGGGCGTCACTAATGACGAC<br>GGTGATATTGTCTCCGAACTTTTCCCTAACGAAGCGCAAGCGCG<br>GGTAGAAGCCCAGGAAGAGAAAGGCGATTACAACTCCGGTACG<br>GTGCGCTTTTTGCGTGGCGCAGTGAAAGCCTGCCGCAGCGGCG<br>TGCGTCGCTGTCATTTAATCAGTTATCAGGAAGATGGCGCGCTG<br>TTGCAAGAGTTGTTCTCACGCGACGGTATCGGTACGCAGATTGT<br>GATGGAAAGCGCCGAGCAGATTCGTCGCGCAACAATCAACGAT<br>ATTGGCGGTATTCTGGAGTTGATTCGCCCACTGGAGCAGCAAGG<br>TATTCTGGTACGCCGTTCTCGCGAGCAGCTGGAGATGGAAATCG<br>ACAAATTCACCATTATTCAGCGCGATAACACGACTATTGCCTGC<br>GCCGCGCTCTATCCGTTCCCGGAAGAGAAGATTGGGGAAATGG<br>CCTGTGTGGCAGTTCACCCGGATTACCGCAGTTCATCAAGGGGT<br>GAAGTTCTGCTGGAACGCATTGCCGCTCAGGCTAAGCAGAGCG<br>GCTTAAGCAAATTGTTTGTGCTGACCACGCGCAGTATTCACTGG<br>TTCCAGGAACGTGGATTTACCCCAGTGGATATTGATTTACTGCCC<br>GAGAGCAAAAAGCAGTTGTACAACTACCAGCGTAAATCCAAAG<br>TGTTGATGGCGGATTTAGGGTAA
Arg box	ATGCTTTAGACTTGCAAATGAATAATCATCCATATAAATTGAATTT<br>TAATTCATTGAGGCGTTAGCCACAGGAGGGATCTTAA
pelB	ATGAAATACCTATTGCCTACGGCAGCCGCCGGATTGCTATTACTC<br>GCGGCTCAACCCGCAATGGCC
sPD1	ATGTGGGTCCGGCAGGTACCCTGGTCATTCACTTGGGCTGTGCT<br>GCAGTTGAGCTGGCAATCAGGGTGGCTTCTAGAGGTCCCCAAT<br>GGGCCCTGGAGGTCCCTCACCTTCTACCCAGCCTGGCTCACAG<br>TGTCAGAGGGAGCAAATGCCACCTTCACCTGCAGCTTGTCCAA<br>CTGGTCGGAGGATCTTATGCTGAACTGGAACCGCCTGAGTCCC<br>AGCAACCAGACTGAAAAACAGGCCGCCTTCTGTAATGGTTTGA<br>GCCAACCCGTCCAGGATGCCCGCTTCCAGATCATACAGCTGCCC<br>AACAGGCATGACTTCCACATGAACATCCTTGACACACGGCGCA<br>ATGACAGTGGCATCTACCTCTGTGGGGCCATCTCCCTGCACCCC<br>AAGGCAAAAATCGAGGAGAGCCCTGGAGCAGAGCTCGTGGTA<br>ACAGAGAGAATCCTGGAGACCTCAACAAGATATCCCAGCCCCT<br>CGCCCAAACCAGAAGGCCGGTTTCAAGGCATGTGA
